# Community estimate of global glacier mass changes from 2000 to 2023

**DOI:** 10.1038/s41586-024-08545-z

**Published:** 2025-02-19

**Authors:** The GlaMBIE Team, Michael Zemp, Livia Jakob, Inés Dussaillant, Samuel U. Nussbaumer, Noel Gourmelen, Sophie Dubber, Geruo A, Sahra Abdullahi, Liss Marie Andreassen, Etienne Berthier, Atanu Bhattacharya, Alejandro Blazquez, Laura F. Boehm Vock, Tobias Bolch, Jason Box, Matthias H. Braun, Fanny Brun, Eric Cicero, William Colgan, Nicolas Eckert, Daniel Farinotti, Caitlyn Florentine, Dana Floricioiu, Alex Gardner, Christopher Harig, Javed Hassan, Romain Hugonnet, Matthias Huss, Tómas Jóhannesson, Chia-Chun Angela Liang, Chang-Qing Ke, Shfaqat Abbas Khan, Owen King, Marin Kneib, Lukas Krieger, Fabien Maussion, Enrico Mattea, Robert McNabb, Brian Menounos, Evan Miles, Geir Moholdt, Johan Nilsson, Finnur Pálsson, Julia Pfeffer, Livia Piermattei, Stephen Plummer, Andreas Richter, Ingo Sasgen, Lilian Schuster, Thorsten Seehaus, Xiaoyi Shen, Christian Sommer, Tyler Sutterley, Désirée Treichler, Isabella Velicogna, Bert Wouters, Harry Zekollari, Whyjay Zheng

**Affiliations:** 1 Department of Geography, University of Zurich, Zurich, Switzerland; 2 Earthwave Ltd, Edinburgh, UK; 3 University of Edinburgh, School of Geosciences, Edinburgh, UK; 4 Department of Earth System Science, University of California, Irvine, Irvine, CA USA; 5 German Aerospace Center (DLR), Earth Observation Center, Oberpfaffenhofen, Germany; 6 Norwegian Water Resources and Energy Directorate (NVE), Oslo, Norway; 7 Université de Toulouse, LEGOS (CNES/CNRS/IRD/UT3), Toulouse, France; 8 Department of Earth Sciences and Remote Sensing, JIS University, Kolkata, India; 9 Centre for Data Science, JIS Institute of Advanced Studies and Research, Kolkata, India; 10 Department of Mathematics, Statistics, and Computer Science, St. Olaf College, Northfield, MN USA; 11 Institute of Geodesy, Graz University of Technology, Graz, Austria; 12 Central-Asian Regional Glaciological Centre of Category 2 Under the Auspices of UNESCO, Almaty, Kazakhstan; 13 Department of Glaciology and Climate, Geological Survey of Denmark and Greenland, Copenhagen, Denmark; 14 Institute of Geography, Friedrich-Alexander-Universität Erlangen-Nürnberg, Erlangen, Germany; 15 Institut des Géosciences de l’Environnement, Université Grenoble-Alpes, CNRS, IRD, Grenoble, France; 16 Department of Geosciences, University of Arizona, Tucson, AZ USA; 17 Department of Earth and Space Science and Engineering, York University, Toronto, Ontario Canada; 18 INRAE, CNRS, IRD, Grenoble INP, IGE, Grenoble, France; 19 Grenoble Risk Institute, Grenoble, France; 20 Laboratory of Hydraulics, Hydrology and Glaciology (VAW), ETH Zurich, Zurich, Switzerland; 21 Swiss Federal Institute for Forest, Snow and Landscape Research (WSL), Birmensdorf, Switzerland; 22 Northern Rocky Mountain Science Center, US Geological Survey, Bozeman, MT USA; 23 Jet Propulsion Laboratory, California Institute of Technology, Pasadena, CA USA; 24 DTU Space, Technical University of Denmark, Kongens Lyngby, Denmark; 25 University of Washington, Civil and Environmental Engineering, Seattle, WA USA; 26 Department of Geosciences, University of Fribourg, Fribourg, Switzerland; 27 Icelandic Meteorological Office, Reykjavik, Iceland; 28 Jiangsu Provincial Key Laboratory of Geographic Information Science and Technology, Key Laboratory for Land Satellite Remote Sensing Applications of Ministry of Natural Resources, School of Geography and Ocean Science, Nanjing University, Nanjing, China; 29 Department of Geography, Politics and Sociology, Newcastle University, Newcastle, UK; 30 Department of Atmospheric and Cryospheric Sciences, University of Innsbruck, Innsbruck, Austria; 31 School of Geographical Sciences, University of Bristol, Bristol, UK; 32 School of Geography and Environmental Sciences, Ulster University, Coleraine, UK; 33 Geological Survey of Canada Pacific, Natural Resources Canada, Sidney, British Columbia Canada; 34 Hakai Institute, Campbell River, British Columbia Canada; 35 Geography Earth and Environmental Sciences, University of Northern British Columbia, Prince George, British Columbia Canada; 36 Norwegian Polar Institute, Tromsø, Norway; 37 Institute of Earth Sciences, University of Iceland, Reykjavik, Iceland; 38 Magellium, Ramonville-Saint-Agne, France; 39 Department of Geosciences, University of Oslo, Oslo, Norway; 40 European Space Agency, ESRIN, Frascati, Italy; 41 Laboratorio MAGGIA, Facultad de Ciencias Astronómicas y Geofísicas, Universidad Nacional de La Plata, La Plata, Argentina; 42 Consejo Nacional de Investigaciones Científicas y Técnicas, Buenos Aires, Argentina; 43 Alfred Wegener Institute, Helmholtz Centre for Polar and Marine Research, Bremerhaven, Germany; 44 Institute of Geography, University of Augsburg, Augsburg, Germany; 45 School of Earth Sciences and Engineering, Hohai University, Nanjing, China; 46 Polar Science Center, Applied Physics Laboratory, University of Washington, Seattle, WA USA; 47 Department of Geoscience and Remote Sensing, Delft University of Technology, Delft, The Netherlands; 48 Department of Water and Climate, Vrije Universiteit Brussel, Brussels, Belgium; 49 Center for Space and Remote Sensing Research, National Central University, Taoyuan, Taiwan

**Keywords:** Cryospheric science, Climate-change impacts, Hydrology

## Abstract

Glaciers are indicators of ongoing anthropogenic climate change^1^. Their melting leads to increased local geohazards^2^, and impacts marine^3^ and terrestrial^4,5^ ecosystems, regional freshwater resources^6^, and both global water and energy cycles^7,8^. Together with the Greenland and Antarctic ice sheets, glaciers are essential drivers of present^9,10^ and future^11–13^ sea-level rise. Previous assessments of global glacier mass changes have been hampered by spatial and temporal limitations and the heterogeneity of existing data series^14–16^. Here we show in an intercomparison exercise that glaciers worldwide lost 273 ± 16 gigatonnes in mass annually from 2000 to 2023, with an increase of 36 ± 10% from the first (2000–2011) to the second (2012–2023) half of the period. Since 2000, glaciers have lost between 2% and 39% of their ice regionally and about 5% globally. Glacier mass loss is about 18% larger than the loss from the Greenland Ice Sheet and more than twice that from the Antarctic Ice Sheet^17^. Our results arise from a scientific community effort to collect, homogenize, combine and analyse glacier mass changes from in situ and remote-sensing observations. Although our estimates are in agreement with findings from previous assessments^14–16^ at a global scale, we found some large regional deviations owing to systematic differences among observation methods. Our results provide a refined baseline for better understanding observational differences and for calibrating model ensembles^12,16,18^, which will help to narrow projection uncertainty for the twenty-first century^11,12,18^.

## Abstract (web summary)

An intercomparison exercise reassesses mass loss from glaciers worldwide based on the main in situ and satellite methods from 2000 to 2023; the results are consistent with previous assessments and provide a refined and comprehensive observational baseline for future impact and modelling studies.

## Main

Glaciers separate from the continental ice sheets in Greenland and Antarctica covered a global area of approximately 706,000 km^2^ around the year 2000^19^, with an estimated total volume of 158,170 ± 41,030 km^3^, equivalent to a potential sea-level rise of 324 ± 84 mm (ref. ^20^). Glaciers are integral components of Earth’s climate and hydrologic system^1^. Hence, glacier monitoring is essential for understanding and assessing ongoing changes^21,22^, providing a basis for impact^2–10^ and modelling^11–13^ studies, and helping to track progress on limiting climate change^23^. The four main observation methods to derive glacier mass changes include glaciological measurements, digital elevation model (DEM) differencing, altimetry and gravimetry. Additional concepts include hybrid approaches that combine different observation methods. In situ glaciological measurements have been carried out at about 500 unevenly distributed glaciers^24^, representing less than 1% of Earth’s glaciers^19^. Glaciological time series provide seasonal-to-annual variability of glacier mass changes^25^. Although these are generally well correlated regionally, long-term trends of individual glaciers might not always be representative of a given region. Spaceborne observations complement in situ measurements, allowing for glacier monitoring at global scale over recent decades. Several optical and radar sensors allow the derivation of DEMs, which reflect the glacier surface topography. Repeat mapping and calculation of DEM differences provide multi-annual trends in elevation and volume changes^26^ for all glaciers in the world^27^. Similarly, laser and radar altimetry determine elevation changes along linear tracks, which can be extrapolated to calculate regional estimates of glacier elevation and volume change^28^. Unlike DEM differencing, altimetry provides spatially sparse observations but has a high (that is, monthly to annual) temporal resolution^26^. DEM differencing and altimetry require converting glacier volume to mass changes using density assumptions^29^. Satellite gravimetry estimates regional glacier mass changes at monthly resolution by measuring changes in Earth’s gravitational field after correcting for solid Earth and hydrological effects^30,31^. Although satellite gravimetry provides high temporal resolution and direct estimates of mass, it has a spatial resolution of a few hundred kilometres, which is several orders of magnitude lower than DEM differencing or altimetry^26^.

The heterogeneity of these observation methods in terms of spatial, temporal and observational characteristics, the diversity of approaches within a given method, and the lack of homogenization challenged past assessments of glacier mass changes. In the Intergovernmental Panel on Climate Change (IPCC)’s Sixth Assessment Report (AR6)^16^, for example, glacier mass changes for the period from 2000 to 2019 relied on DEM differencing from a limited number of global^27^ and regional studies^16^. Results from a combination of glaciological and DEM differencing^25^ as well as from gravimetry^30^ were used for comparison only. The report calculated regional estimates over a specific baseline period (2000–2019) and as mean mass-change rates based on selected studies per region, which only partly considered the strengths and limitations of the different observation methods.

The spread of reported results—many outside uncertainty margins—and recent updates from different observation methods afford an opportunity to assess regional and global glacier mass loss with a community-led effort. Within the Glacier Mass Balance Intercomparison Exercise (GlaMBIE; https://glambie.org), we collected, homogenized and combined regional results from the observation methods described above to yield a global assessment towards the upcoming IPCC reports of the seventh assessment cycle. At the same time, GlaMBIE provides insights into regional trends and interannual variabilities, quantifies the differences among observation methods, tracks observations within the range of projections, and delivers a refined observational baseline for future impact and modelling studies.

## Glacier mass balance intercomparison

**Extended Data Fig. 1:**
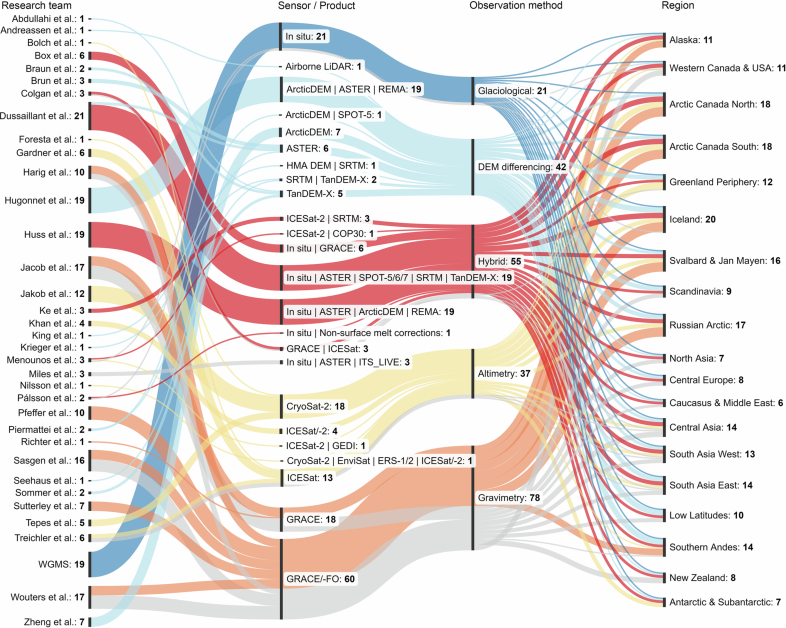
Data submissions to the intercomparison exercise. The figure provides an overview of research teams (left, in alphabetic order) participating in GlaMBIE with their selection of sensors or products (middle left) used for computing glacier mass-change estimates from different observation methods (middle right) for the 19 regions (right). From the 233 regional results, 195 (coloured lines) were used to compute our combined estimates, and 38 (grey lines) were excluded based on regional confidence levels of observation methods (see Methods). Colours follow observation methods. A more detailed overview of all data contributions is given in Supplementary Information Tables 1 and 2. The figure was produced with SankeyMATIC^113^. Source Data

**Extended Data Fig. 2:**
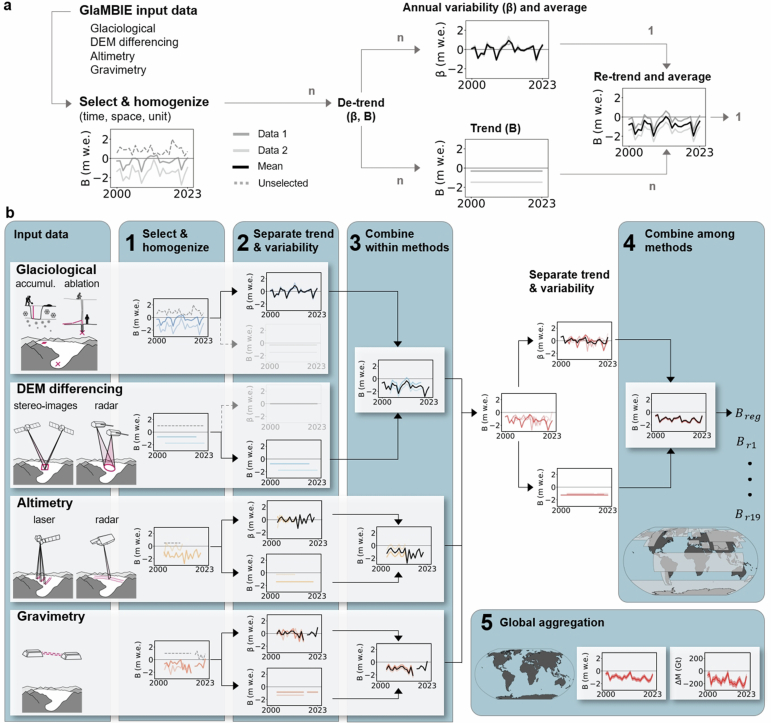
Principal approach and workflow of the intercomparison exercise. The schematic diagram illustrates the principal approach (**a**) to combine input data from different observational sources. After selection and homogenization, each dataset is separated into its annual variability (β) and long-term trend $(\bar{B})$ by de-trending. The annual variabilities from multiple input data are averaged to one time series and added to each long-term trend. The new set of re-trended time series are averaged to one combined estimate. In the GlaMBIE workflow (**b**), this approach is applied to combine (i) the annual variability from glaciological observations with long-term trends from DEM differencing, (ii) multiple input data from altimetry, (iii) multiple input data from gravimetry, and finally to (iv) combine these three results among the different observation methods. Finally, the region estimates are corrected from hydrological to calendar years and for regional area changes and cumulated to a global estimate. More details on the approach are provided in the Methods.

For 19 predefined regions, we compiled 233 estimates of regional glacier mass changes from about 450 data contributors organized in 35 research teams (Extended Data Fig. 1 and Supplementary Tables 1 and 2). These estimates originate from one or more of the four observation methods and cover the period since the early 2000s. The glaciological method and DEM differencing yield results for individual glaciers with annual and decadal resolution, respectively, whereas current estimates from altimetry and gravimetry are available only on a regional scale but provide monthly resolution. Similarly, DEM differencing and altimetry observe elevation change, whereas the glaciological method and gravimetry provide changes in glacier mass. To account for the strengths and limitations of the different methods, we collected all regional datasets in the native units (that is, metres (m), metre water equivalent (m w.e.) and gigatonnes (Gt)) and at the temporal resolutions specified by the participants (that is, monthly, annual and multi-annual). After quality control and expert evaluation of the input data by the GlaMBIE community (that is, co-authors and data contributors), we combined the selected estimates using a five-step approach (Methods and Extended Data Fig. 2). First, we homogenized all datasets concerning spatial, temporal and unit domains using common conversions. Second, we separated the temporal variability from the long-term trend for each dataset. Third, we combined the average temporal variability and long-term trends for each region. Depending on available input data, we computed three regional time series: one for altimetry, one for gravimetry and one combining the temporal variability from the glaciological data with the long-term trends from DEM differencing. Fourth, we combined the time series from altimetry and gravimetry and the combination of glaciological and DEM differencing into single regional estimates. Fifth, we summed the regional estimates to a global time series. With this approach, GlaMBIE provides a time series of annual mass changes at regional and global scales from 2000 to 2023, homogenized consistently for differences in space, time and unit characteristics, and accounting for regional glacier area changes (Methods). Uncertainties originate from several sources that are assumed to be independent, including the input data’s reported uncertainties, homogenization corrections and the spread among the input data (Methods). Our assessment does not correct systematic errors^26,32^, owing to the lack of reference data to perform such corrections at the regional scale. We, instead, excluded individual estimates based on expert evaluation by the GlaMBIE community (Methods). Similar reasons also prevented us from validating our reported random errors. Many input data originate from the same sources or are obtained from similar methods and are thus likely to have correlated errors. As true independence among these mass-change estimates is unlikely, our random errors probably represent a lower range of the actual uncertainty for a given region. Finally, as for previous efforts, our estimates suffer from the limited knowledge of density conversion at short timescales (less than 5 years)^29^.

## Global and regional mass changes

**Fig. 1:**
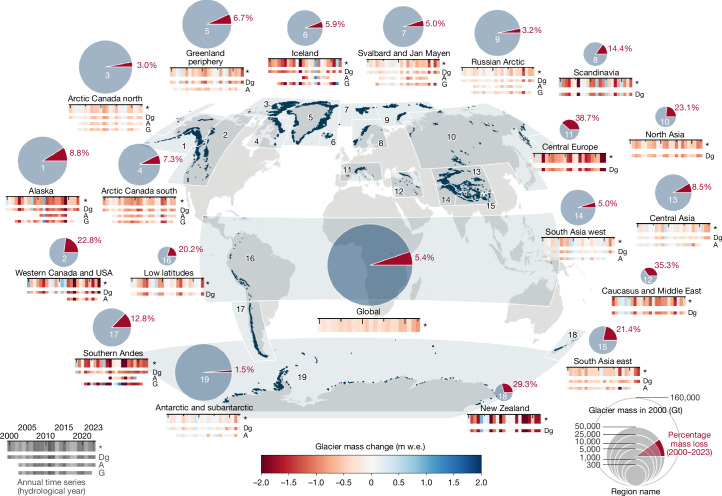
Global glacier mass changes from 2000 to 2023. Regional and global glacier mass changes from 2000 to 2023 as percentage loss (red slice in the pie chart) based on the glacier mass in 2000 (size of the pie chart). The coloured stripes under each pie chart represent annual specific mass changes (in metre water equivalent) for our combined estimate (indicated with an asterisk) together with combined results from DEM differencing and glaciological observations (Dg), altimetry (A) and gravimetry (G). Regional results are represented for hydrological years, that is, running from 1 October to 30 September in the Northern Hemisphere, 1 April to 31 March in the Southern Hemisphere and over the calendar year in the low latitudes. Global results are aggregated for calendar years. Source Data

From 2000 to 2023, the global glacier mass-change (relevant to sea-level rise) totals −6,542 ± 387 Gt (1 Gt = 10^12^ kg; Fig. 1). This loss contributes 18 ± 1 mm to global sea-level rise at an average annual change rate of −273 ± 16 Gt yr^−1^ or 0.75 ± 0.04 mm yr^−1^ (Table 1). Glacier mass-loss rates increased by 36 ± 10% between the first (2000–2011) and the second (2012–2023) half of the record, from −231 ± 23 Gt yr^−1^ to −314 ± 23 Gt yr^−1^. The last pentad (2019–2023) includes the 4 years with the largest annual ice loss of more than 400 Gt yr^−1^, including a record mass loss of 548 ± 120 Gt yr^−1^ (or 1.51 ± 0.33 mm yr^−1^) in 2023. Compared with recent estimates^17^ for the ice sheets from 2002 to 2021, glacier mass loss is about 18% (significant at the 90% confidence interval) larger than the loss from the Greenland Ice Sheet and more than twice the loss from the Antarctic Ice Sheet (Extended Data Table 1). The largest contributors to observed global mean sea-level rise (2003–2016: 3.64 ± 0.26 mm yr^−1^)^33^ include the steric components (2003–2016: 1.19 ± 0.17 mm yr^−1^, 33%)^33^, owing to changes in ocean temperature and salinity, glaciers (this study, 2002–2021: 0.72 ± 0.04 mm yr^−1^, 20%), and the Greenland Ice Sheet (2002–2021: 0.62 ± 0.06 mm yr^−1^, 17%)^17^. Smaller contributions originate from changes in land water storage and the Antarctic Ice Sheet. We note that our glacier mass-change estimate includes calving owing to ice discharge, which is implicitly accounted for in DEM differencing, altimetry and gravimetry. Mass loss owing to calving-front retreat of marine and lake-terminating glaciers^34,35^ is not included, however, as that does not significantly impact global sea level. For the past two decades, mass loss owing to calving-front retreat was quantified as 10 ± 3 Gt yr^−1^ for Northern Hemisphere glaciers^34^ and 43 Gt yr^−1^ for the Greenland Ice Sheet^36^, for comparison.

**Table 1:** Regional and global glacier mass changes from 2000 to 2023

Region	Area in 2000 (km^2^)	Mass in 2000 (Gt)	Observation methods (g | D | A | G | H)	Specific mass-change rate (m w.e. yr^−1^)	Mass-change rate (Gt yr^−1^)	Cumulative sea-level equivalent (mm)
01 Alaska	90,055 ± 4,503	16,497 ± 4,190	1 | 1 | 1 | 5 | 3	−0.72 ± 0.08	−60.8 ± 6.6	4.03 ± 0.44
02 Western Canada and USA	14,602 ± 730	950 ± 243	1 | 1 | 1 | 0 | 3	−0.68 ± 0.06	−9.0 ± 0.9	0.60 ± 0.06
03 Arctic Canada north	105,037 ± 5,252	24,105 ± 6,264	1 | 2 | 4 | 7 | 4	−0.29 ± 0.02	−30.5 ± 2.6	2.02 ± 0.17
04 Arctic Canada south	40,888 ± 2,044	7648 ± 1,981	1 | 2 | 4 | 7 | 4	−0.57 ± 0.05	−23.1 ± 2.1	1.53 ± 0.14
05 Greenland periphery	89,717 ± 4,486	12,525 ± 3,249	1 | 3 | 5 | 0 | 2	−0.44 ± 0.08	−35.1 ± 7.1	2.32 ± 0.47
06 Iceland	11,020 ± 551	3,372 ± 880	1 | 4 | 3 | 7 | 4	−0.79 ± 0.11	−8.3 ± 1.3	0.55 ± 0.08
07 Svalbard and Jan Mayen	34,489 ± 1,724	6,507 ± 1,675	1 | 2 | 3 | 7 | 3	−0.41 ± 0.04	−13.7 ± 1.4	0.91 ± 0.10
08 Scandinavia	2,965 ± 148	275 ± 72	1 | 3 | 0 | 0 | 2	−0.58 ± 0.03	−1.7 ± 0.1	0.11 ± 0.01
09 Russian Arctic	51,633 ± 2,582	11,934 ± 3,095	1 | 3 | 3 | 7 | 3	−0.32 ± 0.04	−16.1 ± 2.1	1.06 ± 0.14
10 North Asia	2,493 ± 125	137 ± 36	1 | 1 | 0 | 0 | 2	−0.56 ± 0.06	−1.3 ± 0.1	0.09 ± 0.01
11 Central Europe	2,150 ± 108	122 ± 27	1 | 2 | 0 | 0 | 2	−1.06 ± 0.04	−2.0 ± 0.1	0.13 ± 0.01
12 Caucasus and Middle East	1,286 ± 64	50 ± 18	1 | 1 | 0 | 0 | 2	−0.62 ± 0.04	−0.7 ± 0.1	0.05 ± 0.00
13 Central Asia	49,747 ± 2,487	2,946 ± 765	1 | 2 | 2 | 0 | 3	−0.22 ± 0.06	−10.4 ± 2.7	0.69 ± 0.18
14 South Asia west	33,568 ± 1,678	2,583 ± 666	1 | 2 | 2 | 0 | 3	−0.17 ± 0.05	−5.4 ± 1.6	0.36 ± 0.10
15 South Asia east	14,942 ± 747	816 ± 207	1 | 3 | 2 | 0 | 3	−0.52 ± 0.08	−7.3 ± 1.2	0.48 ± 0.08
16 Low latitudes	2,369 ± 118	90 ± 27	1 | 4 | 0 | 0 | 2	−0.38 ± 0.06	−0.8 ± 0.1	0.05 ± 0.01
17 Southern Andes	29,429 ± 1471	4,772 ± 1,242	1 | 4 | 1 | 5 | 2	−0.93 ± 0.22	−26.5 ± 6.5	1.68 ± 0.41
18 New Zealand	986 ± 49	66 ± 19	1 | 1 | 0 | 0 | 2	−0.96 ± 0.08	−0.8 ± 0.1	0.05 ± 0.01
19 Antarctic and subantarctic islands	127,845 ± 6,392	26,336 ± 6,734	1 | 1 | 3 | 0 | 2	−0.14 ± 0.06	−16.9 ± 8.2	1.07 ± 0.52
Global total	705,221 ± 11,631	121,728 ± 11,509	19 | 42 | 34 | 45 | 51	−0.41 ± 0.02	−273 ± 16	18.05 ± 1.07

Regional and global glacier area^19^ and mass^20^ corrected to the year 2000 (Methods), with mass changes from 2000 to 2023 expressed as mean specific mass-change rates (m w.e. yr^−1^), mean mass-change rates (Gt yr^−1^) and corresponding equivalents of cumulative global mean sea-level rise (mm). Regional and global changes refer to hydrological and calendar years, respectively. The number of datasets used for the combined estimates is indicated for the different observation methods (Extended Data Fig. 1), including glaciological (g), DEM differencing (D), altimetry (A), gravimetry (G) and hybrid (H). Uncertainties correspond to 95% confidence intervals (Methods).

**Fig. 2:**
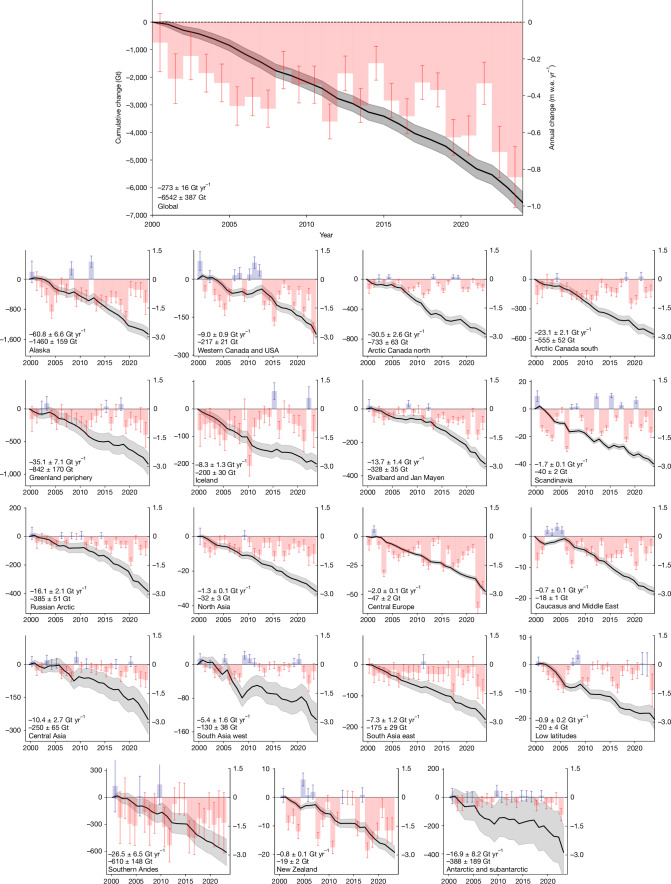
Annual and cumulative glacier mass change from 2000 to 2023. Cumulative and annual glacier mass changes since 2000 for the 19 glacier regions (hydrological years) and aggregated to global sums (calendar years). Cumulative mass changes (left *y* axis, Gt) are shown as black curves, with values for mean annual change rate (Gt yr^−1^) and cumulative change (Gt) for the entire period given in the bottom left corner. Annual mass changes (right *y* axis, m w.e. yr^−1^) are coloured in blue and red for years with positive and negative mass changes, respectively. Uncertainties are given as 95% confidence intervals. It is noted that the left *y* axis differs for each subplot whereas the right *y* axis is the same for all regions. Source Data

All 19 regions experienced glacier mass loss from 2000 to 2023 (Figs. 1 and 2). The largest regional contributions to global glacier mass loss are from Alaska (22%), the Canadian Arctic (20%), peripheral glaciers in Greenland (13%), and the Southern Andes (10%). Compared with regional glacier mass in 2000 (Table 1 and  Methods), the largest relative ice loss occurred in regions with a small glacier area (that is, ≤15,000 km^2^): Central Europe (−39%), Caucasus (−35%), New Zealand (−29%), Asia North (−23%), Western Canada and USA (−23%), and the low latitudes (−20%). The other regions, with a large glacier area (that is, >15,000 km^2^), lost between 2% and 12% of their ice. Specific mass changes in metre water equivalent (1 m w.e. = 1,000 kg m^−2^) represent the mass change averaged over the glacier surface and allow comparison of the intensity of mass change across regions. Regions with a small (large) glacier area typically feature specific mass-change rates more (less) negative than −0.5 m w.e. yr^−1^ (Table 1). Exceptions include Alaska, Arctic Canada south and the Southern Andes, which have a large glacier area not located at high latitude (or altitude) and strong mass-loss rates (Table 1). Comparison of regional change rates between the first (2000–2011) and the second (2012–2023) half of the record indicates an increased mass loss in 14 out of 19 regions; this increase is concurrent with the general pattern of atmospheric warming, overlaid by regional decadal climate variability^11,27,37^. As such, we note that the regions with an increase in mass loss include South Asia west, where slightly positive mass changes and glacier advances have been previously observed in several mountain ranges over the past decades^38^, and recent observations indicate a decline or ending of the so-called Karakoram-Kunlun Anomaly^27,39^. A slowdown of mass loss was found in Iceland and Scandinavia, which can be attributed to regional cooling and an increase in winter precipitation owing to persistent anomalies in large-scale atmospheric circulation^27,40,41^.

GlaMBIE developed the framework and methodology to compile and update mass-change estimates based on the main in situ and satellite observations. As such, we extended the observation period in all glacier regions from 2000 to 2023 and provided these data at annual resolution. This analysis offers an observational baseline for future studies to better understand the impact of climate change on glaciers and the associated downstream effects at the regional scale. Although the current annual resolution estimates are sufficient to assess the contribution of glaciers to sea-level rise, a monthly (or higher) temporal resolution is needed to quantify the contribution of glaciers to the hydrological cycle^6^.

## Differences among observation methods

**Extended Data Fig. 3:**
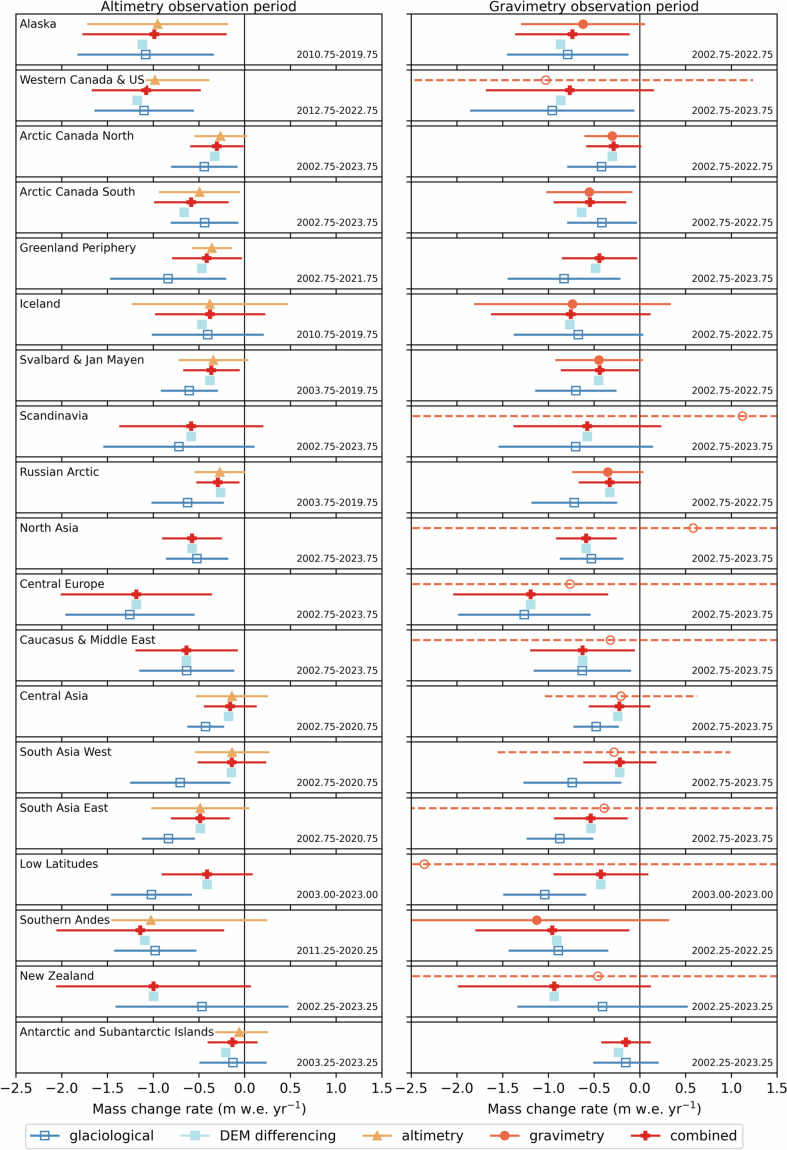
Regional mass-change rates per observation method. For each region (rows), mean specific mass-change rates (shown as markers) and interannual variability (one standard deviation, shown as lines) are compared among different methods over common observation periods for altimetry (left column) and gravimetry (right column). The common observation periods are shown as decimal years (bottom right). The gravimetry period does not include the hydrological year 2007/08. The combined estimates include only the observation methods indicated by complete markers and solid lines. Empty markers or dashed lines indicate results not considered for the combined estimates. Differences between observation methods and related uncertainties are given in Extended Data Table 2. Source Data

GlaMBIE is a comprehensive study that compares and quantifies differences in trend and variability of glacier mass changes among observation methods in all regions (Extended Data Fig. 3 and Extended Data Table 2). Combining interannual variability from glaciological observations with long-term trends from DEM differencing provided results for all 19 glacierized regions over the entire period 2000 to 2023. Altimetry provided results for 13 regions, generally covering periods after 2003 or 2010 related to data availability from ICESat^42,43^ and CryoSat-2^28^, respectively. The missing regions either contain small and widely scattered glaciers or have not been covered by the mission planning. Gravimetry covers the operation periods of the Gravity Recovery and Climate Experiment (GRACE, 2002–2017) and GRACE Follow-On (2018 to present)^30,31^ and provides results for all regions, excluding the peripheral glaciers of Greenland and Antarctica, owing to the high uncertainty of separating their mass-change signal from that of the ice sheets. For regions with a small glacier area (that is, Western Canada and USA, Scandinavia, North Asia, Central Europe, Caucasus, low latitudes, and New Zealand), we excluded gravimetric results from our combined estimates because of implausible trends and variabilities (Extended Data Fig. 3) owing to measurement noise and likely interference of proximal hydrological mass changes^30,31^. Similarly, we excluded gravimetric results from our combined estimates for Central Asia, South Asia east and South Asia west owing to possible leakage between these regions^30,31^, noting that aggregated results for the entire High Mountain Asia can be considered with higher confidence.

The results of the different observation methods are shown together with the combined estimates for all regions in Extended Data Fig. 3. Related differences are quantified over common observation periods in Extended Data Table 2. In general, the long-term trends and interannual variabilities of different observation methods agree within reported uncertainties. Compared with DEM differencing, available for all regions, altimetry observed less negative mass changes, on (arithmetic) average by 0.08 ± 0.08 m w.e. yr^−1^. The largest differences were found in Western Canada and USA, where altimetry results are less negative by 0.19 ± 0.12 m w.e. yr^−1^. In regions where our combined estimates include gravimetry, the latter generally agrees well with DEM differencing, with a mean difference of 0.02 ± 0.08 m w.e. yr^−1^. Larger differences are found for only Alaska and the Southern Andes, with differences of +0.24 ± 0.07 m w.e. yr^−1^ and –0.22 ± 0.25 m w.e. yr^−1^, respectively. For glaciological observations, our results confirm earlier studies^21,44,45^, which showed that the long-term trend from the small glaciological sample does not represent regional means. On average, among all regions, we found a mean bias of –0.10 ± 0.10 m w.e. yr^−1^, with values ranging between –0.61 ± 0.10 m w.e. yr^−1^ (low latitudes) and +0.53 ± 0.11 m w.e. yr^−1^ (New Zealand).

**Extended Data Fig. 4:**
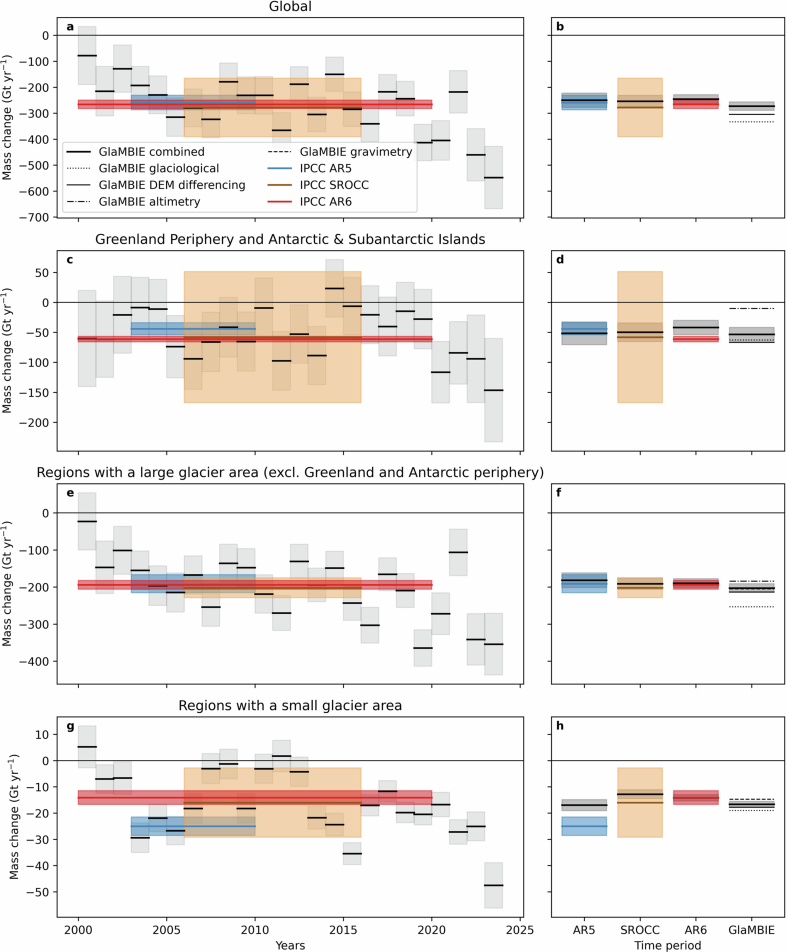
Glacier mass-change estimates in comparison with IPCC results. Comparison of our combined annual glacier mass changes (in Gt) with mean annual mass-change rates (in Gt yr^−1^) from past IPCC reports^15,16,47^ for regional aggregations at the global level (**a**), for Greenland and Antarctic periphery (**c**), regions with a large glacier area (**e**, excluding **c**), and regions with a small glacier area (**g**). The subplots on the right (**b**, **d**, **f**, **h**) show mean annual mass-change rates over the three IPCC assessment periods (AR5: 2003–2009, SROCC: 2006–2015, AR6: 2000–2019), and the GlaMBIE period (2000–2023). For the latter, we show our combined estimates (black line with grey error bars) together with the change rates from glaciological observations, DEM differencing, altimetry, and gravimetry, if available. We note that the long-term trends from glaciological observations and gravimetry in regions with a small glacier area were not used for the combined estimates. Uncertainties are shown for 95% confidence intervals. Source Data

Although the differences among observation methods are often within uncertainties (at 95% confidence intervals), they still pose a challenge when cumulating over longer time periods. As such, the global (area weighted) mean difference between DEM differencing and altimetry results in a cumulative difference of +2.27 ± 0.33 m w.e. over the entire 24-year period, which corresponds to about 1,602 ± 235 Gt or 4.4 ± 0.6 mm sea-level equivalent. The interannual variability generally agrees well among observation methods with differences smaller than 0.2 m w.e. yr^−1^ (Extended Data Fig. 3). An exception is the Southern Andes, where interannual variabilities from glaciological observations differ by 0.82 m w.e. yr^−1^ and 0.90 m w.e. yr^−1^ from altimetry and gravimetry, respectively. For regions with a large glacier area, the relative differences to the combined estimate in total mass-change rate (−203 ± 11 Gt yr^−1^) are −25% for glaciological, −5% for DEM differencing, +9% for altimetry and −1% for gravimetry (Extended Data Fig. 4b).

With the quantification of differences among observation methods, GlaMBIE provides an opportunity to better understand and reduce the discrepancies among observation methods. As an example, a detailed comparison^28^ of altimetry and DEM differencing showed that differences in observed change rates do not necessarily stem from differences in the spatial coverage of given methods (for example, in ice margins and areas of poor radiometric contrast) but exist across the entire glacier hypsometry. In addition, GlaMBIE shows the need for homogenization of uncertainty assessments^32^ and improved estimates of volume-to-mass conversion, especially over short survey periods^29^. To further develop the applied observation methods, an intercomparison at higher spatial and temporal resolution is needed. As such, mass-change estimates at glacier (or higher) spatial resolution are required to identify the sources of observational differences^26,46^. Validation of regional mass changes remains a challenge, and future efforts should be directed towards using high-quality, independent observations at multiple local sites to quantify uncertainties better and propagate these to the regional scale. For example, reference data at the glacier scale, such as from airborne laser surveys, are less prone to errors than spaceborne observations and can be used for validation.

## Comparison with IPCC estimates and outlook

Our results generally confirm glacier mass-loss trends reported by the latest IPCC reports. Over common periods, our combined estimates of global glacier mass changes are less negative (but within uncertainties) by 4%, 9% and 8% than estimates in the IPCC’s Fifth Assessment Report (AR5)^47^, Special Report on the Ocean and Cryosphere in a Changing Climate (SROCC)^15^ and AR6^16^, respectively (Extended Data Table 3). At a regional level, the differences to the results from AR6 range between −20% for the Southern Andes and +53% for the Antarctic and subantarctic islands but are often within uncertainties (Extended Data Table 3). Even if these differences are not always significant at the 95% confidence level, they are in many cases large enough to be worth considering given the severe impacts of glacier changes. The values reported by IPCC are based on a few selected studies per region. In contrast, our GlaMBIE protocols (Extended Data Fig. 1) consistently combine time series from the main observation methods, each based on input from multiple research teams. As such, our coordinated community effort yields a most comprehensive estimate of glacier mass changes from observations. The annual resolution of the GlaMBIE estimates represents an important improvement over the multi-annual change rates reported by the IPCC. Over aggregated regions (Extended Data Fig. 4), we found the most prominent differences for Greenland periphery, and for Antarctic and subantarctic islands, where our estimate (−53 ± 11 Gt yr^−1^) is less negative than reported in IPCC AR6 (−61 ± 4 Gt yr^−1^) owing to our inclusion of results from altimetry; and in regions with a small glacier area, where our estimate (−17 ± 1 Gt yr^−1^) is also less negative than that reported in IPCC AR5 (−25 ± 4 Gt yr^−1^) owing to our exclusion of results from gravimetry. The global trend reported in IPCC AR6 (2000–2019: −267 ± 16 Gt yr^−1^) is based on, and identical to, the results of a global DEM differencing study^27^, with minor regional differences due to averaging with selected other studies^16^. Hence, our global estimates are also 8% less negative than the results from Hugonnet et al.^27^ but provide interannual variability, which is not well captured by DEM differencing. GlaMBIE extends the temporal coverage to 2023, provides results at annual resolution and has established a community mechanism to allow estimates to be updated towards the IPCC Seventh Assessment Report. Regarding uncertainties, our estimates come with error bars that are smaller than the ones reported in IPCC SROCC (Extended Data Table 3 and Extended Data Fig. 4), which can be explained by the improvement in observational coverage. Compared with IPCC AR5 and AR6, our estimates feature similar uncertainties over multiyear periods but additionally have the advantage of providing results and uncertainties at annual resolution.

**Extended Data Fig. 5:**
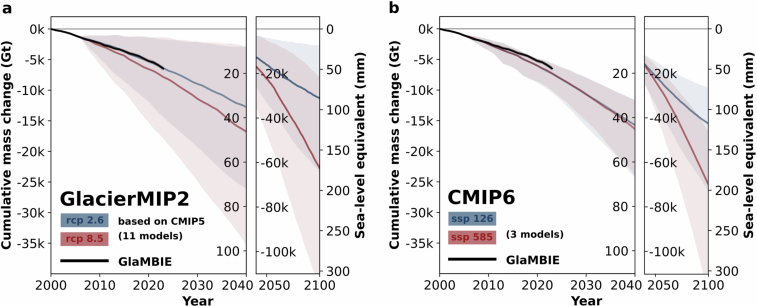
Glacier mass-change observations and model projections. Comparison of observed cumulative glacier mass changes (in Gt, left y-axis) and corresponding cumulative sea-level equivalents (in mm, right y-axis) since 2000 with ensemble projections for 2007–2040 and 2040–2100 from the glacier model intercomparison project (GlacierMIP2, based on CMIP5)^12^, as used in IPCC AR6^16^ (**a**) and from a more recent model study (based on CMIP6)^18^ (**b**). Glacier mass-change observations (black line) are accompanied by their 95% confidence intervals (grey shading). For the projections, ensemble medians (blue and red lines) are shown with 90 percentile ranges (blue and red shadings) for low and high emission scenarios, respectively. Projections have been offset at their start date (2007) to fit the cumulative value of the observations. Regional comparisons are shown in Supplementary Information Figs. 20 and 21. Source Data

Glacier model ensembles, as presented in IPCC AR6^12,16^, projected a (full) ensemble median mass loss of about 40 mm sea-level equivalent by 2040 (relative to 2000; Extended Data Fig. 5a) that ranges from 8 mm to 97 mm (95 percentile range). Relative to 2000, our global mass-change assessment estimates 18.1 ± 0.9 mm sea-level equivalent by 2023, thus agreeing with the median of modelled low-emission scenarios. Considering already committed mass loss owing to the delay in glacier response to climate change^48,49^, we can expect glacier mass loss to continue in the coming decades, regardless of emission pathways. This indicates that we have already passed the IPCC AR6 lowest mass-loss projections over the period from 2000 to 2040. In most regions, observations follow the (full) ensemble median within the 68-percentile range (Supplementary Fig. 20). Although glacier projections were more negative than observations in the Russian Arctic, Central Asia and South Asia west, projections were substantially less negative than observations in the Southern Andes and New Zealand, which calls for further investigation. Large deviations between model ensemble and observations were already identified in these regions by a recent study involving one glacier model^50^. They were attributed to the lack of calibration data (Russian Arctic), a negative bias in glaciological observations used for model calibration (Central Asia and South Asia west), issues in model set-up forced with reanalysis data (Southern Andes), and large portions of marine-terminating glaciers not well represented in the model (Southern Andes).

Recent model intercomparison efforts^12^ indicate that the large uncertainty in projected glacier evolution is driven by differences in both glacier models and the data used for initial conditions and calibration. In contrast, uncertainty in the emission scenario becomes dominant only towards the end of the twenty-first century. The model ensemble presented in IPCC AR6^12,16^ was mainly calibrated to glaciological observations, for which we showed limitations concerning sample size and a generally negative bias. More recent modelling studies^11,18^ are calibrated using glacier-specific results from DEM differencing^27^ with global coverage. Those results reveal a cumulative projected mass loss between 32 mm and 67 mm sea-level equivalent by 2040 (relative to 2000, 95 percentile range; Extended Data Fig. 5b). This confirms the indication from observation that the lowest mass-loss projections by 2040 provided within IPCC AR6 have already been exceeded. Regionally (Supplementary Fig. 21), more recent model projections are better aligned to observations owing to the calibration to DEM differencing with global coverage. Substantial deviations remain in the Antarctic and subantarctic islands, where observations show significantly less mass loss than model projections. The difference in this region, with a large proportion of marine-terminating glaciers^20^, could arise from the fraction of glacier ice below sea level (not contributing to sea-level rise), which is included in model projections but excluded in our observational estimates.

Glacier mass loss in the second half of this century strongly depends on emission scenarios. By 2100 (relative to 2015), about one-quarter (25–29%, low-emission-scenario range) to one-half (43–54%, high-emission-scenario range) of the global glacier mass is projected to be lost under Coupled Model Intercomparison Project Phase 6 scenarios^18^ (Extended Data Fig. 5). Altogether, our observations and recent modelling studies^11,18^ indicate higher projected glacier mass loss than the estimates from IPCC AR6^12,16^. We are, therefore, facing continued and possibly accelerated mass loss until the end of this century. This underpins the IPCC’s call^16^ for urgent and concrete actions to reduce greenhouse gas emissions and associated warming to limit the impact of glacier wastage on local geohazards, regional freshwater availability and global sea-level rise.

## Methods

GlaMBIE (https://glambie.org) is a community effort to compile, homogenize, combine and analyse regional estimates of glacier mass changes from four distinct observation methods, or hybrids of observation methods: glaciological observations, DEM differencing, altimetry and gravimetry (Extended Data Fig. 1). In total, we analysed 233 regional estimates of glacier mass changes from about 450 data contributors organized in 35 research teams (Supplementary Tables 1 and 2). Data contributions were compiled through an open call for data submission and selected based on expert evaluation of their confidence levels. Within each observation method, the selected input data were homogenized for time, space and unit domains using common corrections. They were then combined first within and second among methods for each of the 19 regions and finally aggregated to global estimates. Below, we briefly summarize the input data (Extended Data Fig. 1) and the general workflow (Extended Data Fig. 2) of our intercomparison exercise, including key equations. The full methodological details are available in our code (‘Code availability’). For more information on the different observation methods, we refer to a recent review on measuring glacier mass changes from space^26^ and the methods references of our input data as listed in Supplementary Table 1. As an output, GlaMBIE provides the native input data in a standardized format, combined estimates per observation method and combined estimates among observation methods (‘Data availability’). Given the available data, we consider our combined estimate (among methods) to best reflect the expert evaluation of the GlaMBIE community.

### Glacier regions

We used the 19 first-order glacier regions defined by the Global Terrestrial Network for Glaciers^51^. These regions appear suitable for glacier studies owing to their manageable number and geographical extent, which in most cases is close to the spatial correlation distance of the variability of glacier mass change, which is several hundred kilometres^25,52^. In our analysis, we differentiate between regions with a large glacier area (>15,000 km^2^) and regions with a small glacier area (<15,000 km^2^).

### Glacier area, volume and mass in 2000

We aggregated the regional glacier area from the Randolph Glacier Inventory (RGI 6.0)^19,53^. This snapshot inventory provides one digital outline and a corresponding area for each glacier in the world. Although RGI aims for a reference year in 2000, the regional (area-weighted average) reference years deviate by up to 22 years (Extended Data Table 4). To account for glacier shrinkage, we used regional glacier area-change rates (percent per year) compiled from IPCC AR5^47^ (Ch. 4, Fig. 4.10 and Table 4.SM1), extended with additional literature from Zemp et al.^25^. With these annual area-change rates, we corrected glacier area from RGI 6.0 to the year 2000 (Table 1) and computed yearly time series (*t*_*y*_) of regional glacier area (*S* in km^2^):

$${S}_{{t}_{y}}={S}_{{t}_{0}}+({t}_{y}-{t}_{0})\times \delta S/\delta t\times {S}_{{t}_{0}}\,,$$

where ${S}_{{t}_{0}}$ is the regional glacier area in the (area-weighted average) reference year *t*_0_ and *δS*/*δt* is the annual area-change rate (in percentage). It is noted that the latest version of RGI (7.0)^54,55^ was not used as it only became available after the launch of the GlaMBIE, and its full implementation in glacier mass-change assessments will take a few years. Regional glacier volume and mass are from a multi-model consensus ice-thickness estimate^20^, which was based on glacier outlines from RGI 6.0. We used their estimates relevant to sea-level rise, that is, subtracting the ice fraction below present-day sea level. In addition, we corrected their values to the year 2000 (Table 1) by using annual mass changes after 2000 from our combined estimate (see below) and before 2000 from the input dataset of Dussaillant et al.^56^, which provides mass-change estimates from 1976, combining glaciological observations with DEM differencing. For regional glacier area and related changes over time, we assume a general uncertainty of ±5% based on a glacier mapping intercomparison study^57^. Uncertainties for glacier volume and mass are from the multi-model consensus ice-thickness estimate^20^.

### Glaciological observations

This approach determines glacier mass changes traditionally in the unit metre water equivalent (1 m w.e. = 1,000 kg m^−2^) from in situ observations of accumulation and ablation, generally based on measurements at stakes and in snow pits^58^. The method provides surface mass changes from a few hundred glaciers distributed in almost all glacier regions over seasonal-to-annual timescales with some records beginning in the late-nineteenth century^21,24^. We analysed glaciological observations from the World Glacier Monitoring Service^24,59^. The data cover the period from 2000 to 2023 and are available for all but two glacier regions (Extended Data Fig. 1 and Supplementary Table 1). We replaced the data gaps in Arctic Canada south with observations from Arctic Canada north and the gaps in the Russian Arctic with observations from Svalbard. We only used the interannual variability from glaciological observations, which is considered high confidence^25,52^ owing to its spatial correlation over several hundred kilometres^60,61^. The long-term trend, however, was not used owing to the sparse spatial coverage (typically well below 10%) and limited representativeness of the glaciological samples concerning total mass changes^44,62–64^. Also, the glaciological method does not account for ice discharge of marine-terminating glaciers, which is a relevant mass-loss component in some regions^34^ and implicitly accounted for in DEM differencing, altimetry and gravimetry. Consequently, we combined the temporal variability from the glaciological observations with long-term trends from DEM differencing (Extended Data Fig. 2).

### DEM differencing

This approach determines glacier elevation change (traditionally in the unit of metres) by repeated mapping of glacier surface elevations, such as from optical stereo photogrammetry or synthetic aperture radar interferometry^26,46^. The method provides multi-annual elevation differences, ideally corrected for vertical land motion^65^, and requires density assumptions for converting to geodetic mass changes^29^. DEM differencing represents glacier mass changes above sea level as it implicitly accounts for calving owing to ice discharge (contributing to sea-level rise), but it does not include any mass changes below water level (not contributing to sea-level rise) owing to the retreat or advance of lake- and ocean-terminating glaciers^34^. We used 42 geodetic estimates from DEM differencing from 12 research teams covering all glacier regions and the entire period since 2000 using various methods^27,46,66–78^ (Extended Data Fig. 1 and Supplementary Table 1). The regional assessments used various optical (for example, Advanced Spaceborne Thermal Emission and Reflection Radiometer (ASTER), GeoEye, Pléiades, SPOT-5/6/7 (SPOT from French ‘Satellite pour l’Observation de la Terre’), Wordview-1/2) or radar (for example, Shuttle Radar Topography Mission (SRTM), TerraSAR-X add-on for Digital Elevation Measurements (TanDEM-X)) sensors and products. The spatial coverage of regional results from participants ranged between 25% and close to 100%. The observational coverage was considered to be representative of the entire region or data gaps were filled by spatial or hypsometric interpolation^46^. DEM differencing often provides long-term regional trends at high confidence levels, but it does not fully represent seasonal or annual variability. Consequently, we combined the long-term trends from DEM differencing and the annual variability from glaciological observations.

### Altimetry

Laser and radar altimetry determine elevation change (traditionally in the unit of metres) along ground tracks or in swath modes, which must be extrapolated to glacier-wide results and aggregated to regional estimates. Elevation change can be derived at seasonal to monthly resolution, often reported as multi-annual change rates, from laser altimetry (2003–2009 from ICESat, since 2018 from ICESat-2) and radar altimetry (since 2010 from CryoSat-2, earlier from ERS-1/2 and Envisat). Results are partly corrected for elastic uplift rates from present-day and long-term ice-mass changes (for example, in Greenland periphery, Iceland)^65^ and require density assumptions for converting to geodetic mass changes^29^. Similar to DEM differencing, altimetry represents glacier mass changes relevant to sea-level rise. We analysed 41 geodetic assessments from altimetry from nine research teams covering 13 out of 19 regions using various methods^28,42,43,45,79–85^ (Extended Data Fig. 1 and Supplementary Table 1). Altimetry provides both temporal variability and long-term trends at high confidence levels for regions with a large glacier area, including the Greenland and Antarctic periphery. The missing regions either have small and widely scattered glaciers or have not been covered by the mission planning. Like DEM differencing, the spatial coverage differed between regions and sensors used. It was either considered representative for the entire region or data gaps were filled by spatial or hypsometric interpolation^28,86–88^.

### Gravimetry

This approach estimates mass-change anomalies traditionally in the unit of gigatonnes (1 Gt = 10^12^ kg) by measuring changes in the distance between two satellites in a shared orbit and by applying a series of corrections, for example, for atmospheric drag, solar radiation pressure, glacial isostatic adjustment, signal leakage and non-glacier hydrological components^26,30,31,89^. The method has almost continuously provided regional mass changes at monthly resolution since 2002, with a few dozen months of missing data (typically interpolated from the months before and after) and an observational gap of 11 months between the GRACE (2002–2017) and GRACE Follow-On (2018 to present) missions. For gravimetry, we analysed 78 data contributions from 7 research teams, covering 17 out of 19 regions using various methods^31,90–97^ (Extended Data Fig. 1 and Supplementary Table 1). Gravimetry provides both temporal variability and long-term trends at medium to high confidence levels for seven regions with a large glacier area and related large mass change. The periphery of Greenland and Antarctica is excluded owing to the high uncertainty of separating the mass-change signal of the glaciers and the ice sheets. Gravimetry estimates for regions with a small glacier area and related small mass changes are considered to be of low to no confidence owing to the leakage of non-glacier mass changes, limitations in the hydrological models and poor signal-to-noise ratio and, hence, are shown in the results (Extended Data Table 2 and Extended Data Figs. 1, 3 and 4) for completeness but not included in our combined estimates. The challenges of isolating the glacier signal with GRACE and GRACE Follow-On in regions with a small glacier area are well reflected in implausible mean regional change rates or interannual variabilities shown in Extended Data Fig. 3.

### Hybrid estimates

Some research teams provided estimates combining results from different observation methods, labelled ‘hybrid results’ here to distinguish them from the ‘combined results’ derived by the GlaMBIE workflow. We analysed 58 hybrid results from 7 research teams covering all 19 regions (Extended Data Fig. 1 and Supplementary Table 1). These hybrid estimates are diverse in their approaches. Dussaillant et al.^56,98^ and Huss et al.^23^ combined glaciological observations with geodetic estimates at the glacier scale for all 19 regions with similar but partly different approaches. From their results, we assigned the temporal variability to the glaciological and the long-term trends to the DEM differencing methods. Box et al.^99^ similarly calibrated glaciological observations to results from gravimetry in Alaska, the Canadian Arctic, Iceland, Svalbard and the Russian Arctic. From their results, we assigned the temporal variability to the glaciological and the long-term trends to the gravimetric method. Colgan et al.^100^ inverted low-resolution gravimetry changes over Greenland periphery and the Canadian Arctic using high-resolution altimetry observations. We used the results for the Canadian Arctic and assigned their long-term trends to gravimetry. Ke et al.^101^ calculated long-term trends from ICESat-2 with reference to SRTM and National Aeronautics and Space Administration’s Digital Elevation Model (NASADEM) over High Mountain Asia. We assigned their results to altimetry. Pálsson et al.^102^ submitted two versions of glaciological observations covering 90% of the glacier area over Iceland, one with and one without corrections for non-surface mass-change components. We used their results with corrections and assigned both their trend and variability to the glaciological method. Miles et al.^103^ estimated glacier mass changes over High Mountain Asia in an approach combining DEM differencing, ice velocities and glacier thickness estimates. We excluded their results to avoid double counting the long-term trends from DEM differencing by Brun et al.^69^, submitted to GlaMBIE separately.

### The GlaMBIE workflow

The principal approach and workflow of the intercomparison exercise are illustrated in Extended Data Fig. 2 and described in the following paragraphs. In summary, we compiled glacier mass changes through an open call to the research community from the different observation methods at their native temporal resolution and in their traditional units for the 19 predefined regions. After primary quality control, input data were homogenized for time, space and unit domains and were selected based on an expert evaluation. Selected datasets were de-trended according to their annual variability and long-term trends. After re-trending, datasets were combined within and among observation methods.

### Quality control, homogenization and selection

All data submissions were run through basic quality controls, including checks for completeness and correctness concerning data format, the plausibility of value ranges relating to units, potential outlier detection, and identification of spatial and temporal data gaps. A data-quality report with plots for visual inspection was generated for each data submission, and identified issues were discussed and resolved with the data provider. In the first processing step, all input data were homogenized concerning unit, temporal and spatial domains to reduce corresponding biases and to make results comparable across observational sources. Units were converted—if required—to specific mass changes (in metre water equivalent), considering time-variable glacier area as outlined above. Results from gravimetry (in gigatonnes) were divided by the regional glacier area, considering area changes over time. Results from altimetry and DEM differencing (in metres) were converted assuming an average density of the volume change of 850 kg m^−3^, assuming no change in bulk glacier density over the observation period. In line with the work by Huss^29^, we prescribed the related uncertainty to be ±60 kg m^−3^ and chose to increase it to ±120 kg m^−3^, ±240 kg m^−3^ and ±480 kg m^−3^ for survey periods shorter than 10 years, 5 years and 1 year, respectively. We aligned the temporal domain to annual resolution following hydrological years in the regions of the Northern Hemisphere (1 October to 30 September), in the tropics (1 January to 31 December) and in the Southern Hemisphere (1 April to 31 March). Input data with monthly or seasonal resolution were aggregated to annual sums over the hydrological year. Input data with multi-annual resolutions, such as from DEM differencing or partly from altimetry and gravimetry, were used for long-term trends only, corrected to hydrological years (if needed) by assuming a linear change. Regional results were corrected from hydrological to calendar years for global aggregation using regional glaciological time series, which were downscaled from seasonal to monthly resolution using an analytical model approach^104^. The uncertainty of this temporal correction was assumed to be ±10% of the correction, which depends on the seasonal mass turnover of the region^105^. The spatial domain was regularized by using common glacier regions and (earlier) converting all results to specific mass changes (in metre water equivalent) under consideration of regional area changes. This approach allowed us to use common regional glacier area and area-change rates to calculate regional mass changes in gigatonnes across all input data.

After quality control and homogenization, all input data underwent an expert evaluation by the GlaMBIE community (that is, co-authors and data contributors). In a workshop, the consortium and representatives from all data contributors assessed the confidence levels^106^ (no, medium or high) of both temporal variability and long-term trend of each observation method at a regional level. On the basis of this consensus decision, we excluded input data from observation methods regionally evaluated as having ‘no confidence’. We used input data of medium or high confidence with the same weight. Our combined regional estimates give equal weight to all selected input data within and among observation methods (that is, glaciological and DEM differences, altimetry, gravimetry). Within observation methods, this approach implicitly gives a larger weight to multiple results from the same sensors, that is, various results based on ASTER within DEM differencing, or numerous results based on the same GRACE or GRACE Follow-On gravity field solution within gravimetry. Among observation methods, the weight of a given method depends on the availability of regional data and the assessed confidence level. As an example, the glaciological observations have no weight on regional trends (owing to ‘no confidence’) but determine the temporal variability by one-third in Iceland (sharing the same weight with altimetry and gravimetry) and fully in Central Europe (owing to lack of confident results from the other methods). Altogether, our combined estimates reflect the currently available observations with a potential bias towards specific sensors and approaches. An overview of all included and excluded datasets is given in Extended Data Fig. 1 and Supplementary Figs. 1–19, and the regional weight of each observation method can be derived from Extended Data Fig. 3.

### Separation of temporal variability from the long-term trend

We note that in the case of all time series covering the same observation period, one could simply average the annual values. For overlapping observation periods, however, simple averaging would introduce artefacts, that is, jumps from average values within the common observation period to observed values of one series outside the common observation period. In our approach, we first separate the annual anomalies from the period average and then calibrate each series with reference to a common period of records (cPoR)^25,52^ (Extended Data Fig. 2a), which differs depending on region and method. We calculated for each time series (*i*) the annual anomaly *β* for a given year (*y*) as the difference between the observed mass change *B* and the arithmetic mean balance $\bar{B}$ over the common period of records:

$${\beta }_{i,y}={B}_{i,y}-{\bar{B}}_{i,{\rm{c}}{\rm{P}}{\rm{o}}{\rm{R}}}\,.$$

The resulting time series of annual anomalies were then averaged to one time series of mean yearly anomalies ${\bar{\beta }}_{y}$. This yearly time series was then re-trended by adding the long-term trend of each input dataset:

$${B}_{{\rm{c}}{\rm{a}}{\rm{l}},i,y}={\bar{\beta }}_{y}+{\bar{B}}_{i}\,.$$

This resulted in multiple time series of calibrated annual mass changes ${B}_{{\rm{cal}},i}$ (one for each input dataset), which have different long-term trends (based on the input dataset) but a common estimate of annual anomaly. In cases without annual observations (for example, altimetry pre-2010 or gravimetry during the observational gap between GRACE and GRACE Follow-On), we used the averaged anomaly from the other observation methods to fill in ${\bar{\beta }}_{y}$ for missing years. Finally, these calibrated yearly time series were averaged to get one time series ${\bar{B}}_{{\rm{cal}}}$ for each observation method and region.

The uncertainty of the mean annual anomaly ${\sigma }_{\bar{\beta }}$ combines the reported observational uncertainty *σ*_obs_ of the individual input datasets (*i*) with the variability of the ensemble *σ*_var_, taken as independent:

$${\sigma }_{\bar{\beta }}=\sqrt{{\bar{\sigma }}_{{\rm{obs}}}^{2}+{\sigma }_{\mathrm{var}}^{2}}\,,$$

with

$${\bar{\sigma }}_{{\rm{obs}}}=\frac{1}{N}\sqrt{{\sum }_{i=1}^{N}{\sigma }_{{\rm{obs}},i}^{2}}\,,$$

and

$${\sigma }_{{\rm{var}}}=\frac{{\rm{s.d.}}({\beta }_{i,y{\rm{PoR}}})}{\sqrt{{N}_{y}}}\,.$$

Thereby, the ensemble variability was expressed as standard error, which was calculated from the standard deviation (s.d.) of the annual values from the common period with full sample coverage (PoR) divided by the number of time series (*N*) for a given year (*y*).

The uncertainty of the calibrated time series was calculated by combining the uncertainties of the mean anomalies ${\bar{\sigma }}_{{\bar{\beta }}_{y}}$ and the long-term trends ${\sigma }_{\bar{{B}_{i}}}$ as:

$${\sigma }_{{B}_{{\rm{cal}},i,y}}=\sqrt{{\bar{\sigma }}_{\bar{{\beta }_{y}}}^{2}+{\sigma }_{\bar{{{\rm{B}}}_{i}}}^{2}}\,.$$

The uncertainty of the mean calibrated time series ${\sigma }_{{\bar{B}}_{{\rm{c}}{\rm{a}}{\rm{l}}}}$ combines (again) the uncertainties of the individual calibrated time series with the variability of the corresponding ensemble.

### Combined estimate within observation methods

The approach of de-trending and re-trending was applied to combine the input data for each observation method. We combined the glaciological method’s temporal variability with the long-term trends from geodetic DEM differencing. We separately combined temporal variability and long-term trends from within the methods for altimetry and gravimetry. The expert evaluation assigned data submissions from hybrid approaches to the best-fitting method. As a result, for each region, our approach provided one combined estimate for (1) glaciological observations and DEM differencing, (2) altimetry and (3) gravimetry (Extended Data Fig. 2b), provided that corresponding data had been submitted. The uncertainties of the combined estimates were calculated, as explained in the section above.

### Combined estimate among observation methods

On the basis of these (up to three) combined estimates per region, we then calculated a combined estimate among observation methods using the same approach as before. This means that we de-trended the time series with reference to the common observation period. Then, we averaged these anomalies and re-trended the time series of mean anomalies to the trends of the observation methods over the common period of records. Finally, we averaged the resulting time series to get a combined regional estimate among observation methods (Extended Data Fig. 2b). The regional results are provided as mean specific mass changes (*B*_*reg*_ in m w.e.) and as mass changes (Δ*M*_*reg*_ in Gt). The latter was calculated as the product of the specific mass changes and the regional glacier areas (*S*_*reg*_), considering area changes (see above):

$${\Delta M}_{reg}={B}_{reg}\times {S}_{reg}\,.$$

The corresponding uncertainty (*σ*_Δ*M*_) was calculated by combining fractional uncertainties related to the combined observations (*σ*_*B*_) and regional glacier area (*σ*_*S*_):

$${\sigma }_{{\Delta M}_{reg}}=|{\Delta M}_{reg}|\times \sqrt{{\left(\frac{{{\sigma }_{B}}_{reg}}{{B}_{reg}}\right)}^{2}+\,{\left(\frac{{{\sigma }_{S}}_{reg}}{{S}_{reg}}\right)}^{2}}\,.$$

### Global aggregation

Global estimates were computed by aggregation of regional results, that is, by calculating regional area-weighted means for specific mass changes (*B*_glob_ in m w.e.), considering area changes (see above):

$${B}_{{\rm{g}}{\rm{l}}{\rm{o}}{\rm{b}}}=\frac{{\sum }_{reg=1}^{19}{B}_{reg}\times {S}_{reg}}{{S}_{{\rm{g}}{\rm{l}}{\rm{o}}{\rm{b}}}}\,,$$

and by simple sums for global mass changes (Δ*M*_glob_ in Gt):

$${\Delta M}_{{\rm{g}}{\rm{l}}{\rm{o}}{\rm{b}}}=\,\mathop{\sum }\limits_{reg=1}^{19}{\Delta M}_{reg}\,.$$

Related uncertainties were calculated, assuming that regional estimates are independent, as:

$${\sigma }_{{B}_{{\rm{g}}{\rm{l}}{\rm{o}}{\rm{b}}}}=\frac{\sqrt{{\sum }_{reg=1}^{19}{({\sigma }_{{B}_{reg}}\times {S}_{reg})}^{2}}}{{S}_{{\rm{g}}{\rm{l}}{\rm{o}}{\rm{b}}}}\,,$$

and

$${\sigma }_{{\Delta M}_{{\rm{g}}{\rm{l}}{\rm{o}}{\rm{b}}}}=\,\sqrt{{\sum }_{reg=1}^{19}{({\sigma }_{{\Delta M}_{reg}})}^{2}}\,.$$

Observations from all methods represent glacier mass changes above sea level, or—to be more precise—above floatation level. Hence, the conversion to sea-level equivalents was directly calculated by dividing the global mass change (Gt) by an ocean area of 362.5 million square kilometres^107,108^. These estimates include glacier mass changes in hydrologically landlocked (endorheic) basins, which only indirectly contribute to sea-level changes^109^. We note that the uncertainties (*σ*) above are formulated at the 1*σ* level (that is, 68% confidence interval) to simplify equations, whereas the results in the main text, figures and tables are reported at the 1.96*σ* level (that is, 95% confidence interval).

## Online content

Any methods, additional references, Nature Portfolio reporting summaries, source data, extended data, supplementary information, acknowledgements, peer review information; details of author contributions and competing interests; and statements of data and code availability are available at 10.1038/s41586-024-08545-z.

## Supplementary information

Supplementary Information

Peer Review File

## Source data

Source Data Fig. 1

Source Data Fig. 2

Source Data Extended Data Fig. 1

Source Data Extended Data Fig. 3

Source Data Extended Data Fig. 4

Source Data Extended Data Fig. 5

## Figures and Tables

**Extended Data Table 1:**
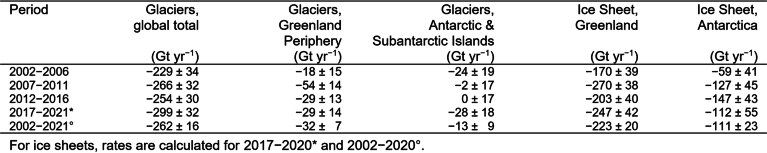
Comparison of mass changes from glaciers and ice sheets

To compare mass changes for peripheral glaciers (from this study) and for ice sheets (from IMBIE^17^) in Greenland and Antarctica, we calculated mean annual change rates (Gt yr^−1^) for five-year periods from calendar years 2002 to 2021. Note that we corrected ice sheet estimates by −10 Gt yr^−1^ and −3 Gt yr^−1^ for the Greenland and Antarctica ice sheets, respectively, to account for the leaking effects of glacier signal into ice sheet estimates from gravimetry^17^. Uncertainties of glacier mass changes correspond to 95% confidence intervals, while ice sheet uncertainties are reported as one standard error^17^.

**Extended Data Table 2:**
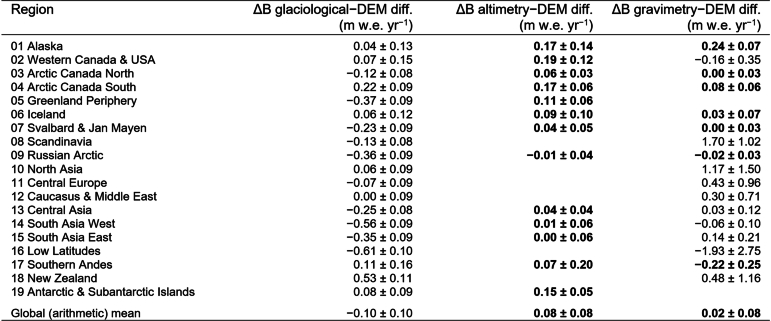
Regional and global differences between observation methods

Differences in specific glacier mass-change rates (ΔB, in m w.e. yr^−1^) from glaciological observations, altimetry, and gravimetry compared to DEM differencing. The differences are calculated from mean annual change rates over common survey periods (Extended Data Fig. 3) and shown with combined uncertainties (95% confidence interval). Values in bold indicate that the corresponding results were used to calculate our combined mass-change estimates. Over all regions, glaciological results are 0.10 m w.e. yr^−1^ more negative than DEM differences, while altimetry and gravimetry are less negative by 0.08 and 0.02 m w.e. yr^−1^, respectively.

**Extended Data Table 3:**
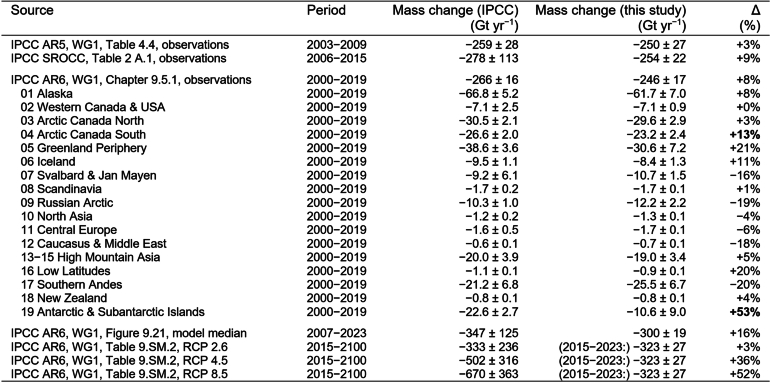
Glacier mass-change rates compared to IPCC results

Comparison of mean annual mass-change rates (in Gt yr^−1^) from past IPCC reports^15,16,47^ with our combined estimates over common survey periods, with relative differences (Δ) given as percentages and shown in bold when significant. The table compares global estimates for the last three IPCC reports and regional estimates for IPCC AR6. For the different emission scenarios from AR6, we compare our combined observational estimate with the model median over the common period from 2007 to 2023. In addition, we compare our observational estimate from 2015 to 2023 with projected mass-change rates from 2015 to 2100 to put the observed annual change rates into the context of future emission scenarios. Uncertainties are given at 95% confidence intervals.

**Extended Data Table 4:**
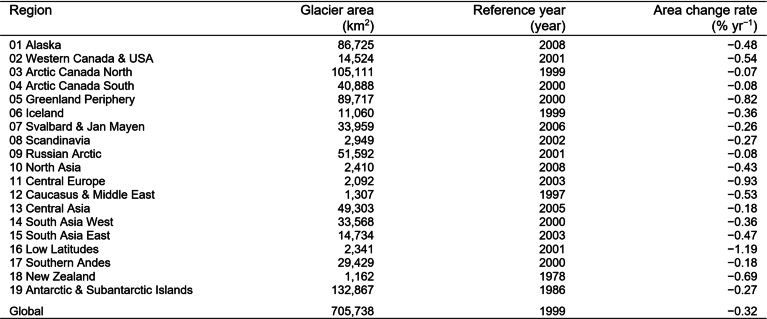
Regional glacier area and area-change rates

Regional and global glacier area and related (area weighted) reference years from Randolph Glacier Inventory (version 6.0)^19^, and (area weighted) mean annual change rates (percentage per year) from a compilation of (sub)regional glacier area-change assessments^14,25^.

## Data Availability

Regional glacier mass-change estimates from the individual research teams and combined results within and among observation methods are available from the World Glacier Monitoring Service (10.5904/wgms-glambie-2024-07). Figure 1 and Extended Data Fig. 2 used global background maps from Natural Earth^110^ and glacier regions defined by the Global Terrestrial Network for Glaciers^51^. Source data are provided with this paper.

## Code availability

The code for implementing our approach is available on GitHub (https://github.com/earthwave/glambie) and on Zenodo at 10.5281/zenodo.14186227 (ref. ^111^).
