# Supplementary material for: Community estimate of global glacier mass changes from 2000 to 2023
Source: Nature. 2025 Feb 19;639(8054):382–8. doi: 10.1038/s41586-024-08545-z (PMC11903323; doi:10.1038/s41586-024-08545-z)
Supplement: Supplementary file 1 — Supplementary Information [file 41586_2024_8545_MOESM1_ESM.pdf]

nature portfolio

https://doi.org/10.1038/s41586-024-08545-z

Supplementary information

Community estimate of global glacier mass changes from 2000 to 2023

In the format provided by the authors and unedited

Supplementary Information

Community estimate of global glacier mass changes from 2000 to 2023

The GlaMBIE Team

Correspondence to: michael.zemp@geo.uzh.ch

This document includes:

SI Table 1 | Data submissions to GlaMBIE

SI Table 2 | Research teams and data contributors to GlaMBIE

Description of SI Figures 1−19

SI Figure 1 | Data submissions and results for Alaska

SI Figure 2 | Data submissions and results for Western Canada & USA

SI Figure 3 | Data submissions and results for Arctic Canada North

SI Figure 4 | Data submissions and results for Arctic Canada South

SI Figure 5 | Data submissions and results for Greenland Periphery

SI Figure 6 | Data submissions and results for Iceland

SI Figure 7 | Data submissions and results for Svalbard & Jan Mayen

SI Figure 8 | Data submissions and results for Scandinavia

SI Figure 9 | Data submissions and results for Russian Arctic

SI Figure 10 | Data submissions and results for North Asia

SI Figure 11 | Data submissions and results for Central Europe

SI Figure 12 | Data submissions and results for Caucasus & Middle East

SI Figure 13 | Data submissions and results for Central Asia

SI Figure 14 | Data submissions and results for South Asia West

SI Figure 15 | Data submissions and results for South Asia East

SI Figure 16 | Data submissions and results for Low Latitudes

SI Figure 17 | Data submissions and results for Southern Andes

SI Figure 18 | Data submissions and results for New Zealand

SI Figure 19 | Data submissions and results for Antarctic & Subantarctic Islands

SI Figure 20 | Glacier mass-change observations and model projections (CMIP-5)

SI Figure 21 | Glacier mass-change observations and model projections (CMIP-6)

SI Figure 22 | Author contributions following the CRediT system

SI References

SI Table 1 | Data submissions to GlaMBIE. The submissions are organized by observation method and research team in alphabetical order and provide an overview of sensors or data products used, references to related publications on methods, and regions for which glacier mass changes were calculated. A complete list of data contributors and their affiliations is provided in SI Table 2. Information about temporal coverage, resolution, and gaps of the submitted data are shown for each region in SI Figures 1−19.

Team	Method	Sensors or products	Method references	Regions
**Bolch et al.**	Altimetry	ICESat	Bolch et al. (2013)[1]	GRL
**Foresta et al.**	Altimetry	CryoSat-2	Foresta et al. (2016)[2]	ISL
**Gardner et al.**	Altimetry	ICESat	Gardner et al. (2013)[3]	ACN, ACS, GRL, SJM, RUA, ANT
**Jakob et al.**	Altimetry	CryoSat-2	Jakob et al. (2021)[4], Jakob and Gourmelen (2023)[5]	ALA, ACN, ACS, GRL, ISL, RUA, ASC, ASW, ASE, SAN, ANT
**Khan et al.**	Altimetry	ICESat/-2	Khan et al. (2022)[6]	ACN, ACS, GRL, ANT
**Menounos et al.**	Altimetry	ICESat-2, GEDI, COP30	Menounos et al. (2024)[7]	WNA
**Nilsson et al.**	Altimetry	CryoSat-2, Envisat, ERS-1/2, ICESat/-2	Nilsson et al. (2022)[8], Nilsson and Gardner (2024)[9]	GRL
**Tepes et al.**	Altimetry	CryoSat-2	Tepes et al. (2021)[10]	ACN, ACS, ISL, SJM, RUA
**Treichler et al.**	Altimetry	ICESat	Treichler et al. (2019)[11]	ASC, ASW, ASE
**Abdullahi et al.**	DEM differencing	TanDEM-X	Abdullahi et al. (2019)[12], Wessel et al. (2016)[13]	GRL
**Andreassen et al.**	DEM differencing	Airborne LiDAR	Andreassen et al. (2016)[14]	SCA
**Braun et al.**	DEM differencing	TanDEM-X	Braun et al. (2019)[15]	TRP, SAN
**Brun et al.**	DEM differencing	ASTER	Brun et al. (2017)[16]	ASC, ASW, ASE
**Dussaillant et al.**	DEM differencing	ASTER	Dussaillant et al. (2019)[17]	TRP, SAN
**Hugonnet et al.**	DEM differencing	ArcticDEM, ASTER, REMA	Hugonnet et al. (2021)[17]	ALA, WNA, ACN, ACS, GRL, ISL, SJM, SCA, RUA, ASN, CEU, CAU, ASC, ASW, ASE, TRP, SAN, NZL, ANT
**King et al.**	DEM differencing	HMA DEM, SRTM	King et al. (2019)[18]	ASE
**Krieger et al.**	DEM differencing	SRTM, TanDEM-X	Abdel Jaber et al. (2019)[19]	SAN
**Piermattei et al.**	DEM differencing	ASTER, ArcticDEM, SPOT-5	Piermattei et al. (2024)[20]	ISL
**Seehaus et al.**	DEM differencing	SRTM, TanDEM-X	Seehaus et al. (2019)[21], Seehaus et al. (2020)[22]	TRP
**Sommer et al.**	DEM differencing	TanDEM-X	Sommer et al. (2020)[23], Sommer et al. (2022)[24]	RUA, CEU
**Zheng et al.**	DEM differencing	ArcticDEM	Zheng et al. (2018)[25]	ACN, ACS, GRL, ISL, SJM, SCA, RUA
**Menounos et al.**	Glaciological	In situ	n.a.	WNA
**WGMS**	Glaciological	In situ	WGMS (2023)[26]	ALA, WNA, ACN, ACS, GRL, ISL, SJM, SCA, RUA, ASN, CEU, CAU, ASC, ASW, ASE, TRP, SAN, NZL, ANT
**Harig et al.**	Gravimetry	GRACE/-FO	Harig and Simons (2016)[27], Beveridge et al. (2018)[28]	ALA, WNA, ACN, ACS, ISL, SJM, RUA, ASC, SAN, NZL

**Jacob et al.**	Gravimetry	GRACE	Jacob et al. (2012)[29]	ALA, WNA, ACN, ACS, ISL, SJM, RUA, ASC, ASN, CEU, CAU, ASC, ASW, ASE, TRP, SAN, NZL
**Pfeffer et al.**	Gravimetry	GRACE/-FO	Blazquez et al. (2018)[30]	ACN, ACS, ISL, SJM, RUA
**Richter et al.**	Gravimetry	GRACE	Richter et al. (2019)[31]	SAN
**Sasgen et al.**	Gravimetry	GRACE/-FO	Sasgen et al. (2022)[32]	ALA, WNA, ACN, ACS, ISL, SJM, RUA, ASC, ASN, CEU, ASC, ASW, ASE, TRP, SAN, NZL
**Sutterley et al.**	Gravimetry	GRACE/-FO	Sutterley and Velicogna (2019)[33], Sutterley et al. (2020)[34]	ALA, ACN, ACS, ISL, SJM, RUA, SAN
**Wouters et al.**	Gravimetry	GRACE/-FO	Wouters et al. (2019)[35]	ALA, WNA, ACN, ACS, ISL, SJM, RUA, ASC, ASN, CEU, CAU, ASC, ASW, ASE, TRP, SAN, NZL
**Box et al.**	Hybrid	GRACE	Box et al. (2018)[36]	ALA, ACN, ACS, ISL, SJM, RUA
**Colgan et al.**	Hybrid	GRACE, ICESat	Colgan et al. (2015)[37]	ACN, ACS, GRL
**Dussaillant et al.**	Hybrid	In situ, ASTER, SPOT-5/6/7, SRTM, TanDEM-X	Dussaillant et al. (2023)[38], Dussaillant et al. (2024, in review)[39]	ALA, WNA, ACN, ACS, GRL, ISL, SJM, SCA, RUA, ASN, CEU, CAU, ASC, ASW, ASE, TRP, SAN, NZL, ANT
**Huss et al.**	Hybrid	In situ, ASTER, ArcticDEM, REMA	Zemp et al. (2019)[40]	ALA, WNA, ACN, ACS, GRL, ISL, SJM, SCA, RUA, ASN, CEU, CAU, ASC, ASW, ASE, TRP, SAN, NZL, ANT
**Ke et al.**	Hybrid	SRTM, NASADEM, ICESat-2	Fan et al. (2023)[41]	ASC, ASW, ASE
**Miles et al.**	Hybrid	In situ, ASTER, ITS_LIVE	Miles et al. (2021)[42]	ASC, ASW, ASE
**Pálsson et al.**	Hybrid	In situ, Non-surface melt corrections	Aðalgeirsdóttir et al. (2020)[43]	ISL

SI Table 2 | Research teams and data contributors to GlaMBIE. Columns are sorted by research team in alphabetical order.

Team	Method	Data contributor (affiliation)
**Abdullahi et al.**	DEM differencing	S. Abdullahi (DLR), B. Wessel (DLR), C. Keller (DLR), M. Huber (DLR), U. Marschalk (DLR), A. Roth (DLR)
**Andreassen et al.**	DEM differencing	L. Andreassen (NVE), B. Kjøllmoen (NVE), H. Elvehøy (NVE)
**Bolch et al.**	Altimetry	T. Bolch (TU Graz), L. Sandberg Sørensen (DTU), N. Mölg (ENVEO)
**Box et al.**	Hybrid	J. Box (GEUS), W. Colgan (GEUS), B. Wouters (TU Delft), D. Burgess (NRCan), S. O'Neel (USGS), L. Thomson (Queen's Univ)
**Braun et al.**	DEM differencing	M. Braun (FAU), P. Malz (FAU), C. Sommer (FAU), D. Farías-Barahona (FAU), T. Sauter (FAU), G. Casassa (UMAG, DGA), A. Soruco, P. Skvarca (Glaciarium), T. Seehaus (FAU)
**Brun et al.**	DEM differencing	F. Brun (UGA), E. Berthier (LEGOS)
**Colgan et al.**	Hybrid	W. Colgan (GEUS), W. Abdalati (Univ Colorado), M. Citterio (GEUS), B. Csatho (SUNY-Buffalo), X. Fettweis (Univ Liège), S. Luthcke (NASA-GSFC), G. Moholdt (NPI), S.B. Simonsen (DTU), M. Stober (Stuttgart U.)
**Dussaillant et al.**	DEM differencing	I. Dussaillant (LEGOS, UGA), E. Berthier (LEGOS), F. Brun (UGA), M. Masiokas (IANIGLA), R. Hugonnet (LEGOS), V. Favier (UGA), A. Rabatel (UGA), P. Pitte (IANIGLA), L. Ruiz (IANIGLA)
**Dussaillant et al.**	Hybrid	I. Dussaillant (UZH), J. Bannwart (UZH), F. Paul (UZH), M. Zemp (UZH)
**Foresta et al.**	Altimetry	L. Foresta (Univ Edinburgh), N. Gourmelen (Univ Edinburgh)
**Gardner et al.**	Altimetry	A. Gardner (JPL), G. Moholdt (NPI), B. Wouters (TU Delft), E. Berthier (LEGOS)
**Harig et al.**	Gravimetry	C. Harig (UA), E. Cicero (UA)
**Hugonnet et al.**	DEM differencing	R. Hugonnet (UW, LEGOS, ETH, WSL), R. McNabb (Ulster Univ, UiO), E. Berthier (LEGOS), B. Menounos (UNBC, Hakai Inst), C. Nuth (UiO, FFI), L. Girod (UiO), D. Farinotti (ETH, WSL), M. Huss (ETH, WSL, UFR), I. Dussaillant (LEGOS, UZH), F. Brun (IGE), A. Kääb (UiO)
**Huss et al.**	Hybrid	M. Huss (ETH, WSL, UFR), R. Hugonnet (UW), D. Farinotti (ETH, WSL)
**Jacob et al.**	Gravimetry	T. Jacob (CU), J. Wahr (CU), T. Pfeffer (CU), S. Swenson (NCAR).<br>Data preparation: I. Sasgen (AWI)
**Jakob et al.**	Altimetry	L. Jakob (Earthwave), N. Gourmelen (Earthwave, Univ Edinburgh), S. Dubber (Earthwave)
**Ke et al.**	Hybrid	C. Ke (NJU), Y. Fan (NJU), X. Zhou (MTECH), X. Shen (NJU)
**Khan et al.**	Altimetry	S. Khan (DTU), W. Colgan (GEUS), J. Hassan (DTU)
**King et al.**	DEM differencing	O. King (NU), A. Bhattacharya (JIS Univ, JISIASR), R. Bhambri (WIHG), T. Bolch (TU Graz)
**Krieger et al.**	DEM differencing	L. Krieger (DLR, IMF), D. Floricioiu (DLR, IMF)
**Menounos et al.**	Altimetry	B. Menounos (UNBC), A. Gardner (JPL), C. Florentine (USGS), A. Fountain (PSU)
**Menounos et al.**	Glaciological	B. Menounos (UNBC), A. Gardner (JPL), C. Florentine (USGS), A. Fountain (PSU)
**Miles et al.**	Hybrid	E. Miles (UZH, UFR, WSL), M. McCarthy (WSL), A. Dehecq (IGE), M. Kneib (IGE, UIBK), S. Fugger (WSL), F. Pellicciotti (WSL)
**Nilsson et al.**	Altimetry	J. Nilsson (JPL), A. Gardner (JPL)
**Pálsson et al.**	Hybrid	F. Pálsson (HI), A. Gunnarsson (Landsvirkjun), G. Aðalgeirsdóttir (HI), E. Magnússon (HI), T. Thorsteinsson (IMO), J. Belart (HI, IMO, LEGOS), T. Jóhannesson (IMO), H. Hannesdóttir (IMO), O. Sigurðsson (IMO), B. Einarsson (IMO), H. Haraldsson (Landsvirkjun), H. Björnsson (HI)
**Pfeffer et al.**	Gravimetry	J. Pfeffer (Magellium), A. Blazquez (CNES), B. Coupry (Magellium), E. Berthier (LEGOS)
**Piermattei et al.**	DEM differencing	L. Piermattei (UZH, WSL, UiO), D. Treichler (UiO), E. Mattea (UFR), R. McNabb (Ulster Univ)
**Richter et al.**	Gravimetry	A. Richter (MAGGIA, CONICET), A. Romero (MAGGIA, CONICET), F. Suad Corbetta (MAGGIA, CONICET), T. Döhne (TUD), M. Horwath (TUD)
**Sasgen et al.**	Gravimetry	I. Sasgen (AWI), S. Cruz Bacca (AWI)
**Seehaus et al.**	DEM differencing	T. Seehaus (FAU), M. Braun (FAU), P. Malz (FAU), C. Sommer (FAU)
**Sommer et al.**	DEM differencing	C. Sommer (FAU), T. Seehaus (FAU), M. Braun (FAU), P. Malz (FAU)
**Sutterley et al.**	Gravimetry	T. Sutterley (APL-UW), I. Velicogna (UCI, JPL), G. A (UCI), C. Liang (UCI)
**Tepes et al.**	Altimetry	P. Tepes (Univ Edinburgh), N. Gourmelen (Univ Edinburgh)
**Treichler et al.**	Altimetry	D. Treichler (UiO), A. Kääb (UiO), N. Salzmann (UFR), C. Xu (UiO)
**WGMS**	Glaciological	World Glacier Monitoring Service (WGMS), Zurich, Switzerland, staff members (in alphabetic order): P. Alean-Kirkpatrick, J. Bannwart, F. Denzinger, I. Dussaillant, L. Fischer, M. Fischer, R. Frauenfelder, H. Frey, I. Gärtner-Roer, W. Haeberli, M. Hoelzle, F. Hüsler, J. Landmann, R. Le Bris, G. Leysinger Vieli, A. Linsbauer, H. Machguth, N. Mölg, K. Naegeli, J. Noetzli, S. Nussbaumer, F. Paul, P. Rastner, J. Roth, S. Suter, E. Welty, M. Zemp.<br><br>National correspondents and principal investigators (in alphabetic order) contributing to the glaciological mass balance data since 2000: J. Abermann (ASIAQ), A. Ahlstrøm

(GEUS), A. Ajikeev (Kyrgyzhydromet), A. Alberti (SGL), M. Andersen (GEUS), B. Anderson (VUW, SGEES, ARC), L. Andreassen, (NVE), T. Angchuk (JNU), L. Antonini, M. Arenillas (Ingeniería 75), F. Armiento (CGI), Á. Ayala (DGA), M. Azam (LGGE), E. Azisov (CAIAG), M. Bachmann (USGS), R. Baikhadzhaev (Kyrgyzhydromet), S. Bajracharya(ICIMOD), E. Baker (USGS), M. Barandun (UFR, CICADA, CAIAG), A. Barbolla (CGI), G. Barcaza (DGA), C. Baroni (CGI, UPDES), A. Barrueto (UO), H. Basagic (PSU), A. Bauder (VAW/ETH, GLAMOS), M. Beedle (USGS), S. Belekov (Kyrgyzhydromet), S. Bellagamba (ISPESL), A. Bera (SGL), J. Berkhoff (AUC), V. Bertoglio (CGI), W. Bidlake: (USGS), A. Bjoerk (UC, NHMD), H. Björnsson (UI, IES), P. Boccabella (CGI), K. Bollen (USGS), M. Bonnefoy: (IRSTEA, ETNA), L. Braun (BASH, CGG), D. Brus (FMI), F. Bruschi (UniPG), F. Buglio (DGA), D. Bulanti (SGL), D. Burgess (NRCan, GSC), R. Böhm (ZAMG), C. Bøggild (GEUS), G. Cabrera (IANIGLA), T. Caira (CGI), S. Camargo (GTF), B. Cao (LZU, WEL), D. Cappelletti (CGI, UniPG), D. Carrión (DGA), L. Carturan: (SAT, UniPG), G. Casartelli, G. Casassa (DGA), D. Cat Berro (SMI), J. Ceballos Liévano (CGI), P. Cherkasov (MESIG), R. Chernov (IGRAS), W. Chunzu (CAREERI, CAS), D. Cimini (MUR, NRC, IMEA), M. Citterio (GEUS), M. Ciucci (ISPESL), A. Clark (USGS), G. Cobos Campos: (MMA, TUV, DIT), A. Cochachin Rapre (UGRH, INRENA), J. Cogley (TU, DG), D. Colombarolli (SGL), T. Condom (IGE, LGGE, UGA), J. Conejo: (Ingeniería 75), L. Copland (UODG), F. Covi (UIDACS), R. Cruz Encarnación (MADI, NWA), N. Cullen (ODG), B. Cáceres Correa (INAMHI), Y. Cárcamo, P. D'Aquila (CGI, CNSAS), M. Daiyrov (CAIAG), H. De Angelis (AAIDG), M. De Zaiacomo (SGL), M. Demuth (NRCan, GSC), A. Di Lullo (AfBs), R. Dinale (AfBs), G. Diolaiuti (CGI), D. Dobhal (WIHG), L. Dávila Roller (UGRH, INRENA), M. Ecclestone (TUDG), K. Echelmeyer (UAFGI), M. Ednie (JNP, NRCan, GSC), B. Einarsson (IMO), N. Elagina (IGRAS), H. Elvehøy (NVE), R. Engeset (NVE), P. Eriksson: (SUDPG), B. Ermenbayev (NAS KR, IWP, TShMRC), E. Ermolin (AAIDG), A. Erofeev (TSUDG), F. Escobar Caceres (DGA), M. Esenaman uulu (CAIAG), D. Eshmiratov (MID, UIF, CICADA), A. Espinoza (DGA), L. Espizua (IANIGLA), G. Esposito (MUR, NRC, IAPR), D. Fagre (USGS), D. Farinotti (WSL, ETH), F. Fernandoy Pedreros (ABU), L. Ferri Hidalgo (IANIGLA), A. Fischer (OEAW, ACINN), S. Fitzsimons (UODG), C. Florentine (USGS), J. Foreman (USGS), F. Fornengo (SMI), A. Fountain (PSUDG), G. Franchi (AfBs, CGI), B. Francou (IRD, LGGE), J. Friedt (NCSR, UFC), K. Fujita (NUGSES), K. Fukui (NUGSES, TCSM), M. Funk (ETH), M. Gaddo (Meteotrentino), A. Gafurov (GRCG), P. Gallo (SGL), S. Galos: (AfBs, ACINN), B. Galton-Fenzi (AAD), H. Gargantini (IANIGLA), C. Garin (DGA), P. Ginot (IGE, LGGE, UGA), P. Glowacki (PASIG), J. Gonzaléz (DGA), A. Grilli (CGI, UniPG), M. Griselin (NCSR, UFC), W. Guan (LZU, WEL), A. Gubanov (MSU, RFBR), A. Gunnarsson (NPC), T. Gurung (ICIMOD), J. Gutierrez (DGA), J. Gómez (INRENA, UGRH), J. Hagen (UIO), W. Hagg (LMU), A. Halimov (NAST), E. Hanna (USDG), H. Hannesdóttir (IMO), H. Haraldsson (NPC), F. Hardmeier (UZH), L. Hartl (HDLT), B. Hasholt (UCDGG), S. Haug (NVE), A. Hedayatullah (KUGSDH), C. Helanow (SUDPG, TRS), K. Helfricht (OEAW), A. Henz (UZH), J. Hernández (DGA), J. Heyer (UZH), P. Heyer (UZH), E. Hodel: (ETH, GLAMOS), P. Holmlund (SUDPGQG), Y. Huian (CAS, CAREERI), M. Huss (ETH, UFR, GLAMOS), P. Huybrechts (FUB), B. Hynek (GSA, UGIGRS, ZAMG), D. Ignatiuk (USFNS), R. Iturraspe (CADIC, NUTF), C. Iurisci (CGI), P. Jansson (SUDPGQG, TRS), Z. Jing (CAS, CAREERI), S. Joshi (ICIMOD), I. Juen (ACINN), K. Kabutov (NAST), G. Kappenberger (ETH, GLAMOS, MeteoSwiss), T. Karlin (SUDPGQG), N. Kasatkin (CARGC), G. Kaser (ACINN), A. Kayumov (NAST), R. Kenzhebaev (CAIAG), J. Keqin (CAS, CAREERI), A. Kerimov (IGRAS), T. Kerr (NIWA), I. Khmelevskoy (IGRAS), N. Kirchner (SU, TRS), B. Kjøllmoen (NVE), N. Knudsen (AUDG), J. Kohler (NPI, FRAM Centre), K. Krantz (UIO), D. Kriegel (GRCG), R. Krimmel (USGS), M. Kronenberg (UFR), M. Kuhn (ACINN), S. Kutuzov (IGRAS), R. Körner (NRCan, GSC, PCSP), C. Lambiel (ULS), K. Langley (ASIAQ, GEM), M. Larrabee (NPS, NOCA), S. Larsen, M. Laska (USFNS), E. Lastrada (SPESA), I. Lavrentiev (IGRAS), J. Lea (ULSES), J. Leiva (IANIGLA), G. Leysinger-Vieli (UZH), H. Li (CAS, CAREERI, NIEER, ITP), Z. Li (CAS, CAREERI, NIEER), S. Liu (CAS, CAREERI), L. Lombardi (IMI), B. Luks (PASIG), A. Mackintosh (VUW, SGEES), K. Makarevich (MESIG), A. Makeshov (NAS KR, IWP, TShMRC), J. Malecki (AMU), H. Mamirov (UFR, CICADA), M. Manni (SGL), R. March (USGS), L. Margonari (AAIDG), A. Marino (ISPESL), S. Marinsek (IAADG), G. Markl (ACINN, HDLT), T. Markussen (GEUS), C. Marshall (USGS), M. Martinelli (Engeoneering), F. Marzano (CETEMPS), M. Masserini (SGL), D. Massoni (CGI, GPNP), E. Mattea (UFR), B. Mavlyudov (IGRAS), C. Mayer (BASH, CGG), C. McNeil (JIRP, USGS), M. Mehta (WIHG), J. Mendoza (HUSA, IHH), M. Meneghel (CGI, SAT), L. Mercalli (SMI), S. Mernild (LANL, COSIM, NERSC), L. Miguel, M. Miller (USGS), M. Montini (CGI), L. Moreau: (EDYTEM, GlacioLab), U. Morra di Cella (ARPA, VDA), L. Motta (UTDES), M. Munari (AfBs), Y. Muravyov (IVS, RAS), T. Musaev (CAIAG, Kyrgyzhydromet), A. Muñoz (Ingeniería 75), Y. Narozhniy (TSU), F. Navarro (AMINSA, TUM, ETSIT), H. Navruzshoev (NAST), A. Neureiter (CIMG, ZAMG), S. Niebuhr (UMN, PGC), S. Nikitin (IGRAS), Y. Nocua Ruge (IHMES), G. Nosenko (IGRAS), S. O'Neel (USGS), C. Oberschmied (AfBs), S. Omurbekov (NAS KR, IWP, TShMRC), M. Oreggioni (SGL), M. Ortelli (SGL), A. Osmonov (CAIAG), A. Ospina (IHMES), B. Pan (LZU, WEL), M. Pecci (CGI, ISPESL, PCM, DARA, DARAS), A. Pedrero (Ingeniería 75, SPESA), E. Peitzsch (USGS), B. Pelto (BCP, PC, UNBC, JIRP, NCGCP, USGS), A. Peracino (CGI), I. Peri (SGL), E. Perroy (ISD), M. Petlicki (PASIG), D. Petrakov (MSUFG), M. Petrov (MID, UFR, CICADA), R.

		Pettersson (UPU, DES), S. Pignotti (IMI), P. Pitte (IANIGLA), V. Pohjola (UTU, UPU), V. Popovnin (MSU, RFBR), R. Porta (SGL), C. Portocarrero Rodríguez (INRENA, UGRH), G. Prandi (SGL), B. Pratap (WIHG), R. Prinz (ACINN, AfBs), D. Puczko (PASIG), P. Puga (DGA), C. Puigdomenech (AAIDG), H. Purdie (UCDG), F. Pálsson (UIIES), J. Quinteros (DGA), A. Rabatel (IRD, LGGE, UGA), A. Ramanathan (JNU, SES, LGGE), J. Ramírez (INGEOMINAS), D. Ramos (GTF), R. Rampazzo (SGL), E. Raparelli (CETEMPS), X. Ravanat (NRIAFE), T. Redpath (UOSS), K. Reingruber (OEAW, IGF, Energie AG), S. Reisenhofer (ZAMG), P. René (AM), L. Reynaud (NCSR), D. Richard (CEMAGREF), J. Riedel (NPS, NOCA), A. Rinaldini (ISPESL), A. Rivera (CECS), J. Rodriguez (AUC), F. Rojas (IHMES), E. Roland (NVE), S. Romshoo (DST), G. Rosqvist (SUDPGQG), G. Rossi (APB, AfBs, SAT, CGI), A. Rossotto (CGI, GPNP), O. Rototayeva (IGRAS), A. Ruddell, M. Ruffoni (SGL), L. Ruiz (IANIGLA), L. Ruvo (SGL), A. Sajood (KUGFDH), D. Sakakibara (HUILTS), T. Saks (MID, UFR, CICADA), R. Salvatori (CGI), N. Salzmann (UZH, UFR), C. Sangewar (GSI), L. Sass (USGS), S. Satarov (CAIAG), R. Satylkanov (NAS KR, IWP, TShMRC), D. Sayakpayev (NAS KR, IWP, TShMRC), M. Schaefer (AUC, ICFM), W. Schöner (ZAMG), R. Scotti (SGL), J. Seco (AAIDG), A. Segovia (DGA), B. Seiser (HDLS), I. Severskiy (MESIG), P. Sharma (NCPOR), V. Shatravin (NAS KR, IWP, TShMRC), T. Sherpa (ICIMOD), J. Sicart (IGE, LGGE, UGA), O. Sigurðsson (NEAHS), P. Sirguey (UODG), D. Six (IGE, LGGE, UGA), P. Skvarca (AAIDG), H. Slupetzky (HDLS, PLUS), S. Smiraglia (CGI, IAC), A. Smirnov (MSU, IGRAS, TSU), I. Sobota (NCU, FES), L. Sold (UFR), A. Soruco (IGE, IGEMA), C. Springer (ZAMG), U. Steinegger (ETH, GLAMOS), M. Stocker-Waldhuber (AfBs, TNP, OEAW), J. Strelin (CADIC, AAI, FCEFyN), M. Strudl, D. Stumm (ICIMOD, UODG), L. Tarasova (IGRAS), E. Thibert (CEMAGREF, IRSTEA, ETNA), L. Thomson (QUDGP, ICELab, uOttawa), F. Tolle (NCSR, UFC), D. Trabant (USGS), A. Trenti (PATUPP, MeteoTrentino), M. Truffer (UAF), B. Trüssel (UAF), T. Tshering (NCHM), N. Tsheten (NCHM), P. Tuccella (CETEMPS), G. Umirzakov (MID, UFR, CICADA), R. Unger (ZAMG), R. Usubaliev (CAIAG), M. Vallon (NCSR), L. Vargo (VUW, SGEES, ARC), F. Villa (SGL), C. Vincent (NCSR, INSU, IGE, LGGE, UGA), S. Vorogushyn (GRCG), F. Vosidov (NAST), P. Wagnon (IGE, LGGE, UGA), J. Wang (LZU, WEL), W. Wang (CAS, CAREERI), N. Wangchuk (NCHM), Z. Wen (LZU, WEL), E. Whorton (USGS), H. Wiesenegger (LSHD), A. Yakovlev (UzHydromet, UzGIP), H. Yang (CAS, CAREERI), W. Yang (CAS, ITP), B. Zagel (LSHD, PLUS), L. Zalazar (IANIGLA), G. Zanon (UPDG), T. Zanoner (IMI), M. Zapata (INRENA, UGRH), T. Zendrini (SGL), Z. Zhou (CAS, CAREERI), G. de Felice (IAC), E. de Pisón Stampa (MMA, PHUSA), S. de Villiers (WNUAS), M. den Ouden (UTU, UPU), T. di Fiore (AQCF), W. van Pelt (UTU, UPU), T. van Ommen (AAD), Þ. Þorsteinsson (IMO).
**Wouters et al.**	Gravimetry	B. Wouters (UU, TU Delft), A. Gardner (JPL), G. Moholdt (NPI)
**Zheng et al.**	DEM differencing	W. Zheng (NCU), M. Willis (CU), M. Pritchard (Cornell Univ), I. Howat (OSU)

Description of SI Figures 1−19

Supplementary Data Figures 1−19 show data submissions and GlaMBIE results for the 19 glacier regions. The subplots show the sample size of submitted estimates (a) with combined results within and among methods as annual (b) and cumulative (c) specific mass changes. Submitted and combined estimates are shown as cumulative specific mass changes for altimetry (d), DEM differencing & glaciological observations (e), and gravimetry (f). In the legends, we indicate for each data submission if the variability [v] and/or trend [t] were used to calculate the combined estimate. Datasets not used are shown as grey dotted lines. Data submissions from hybrid approaches are marked (*) and shown in subplots of the assigned observation method. Uncertainties are given at 95% confidence intervals.

SI Figure 1 | Data submissions and results for Alaska.

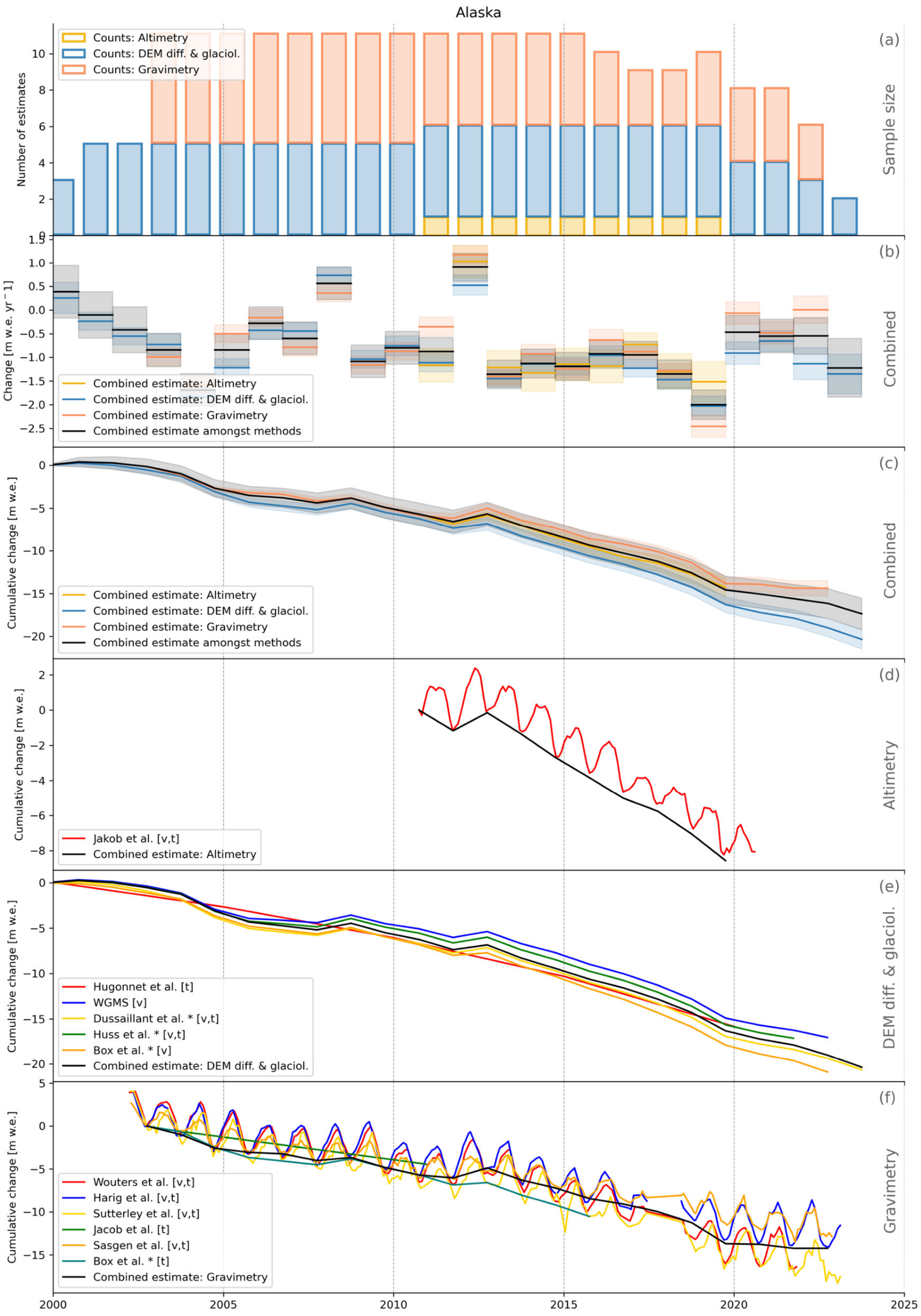

SI Figure 2 | Data submissions for Western Canada & USA

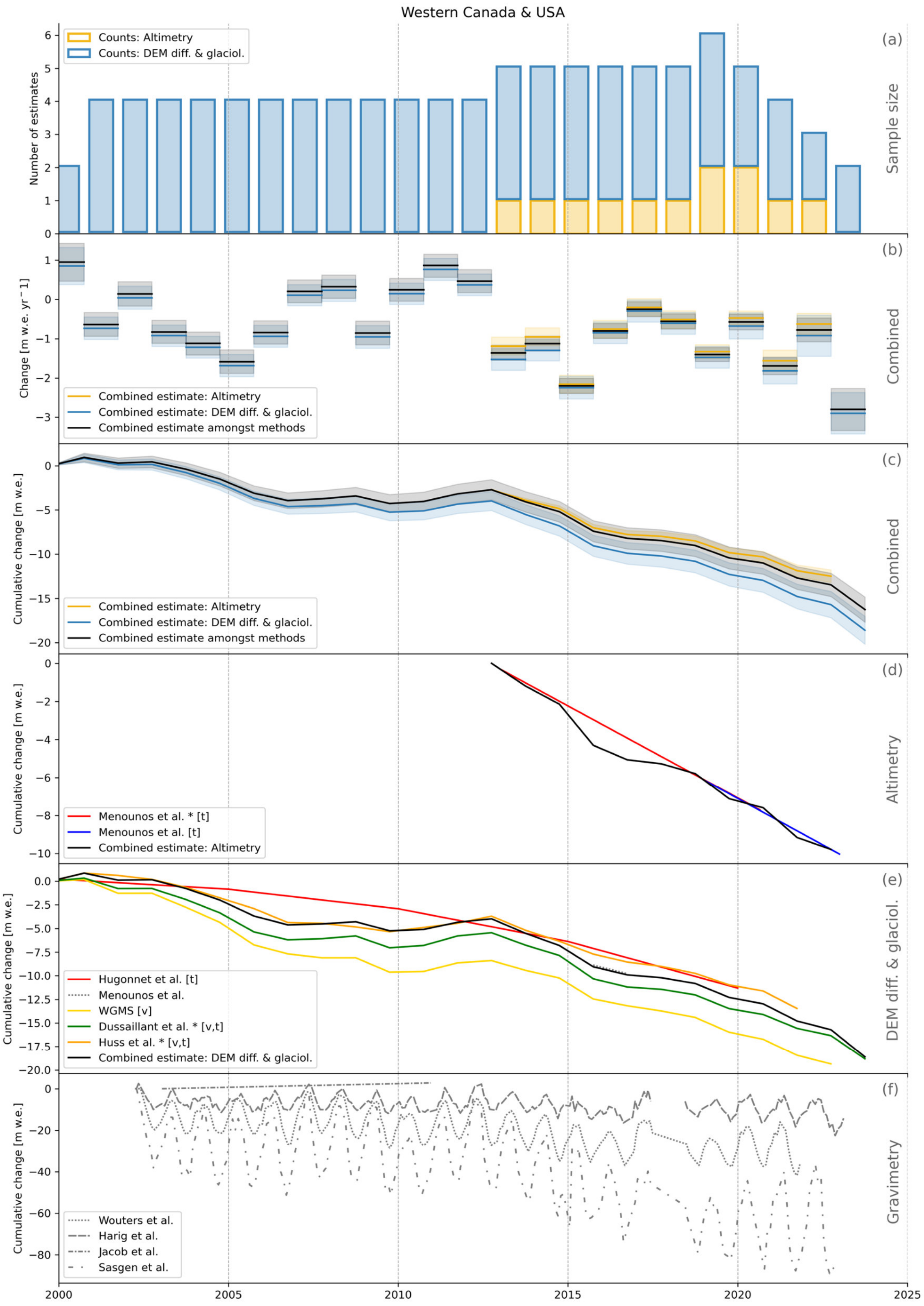

SI Figure 3 | Data submissions for Arctic Canada North

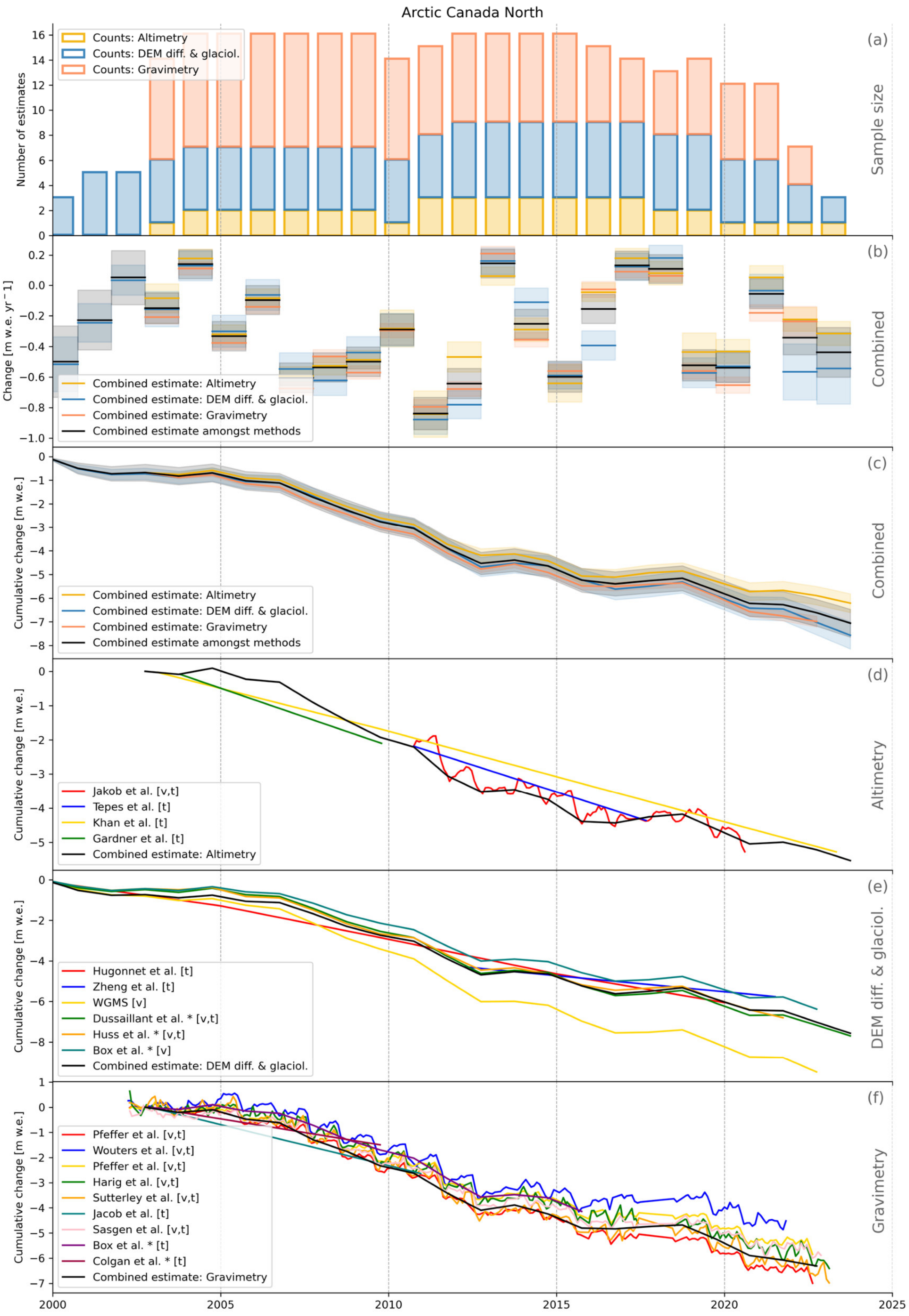

SI Figure 4 | Data submissions for Arctic Canada South

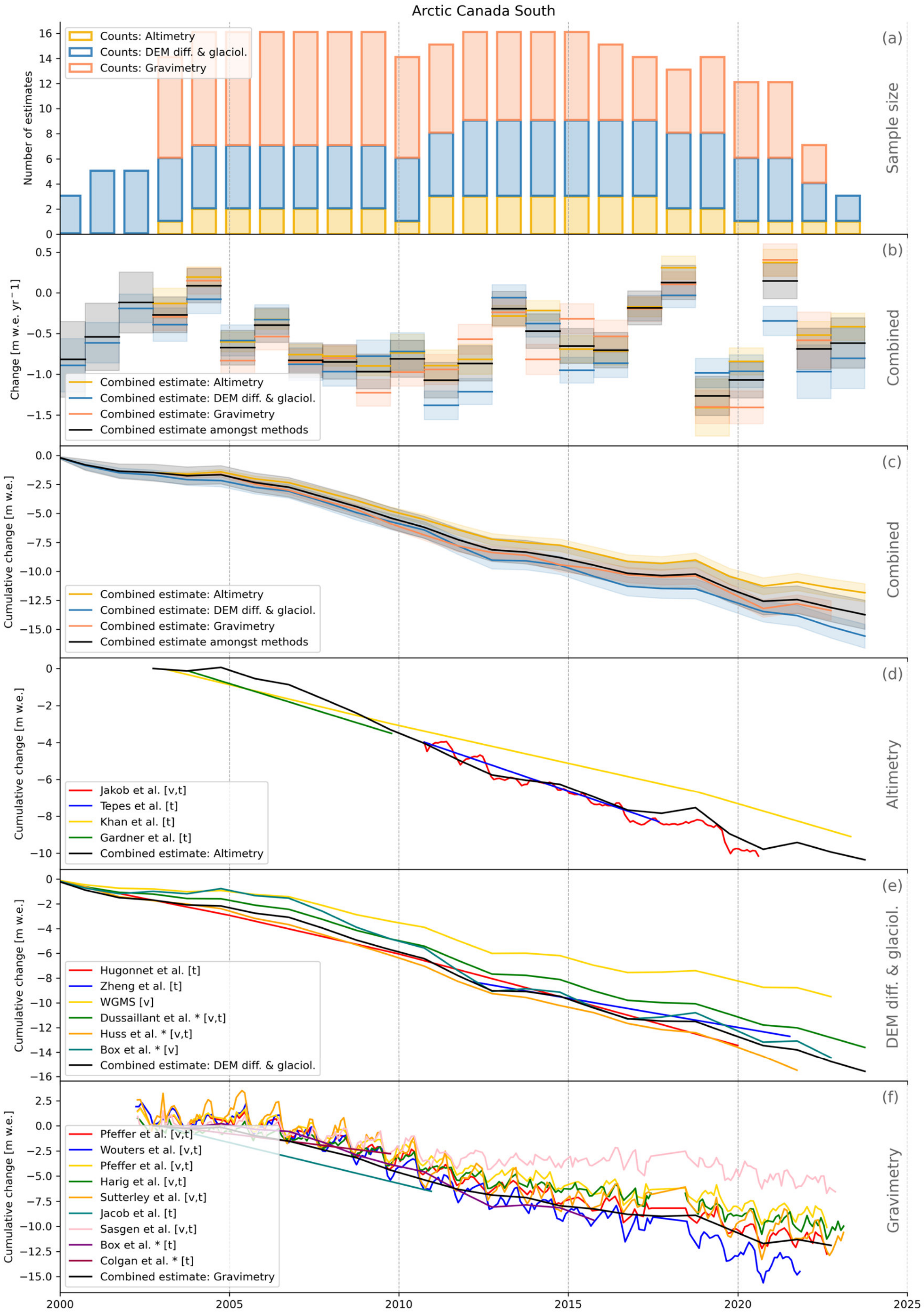

SI Figure 5 | Data submissions for Greenland Periphery

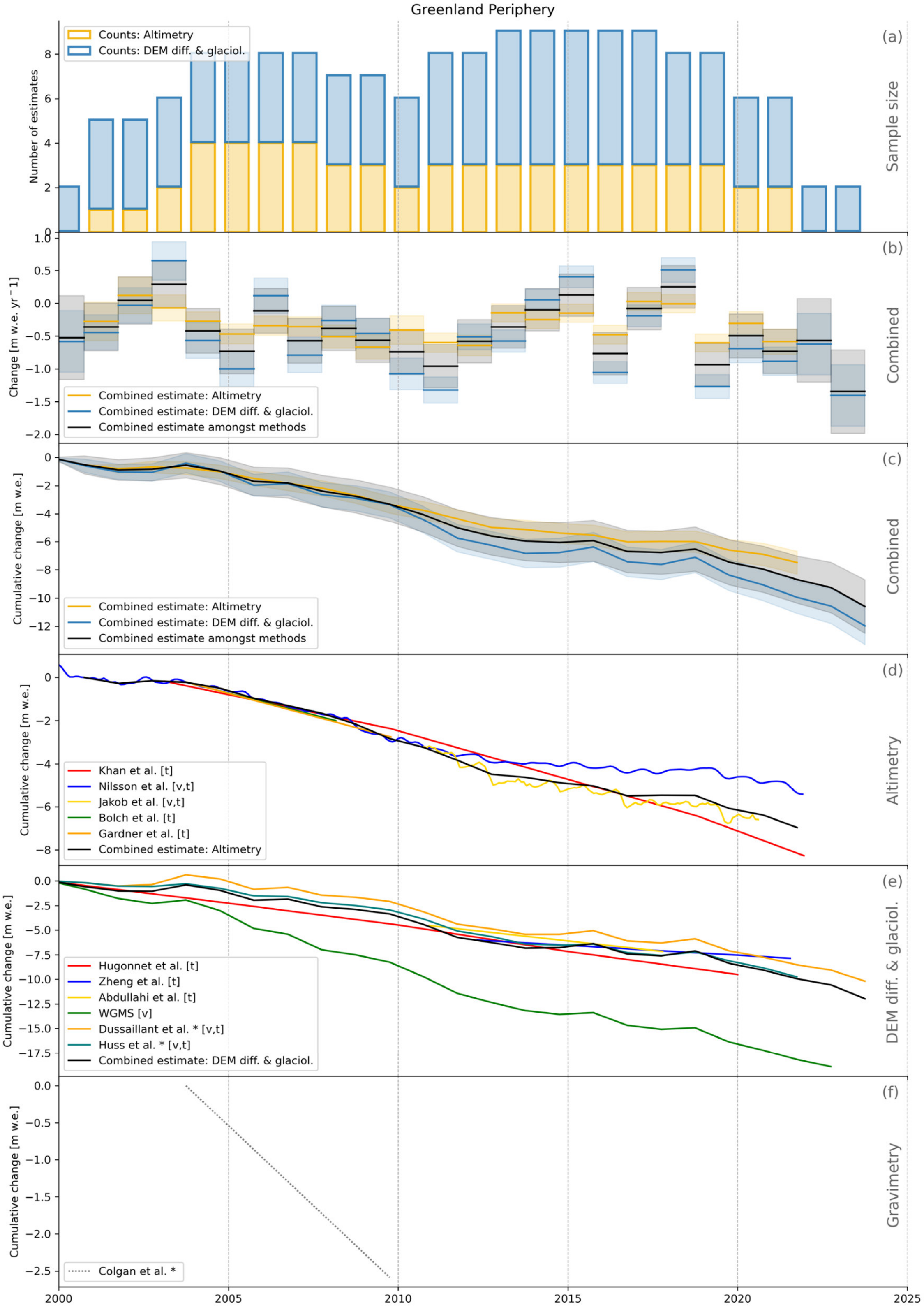

SI Figure 6 | Data submissions for Iceland

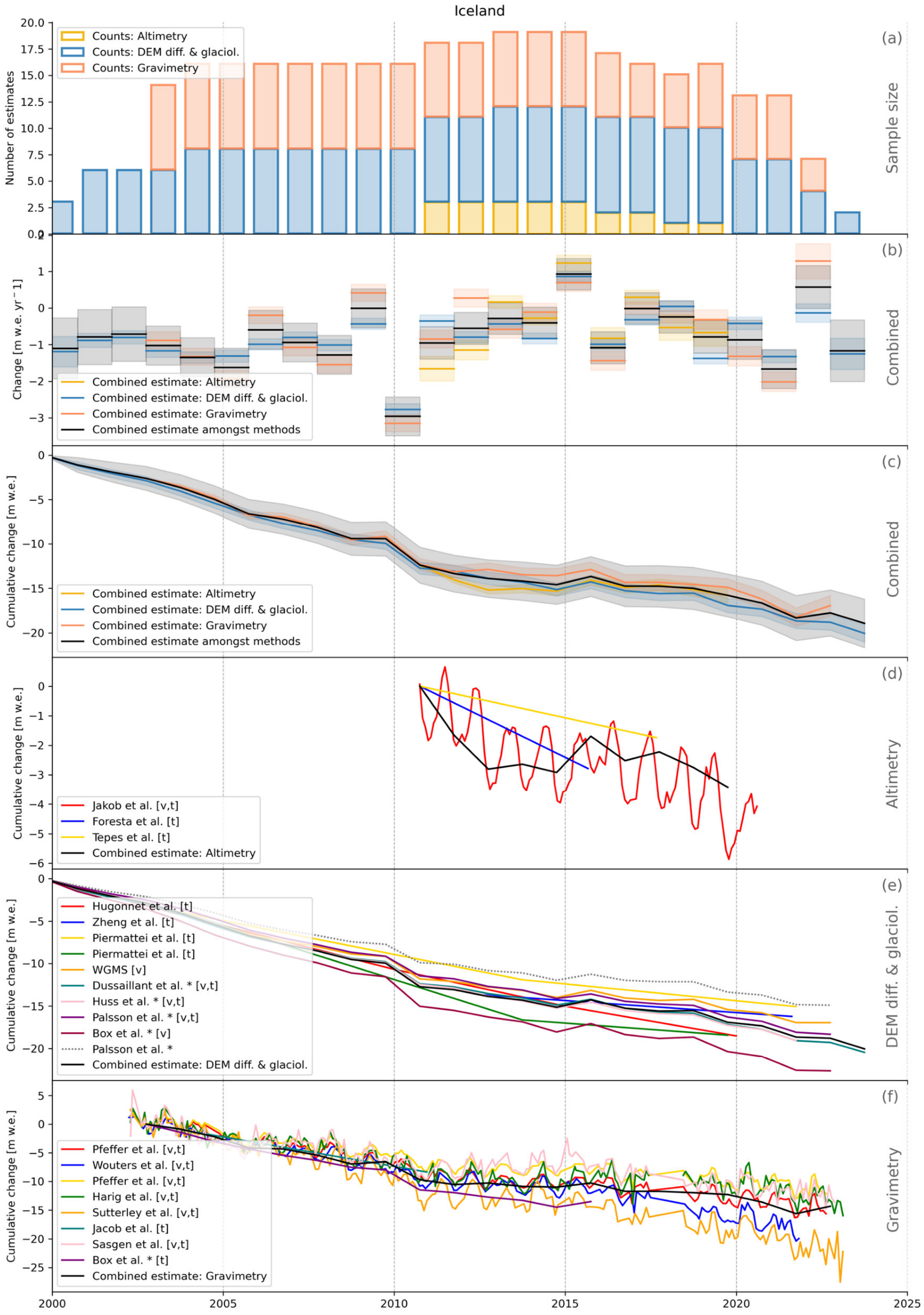

SI Figure 7 | Data submissions for Svalbard & Jan Mayen

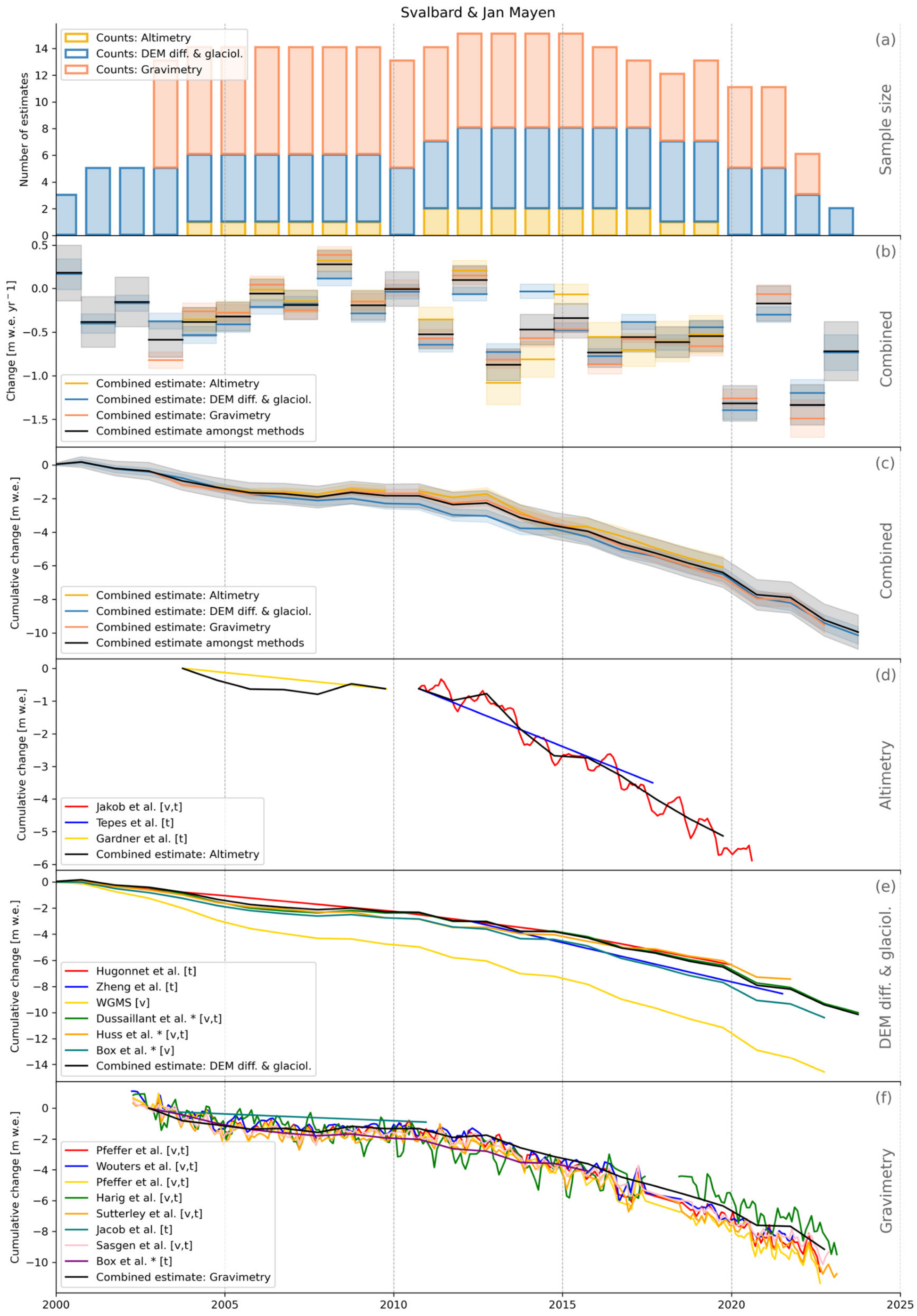

SI Figure 8 | Data submissions for Scandinavia

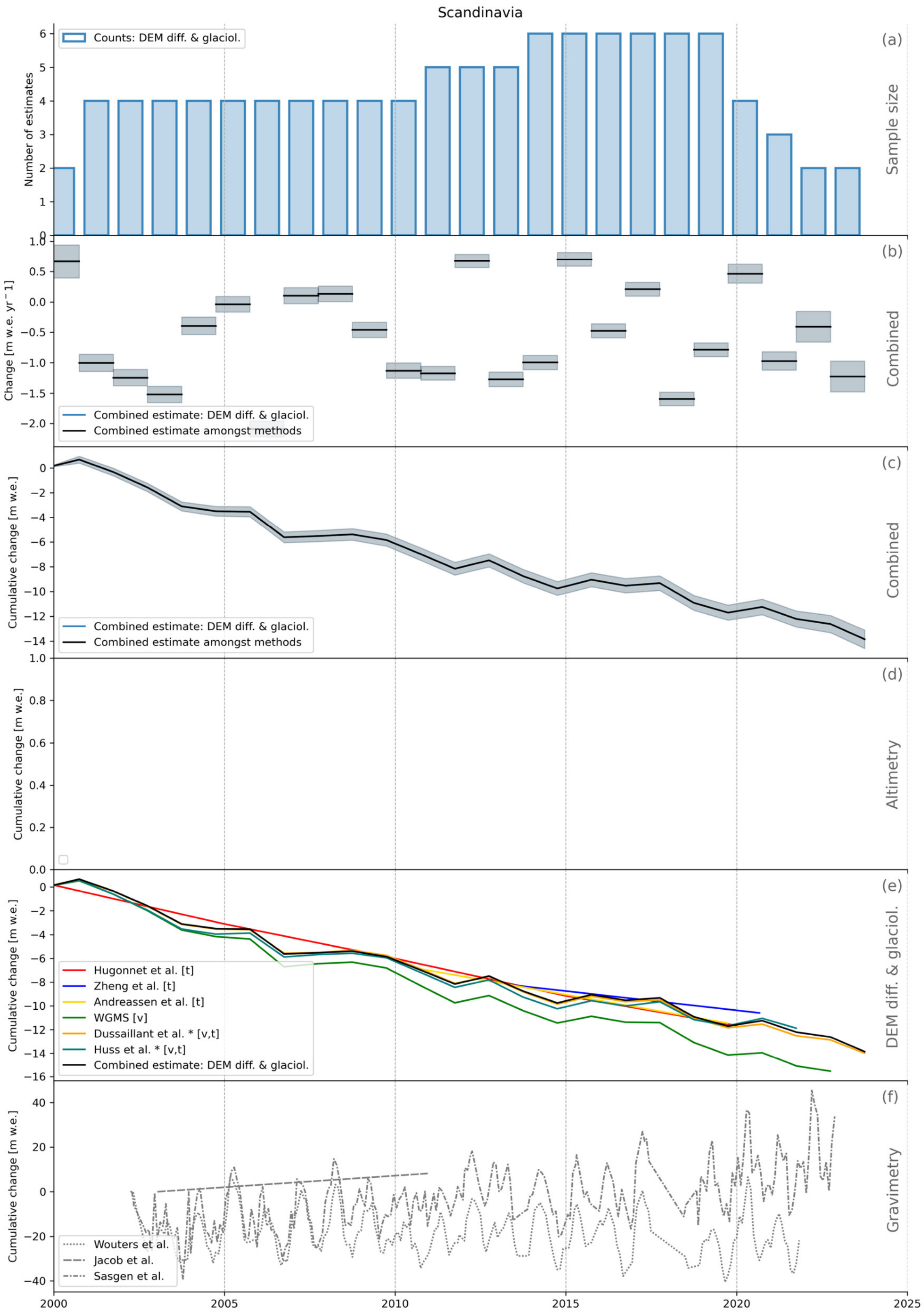

SI Figure 9 | Data submissions for Russian Arctic

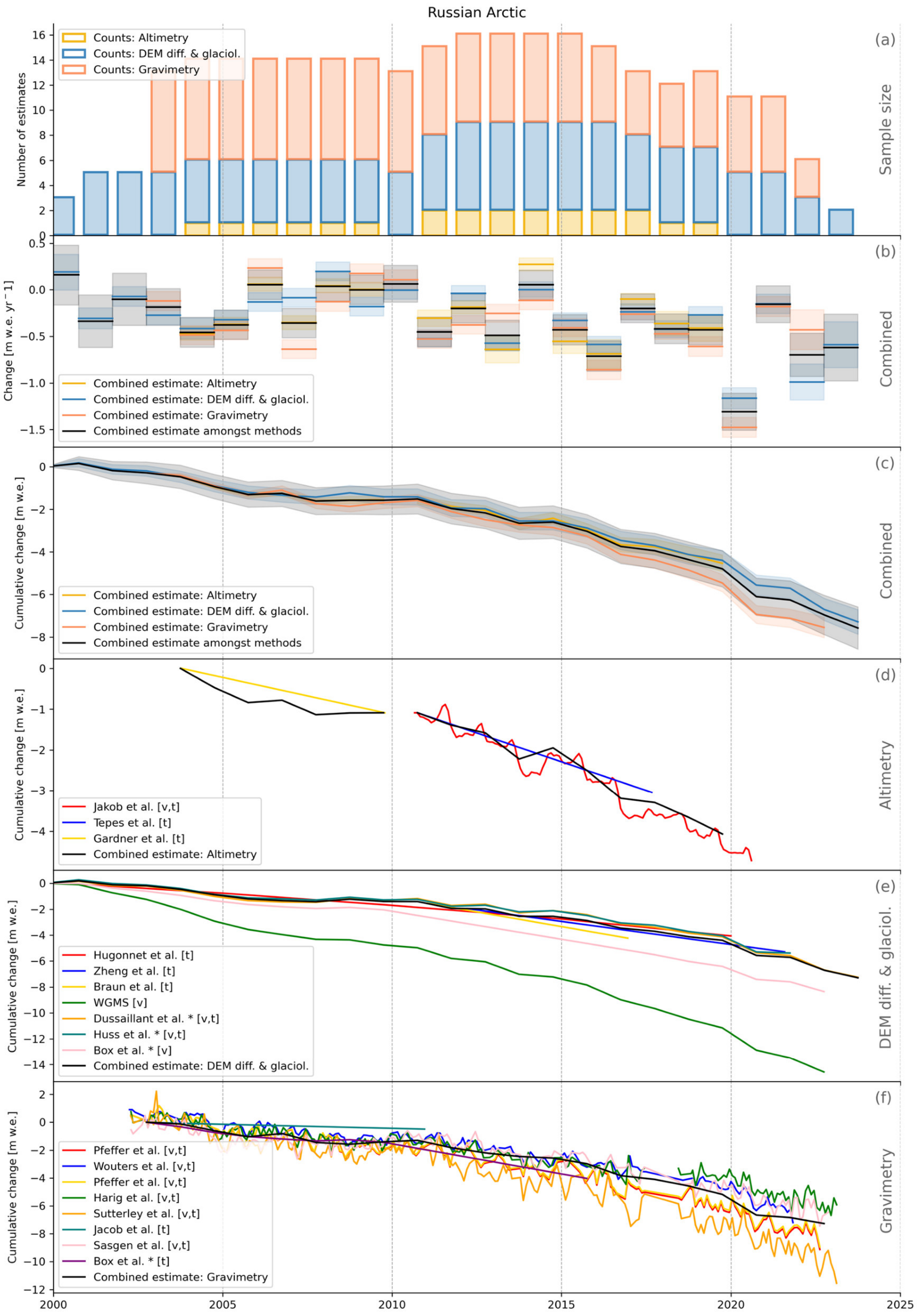

SI Figure 10 | Data submissions for North Asia

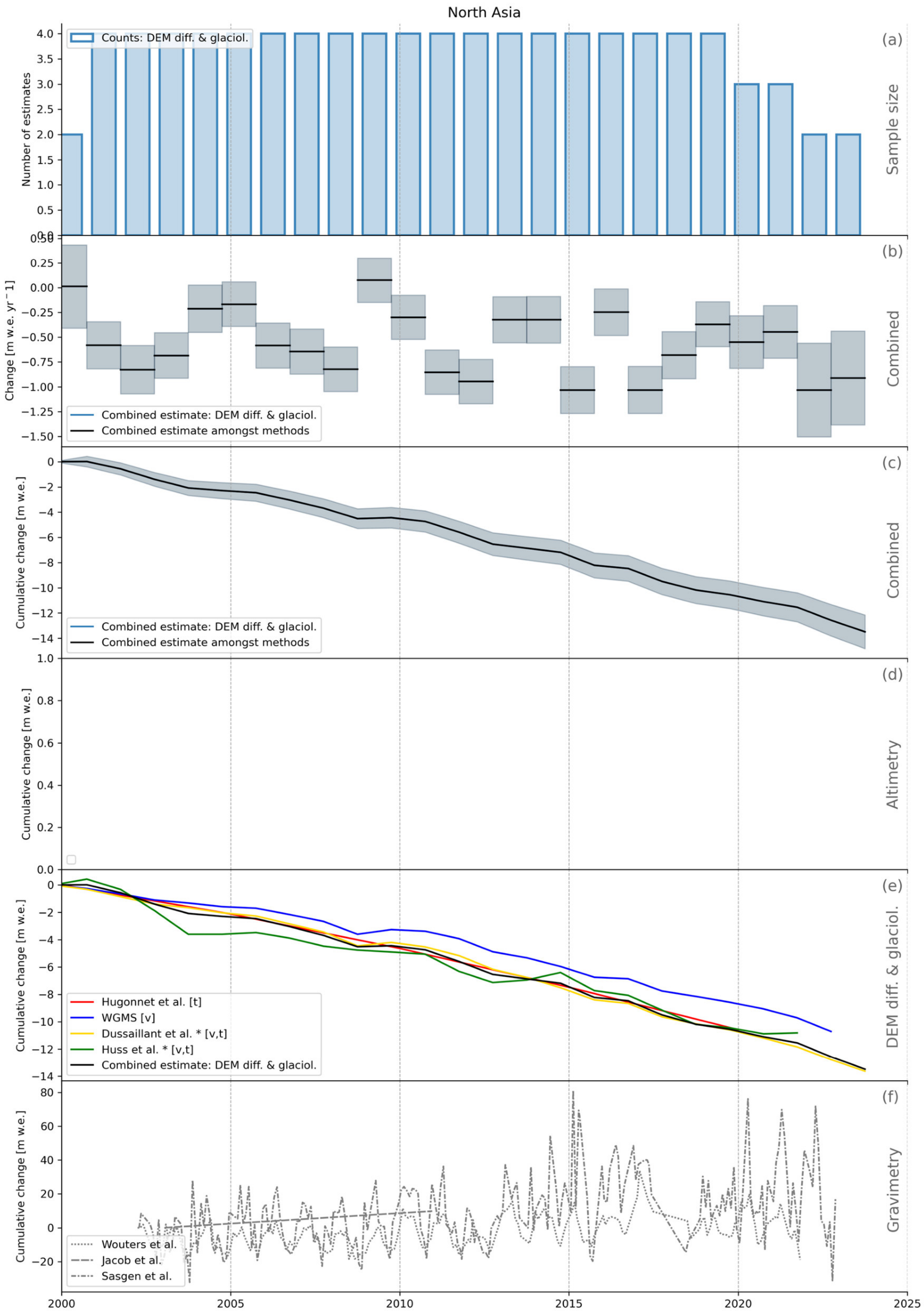

SI Figure 11 | Data submission for Central Europe

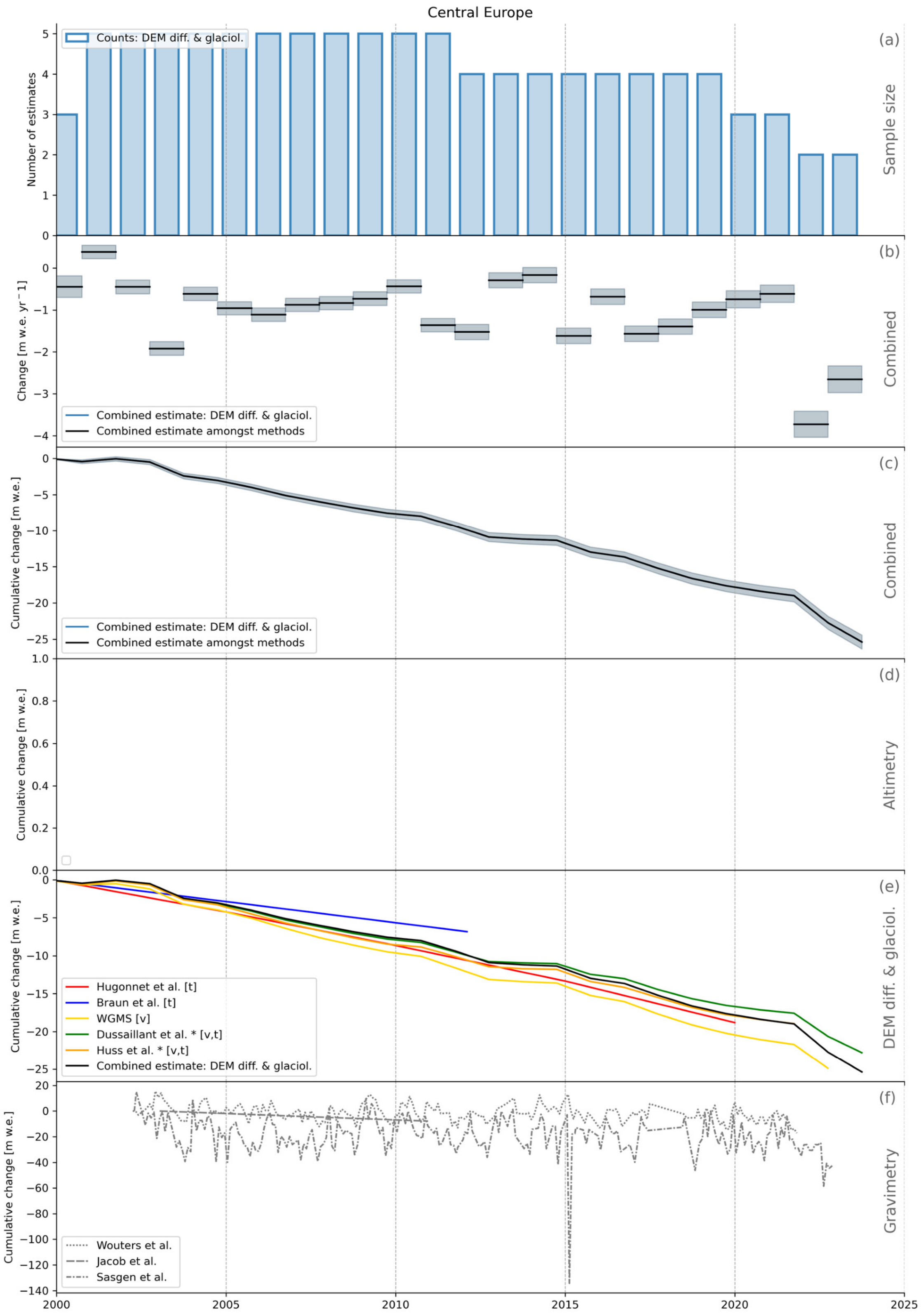

SI Figure 12 | Data submission for Caucasus & Middle East

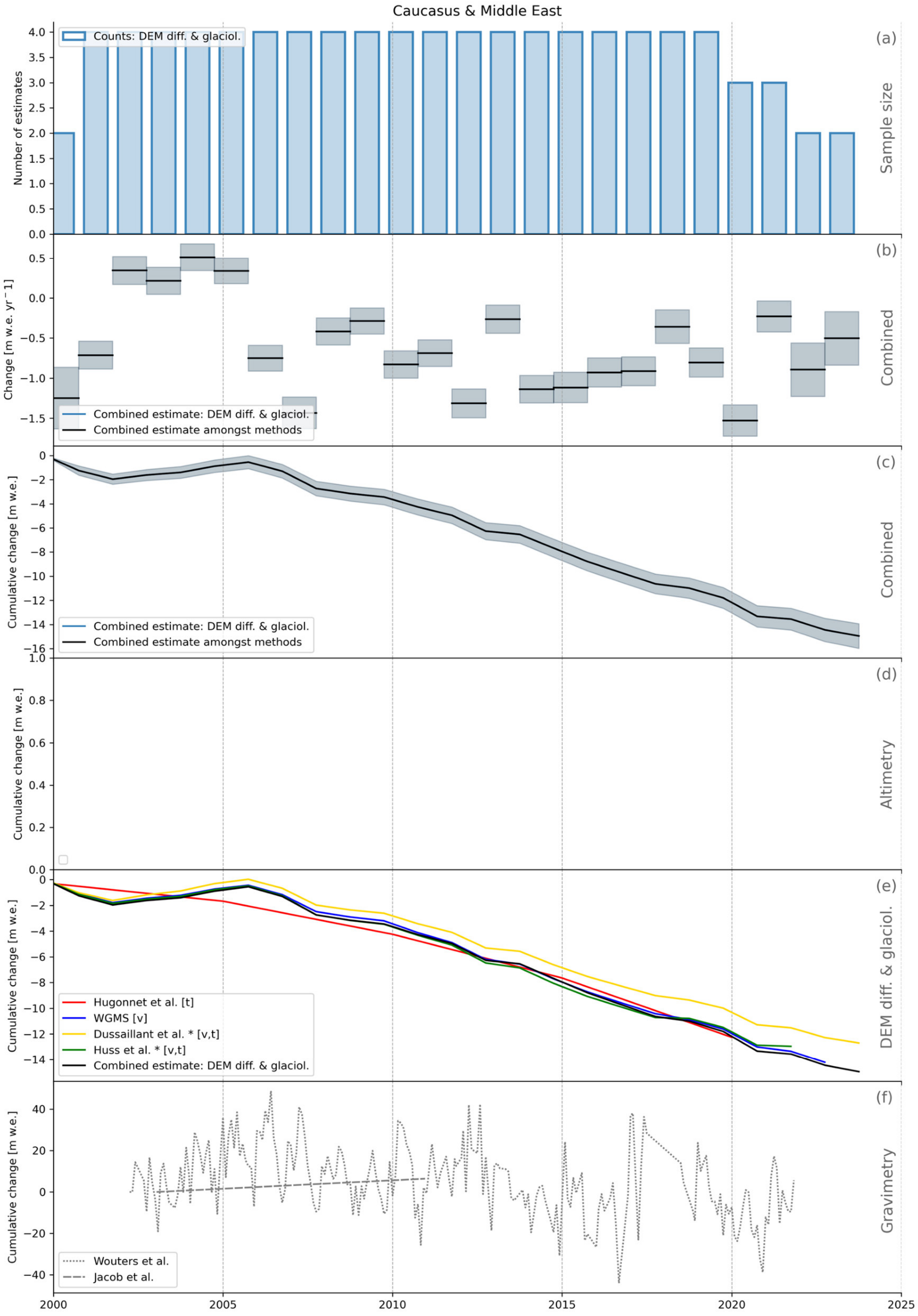

SI Figure 13 | Data submission for Central Asia

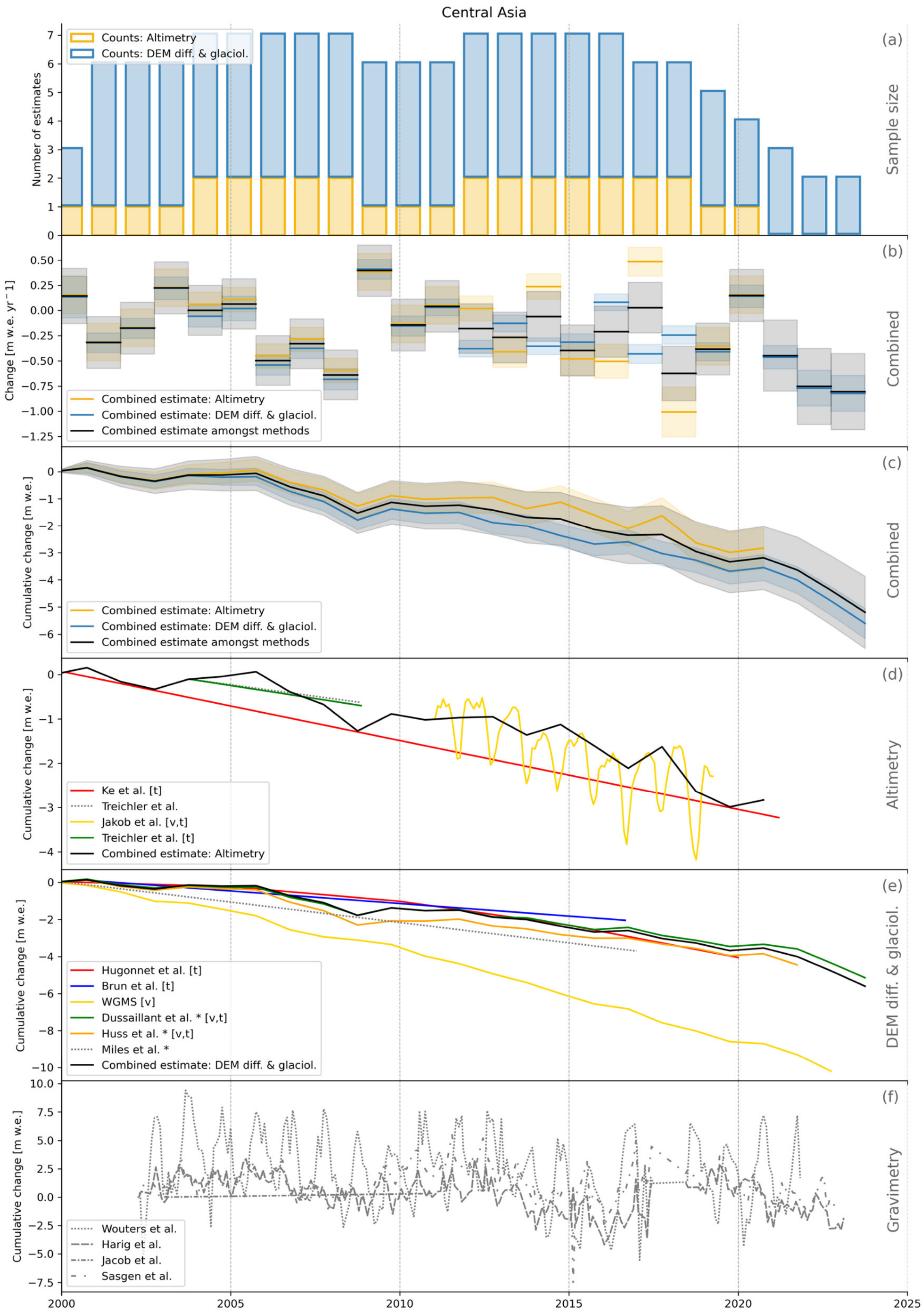

SI Figure 14 | Data submission for South Asia West

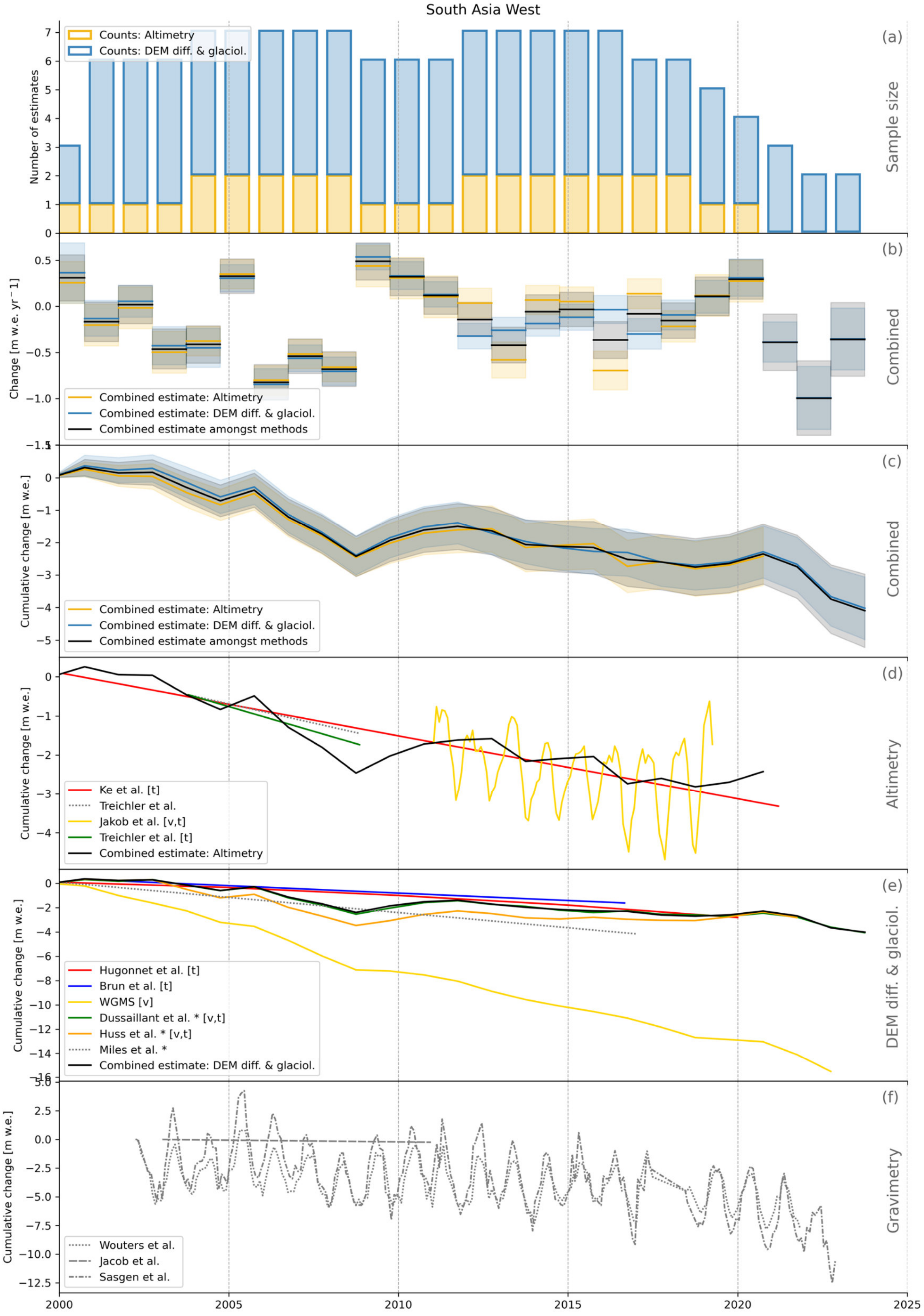

SI Figure 15 | Data submission for South Asia East

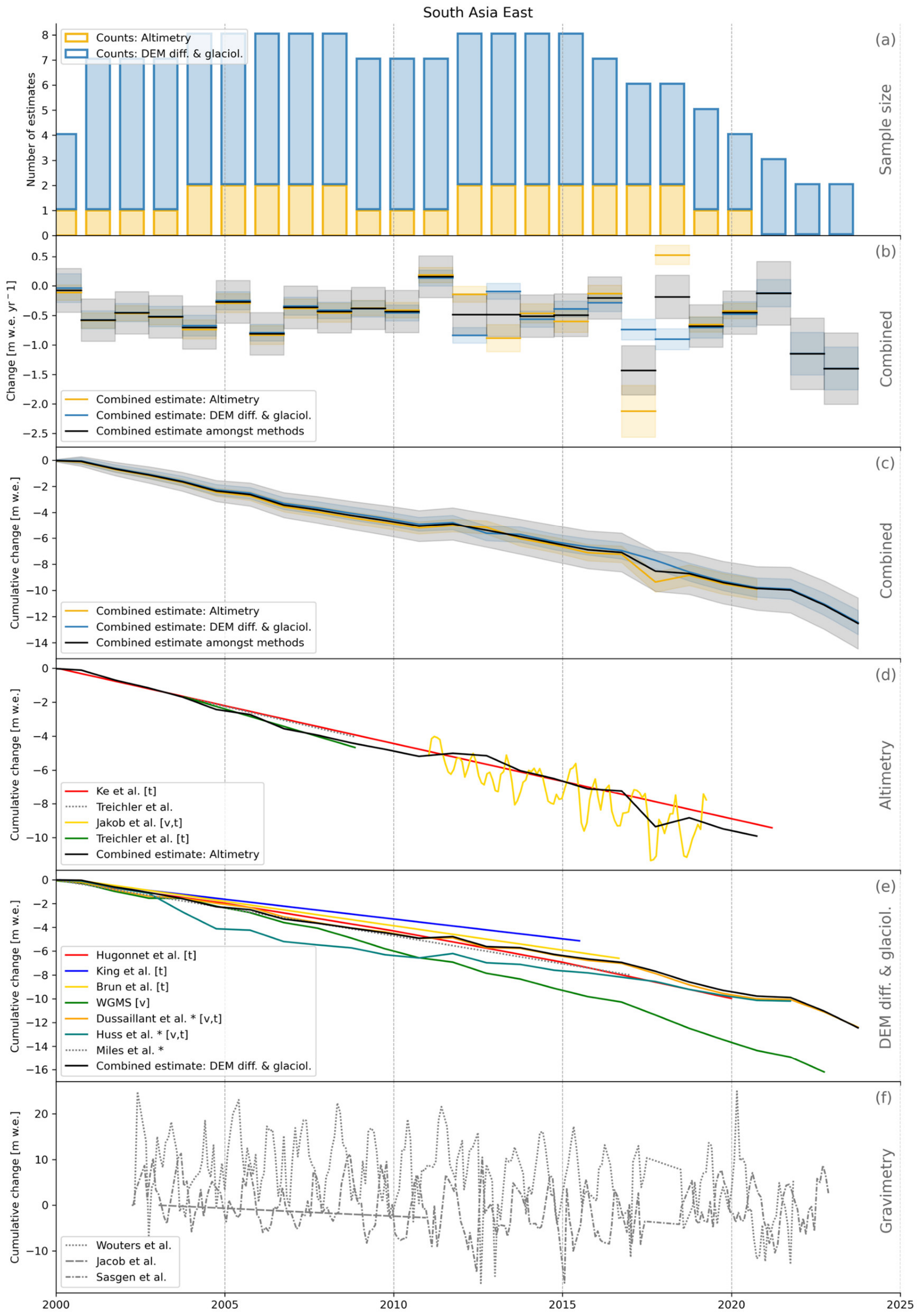

SI Figure 16 | Data submission for Low Latitudes

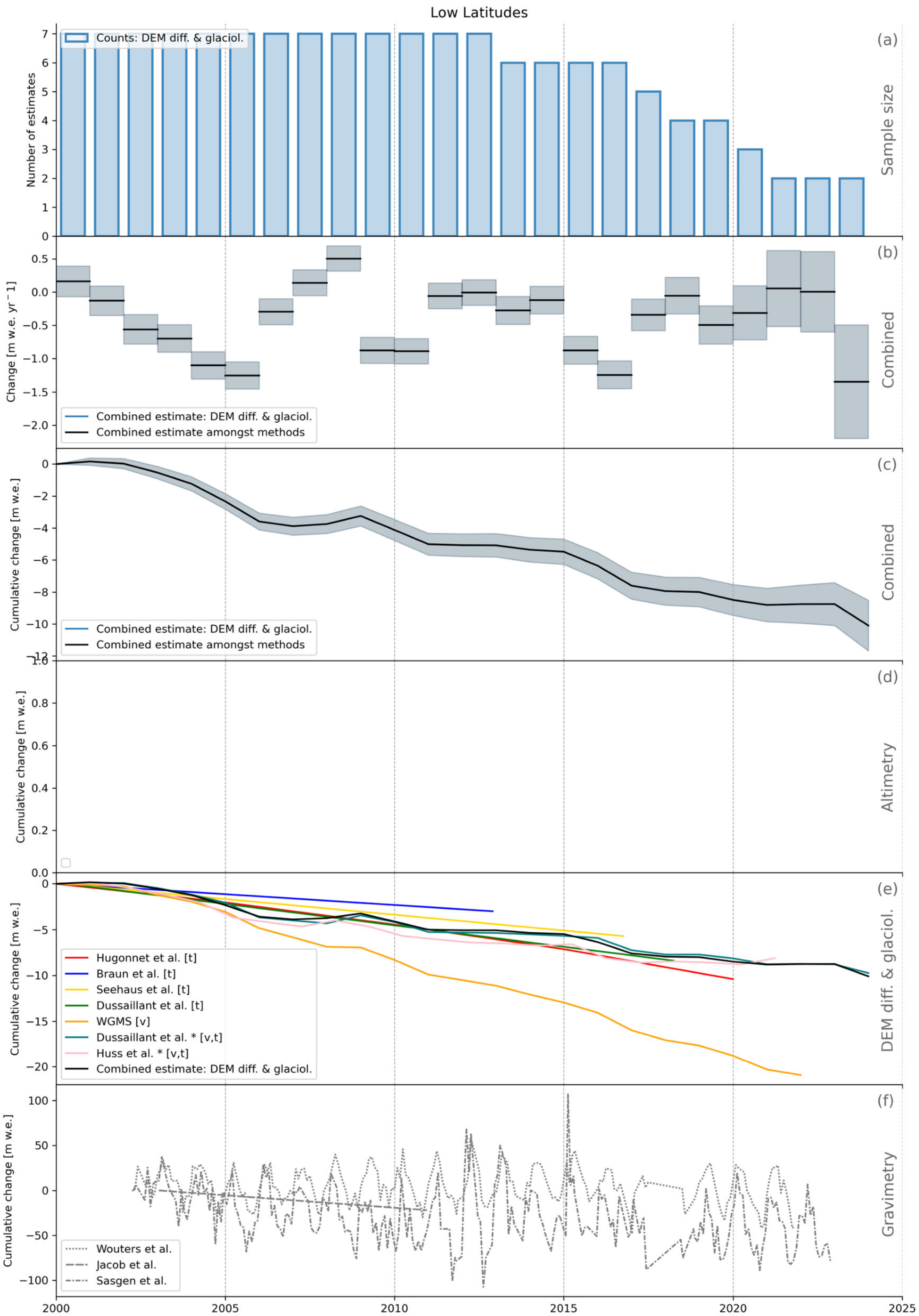

SI Figure 17 | Data submission for Southern Andes

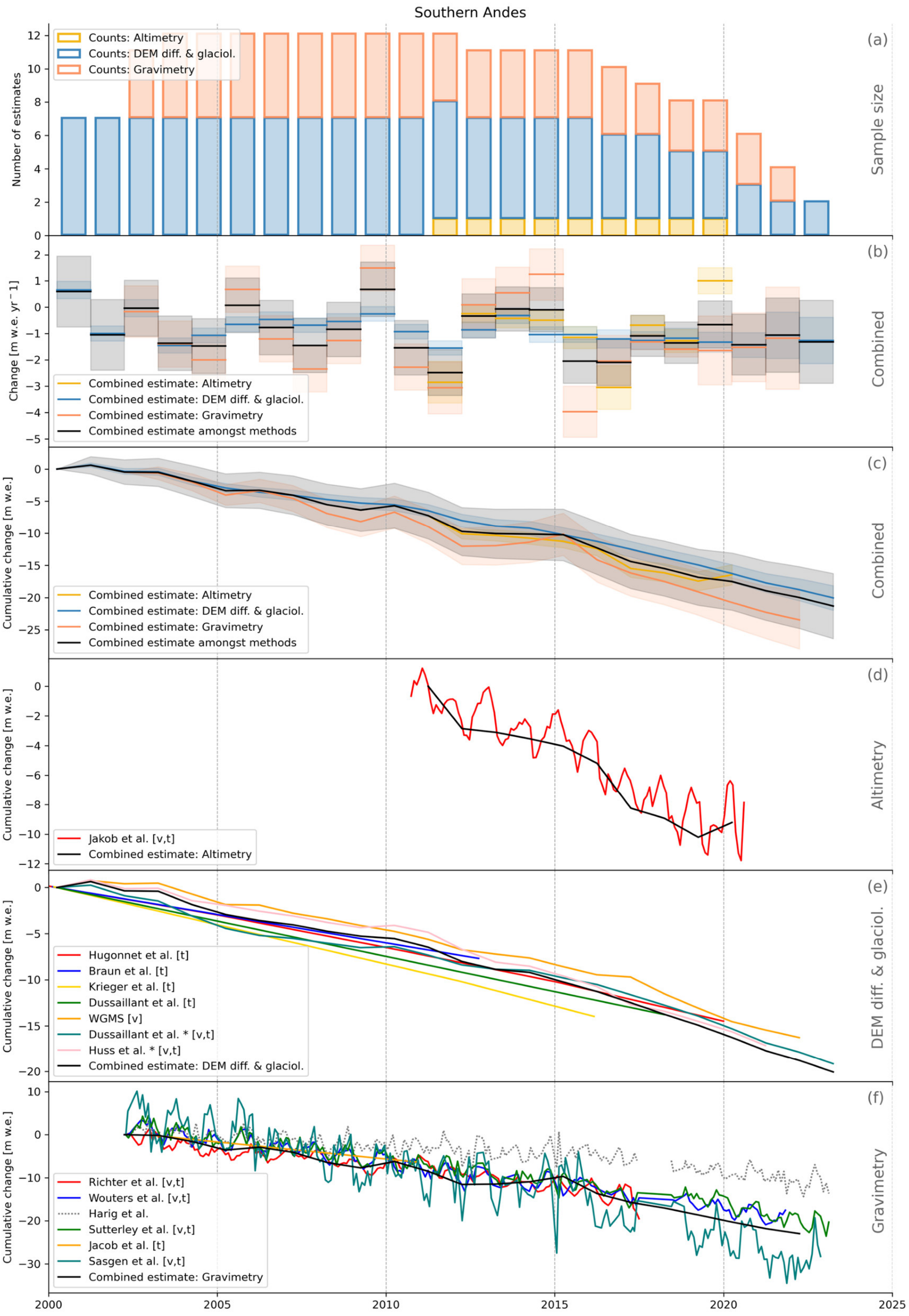

SI Figure 18 | Data submission for New Zealand

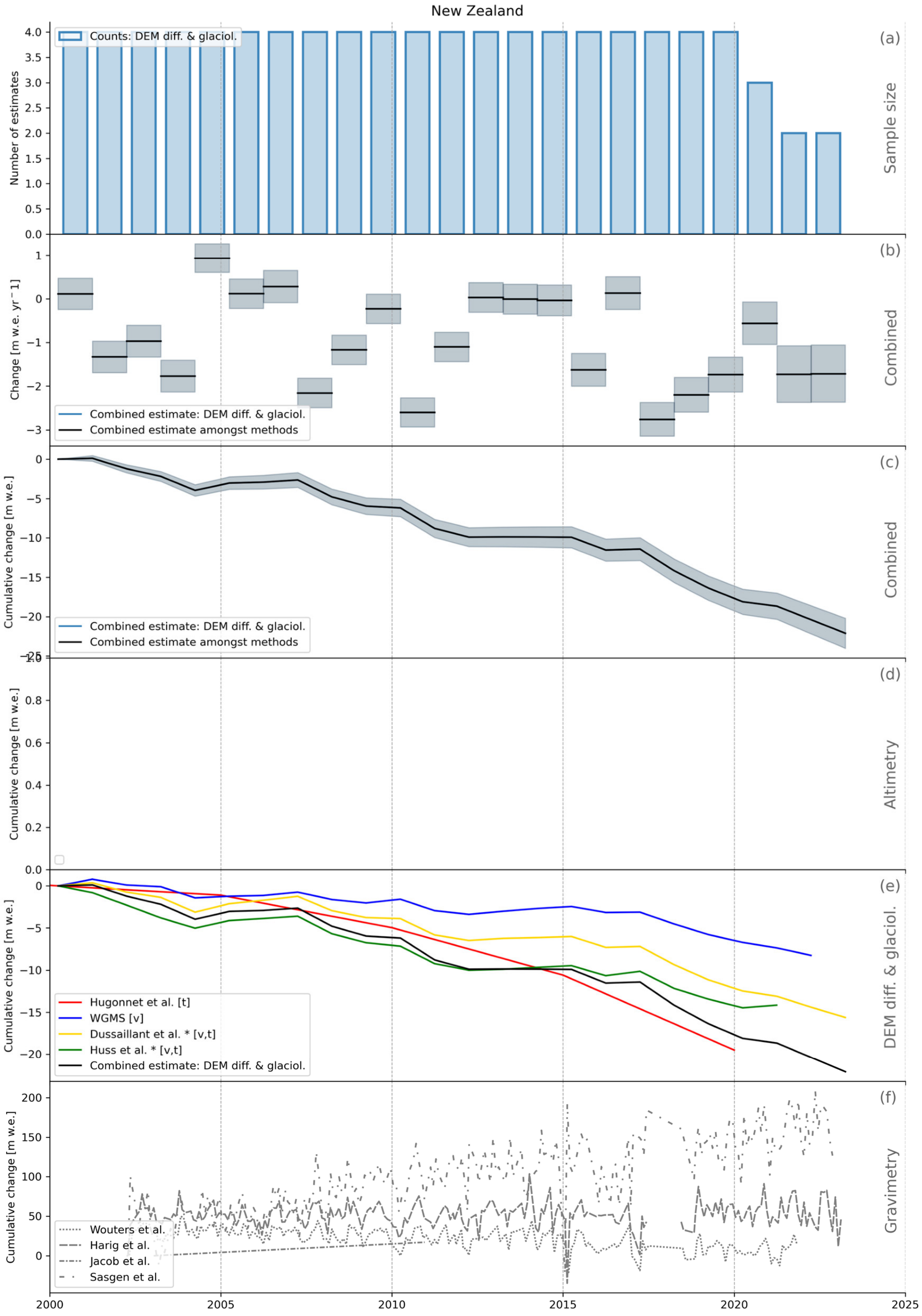

SI Figure 19 | Data submission for Antarctic and Subantarctic Islands

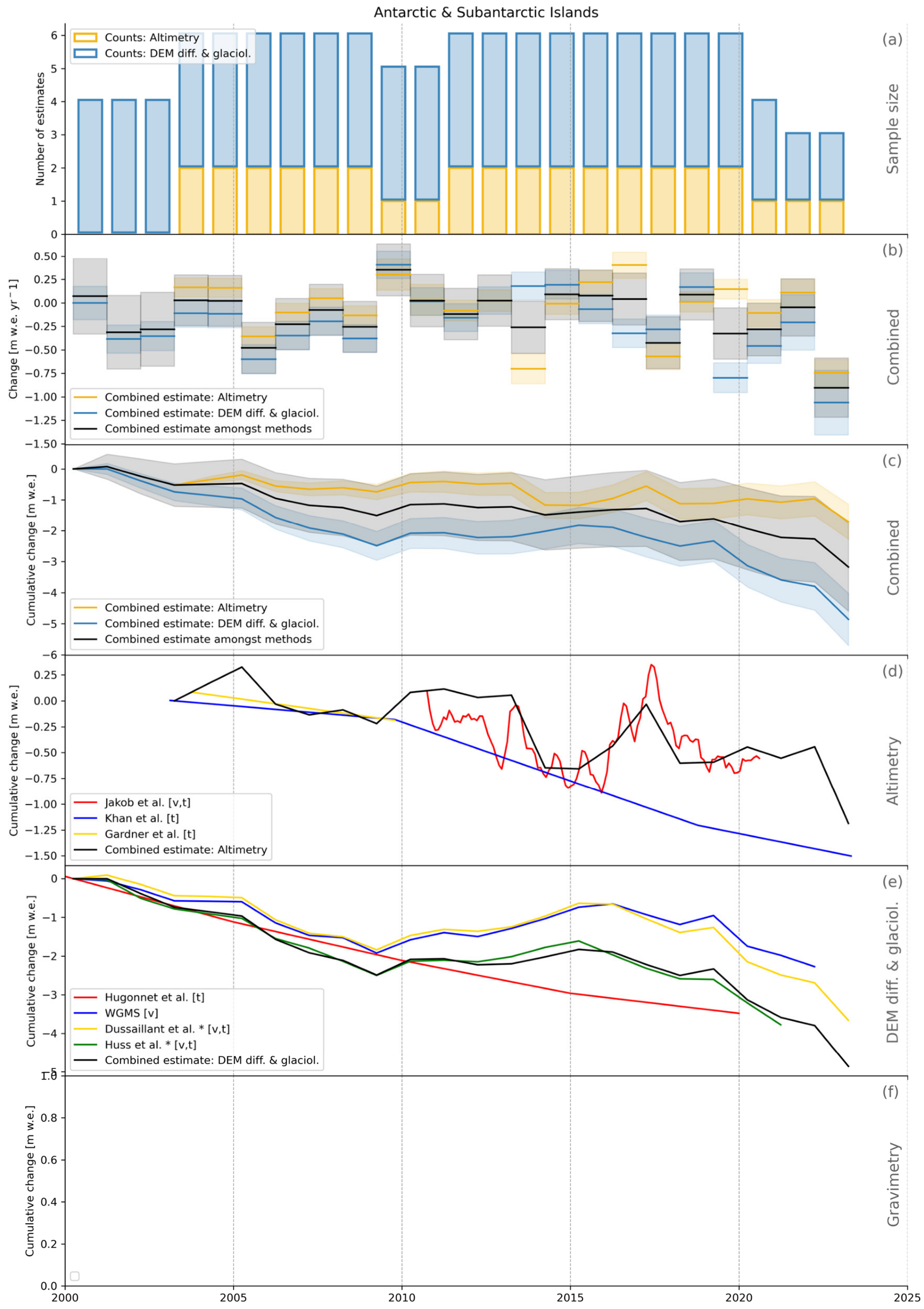

SI Figure 20 | Glacier mass-change observations and model projections (CMIP5). Regional comparison of observed cumulative glacier mass changes (in Gt) since 2000 with ensemble projections for 2007–2100 from the glacier model intercomparison project (GlacierMIP2, based on CMIP5) from Marzeion et al. (2020)[44], as used in IPCC AR6. Glacier mass-change observations (black line) are accompanied by their 95% confidence intervals (grey shading). For the projections, ensemble medians (blue and red lines) are shown with 95 percentile ranges (blue and red shadings) for low and high emission scenarios, respectively. Projections have been offset at their start date (2007) to fit the cumulative value of the observations.

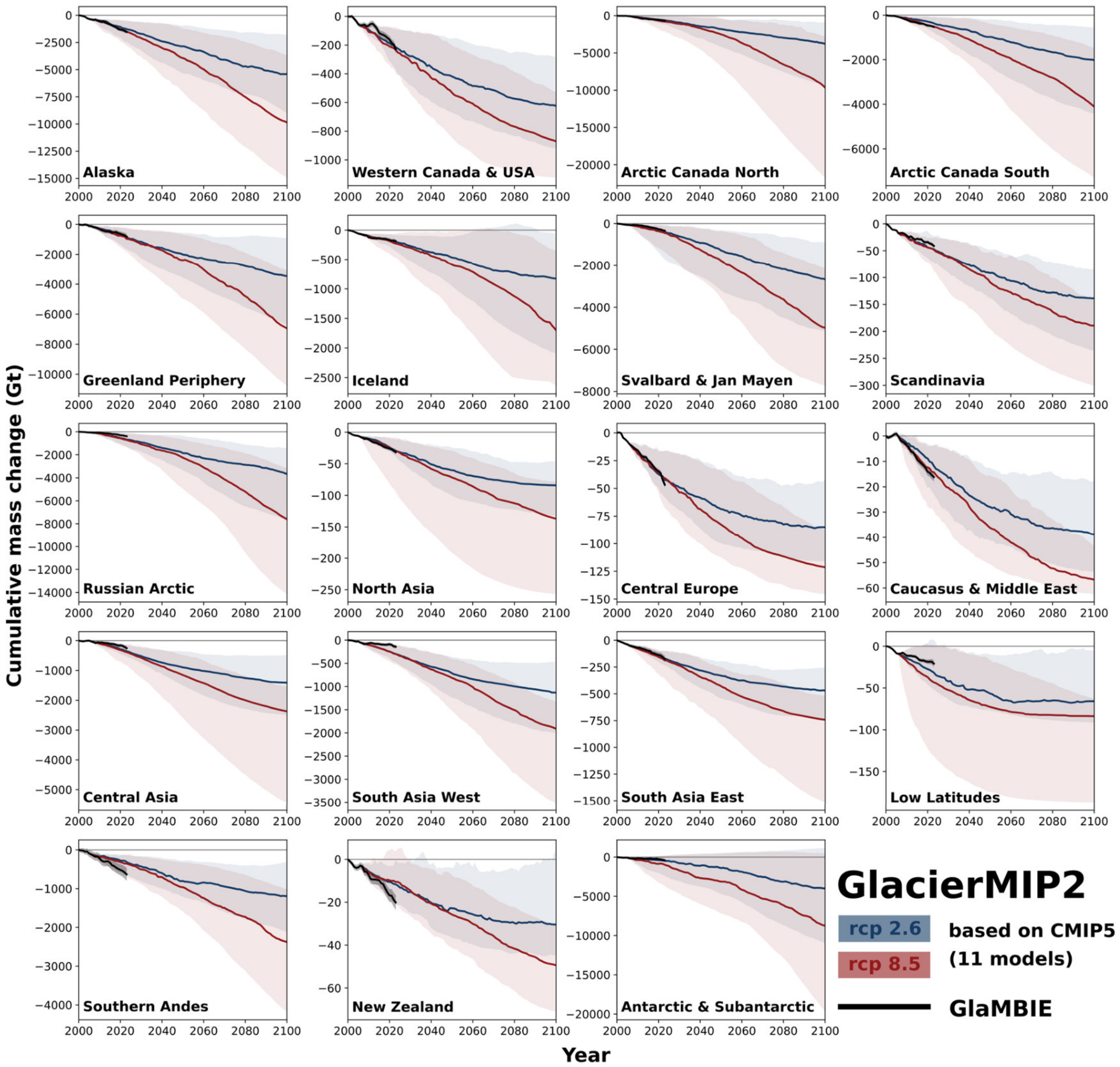

SI Figure 21 | Glacier mass-change observations and model projections (CMIP6). Regional comparison of observed cumulative glacier mass changes (in Gt) since 2000 with ensemble projections for 2007–2100 from a more recent model study (based on CMIP6) from Zekollari et al. (2024)[45]. Glacier mass-change observations (black line) are accompanied by their 95% confidence intervals (grey shading). For the projections, ensemble medians (blue and red lines) are shown with 90 percentile ranges (blue and red shadings) for low and high emission scenarios, respectively. Projections have been offset at their start date (2007) to fit the cumulative value of the observations.

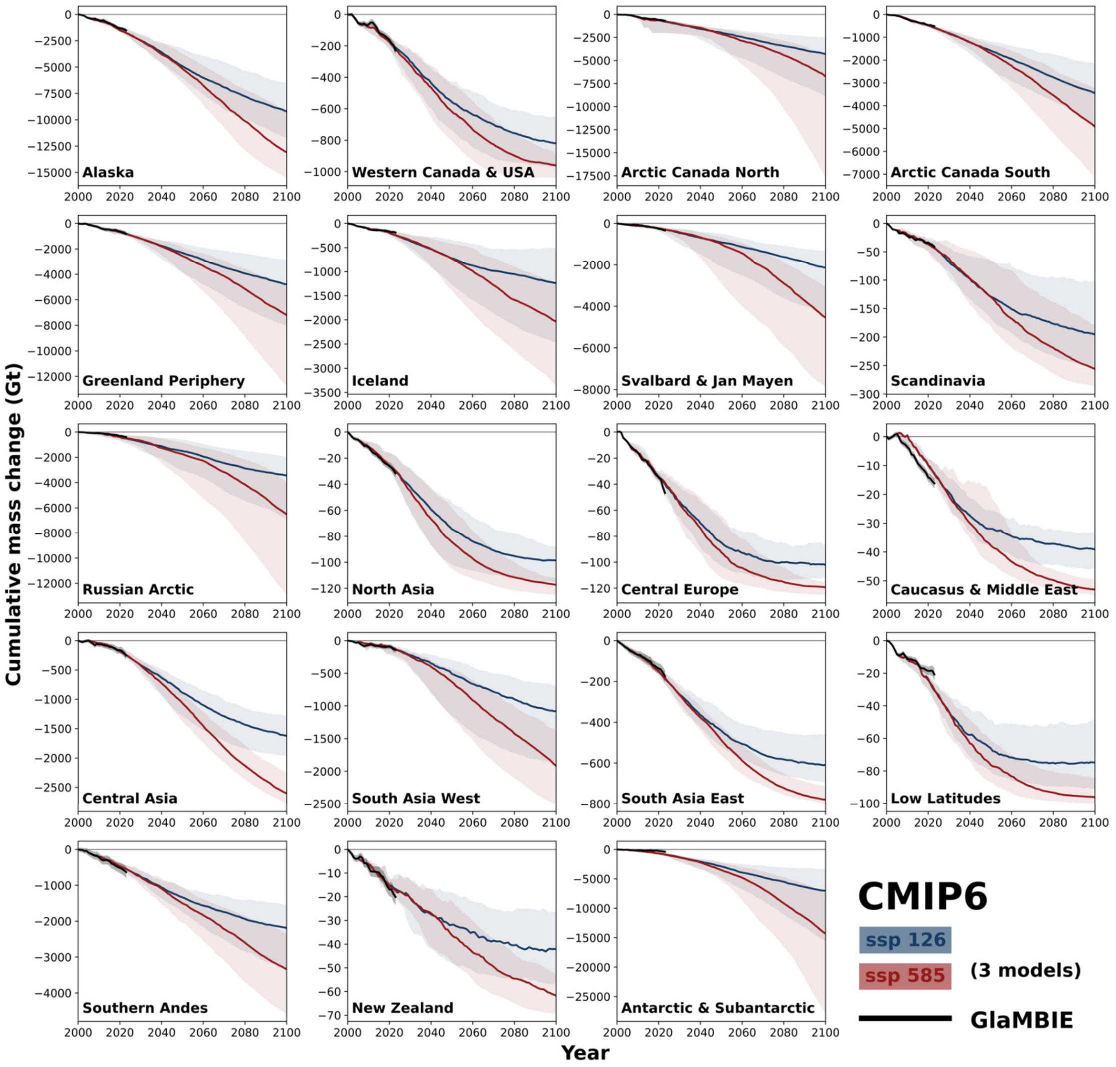

SI Figure 22 | Author contributions following the Contributor Role Taxonomy (CRediT) system by Allen et al. (2019)[46].

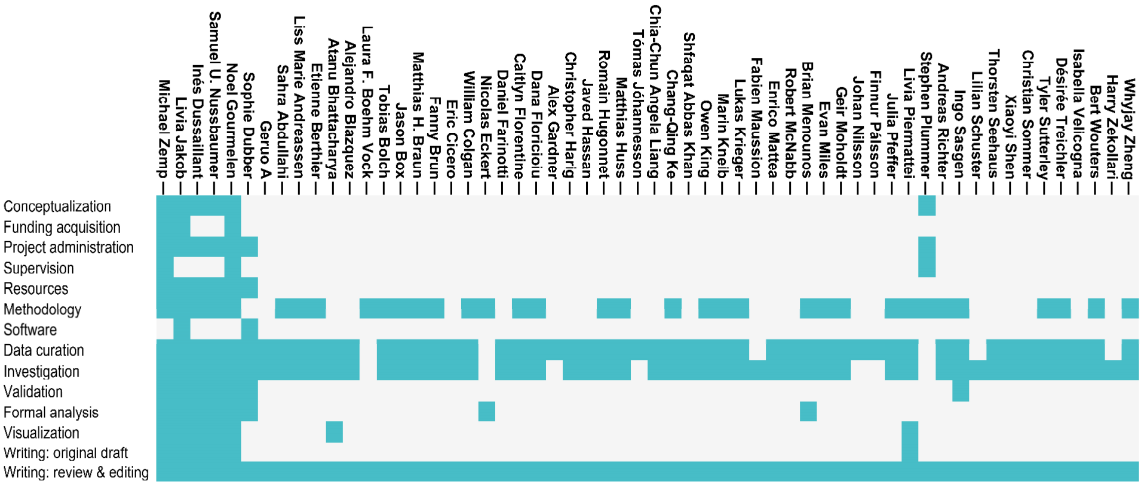

SI References

1. Bolch, T. *et al.* Mass loss of Greenland's glaciers and ice caps 2003–2008 revealed from ICESat laser altimetry data. *Geophysical Research Letters* **40**, 875–881 (2013).
2. Foresta, L. *et al.* Surface elevation change and mass balance of Icelandic ice caps derived from swath mode CryoSat-2 altimetry. *Geophysical Research Letters* **43**, (2016).
3. Gardner, A. S. *et al.* A Reconciled Estimate of Glacier Contributions to Sea Level Rise: 2003 to 2009. *Science* **340**, 852–857 (2013).
4. Jakob, L., Gourmelen, N., Ewart, M. & Plummer, S. Spatially and temporally resolved ice loss in High Mountain Asia and the Gulf of Alaska observed by CryoSat-2 swath altimetry between 2010 and 2019. *The Cryosphere* **15**, 1845–1862 (2021).
5. Jakob, L. & Gourmelen, N. Glacier Mass Loss Between 2010 and 2020 Dominated by Atmospheric Forcing. *Geophysical Research Letters* **50**, e2023GL102954 (2023).
6. Khan, S. A. *et al.* Accelerating Ice Loss From Peripheral Glaciers in North Greenland. *Geophysical Research Letters* **49**, e2022GL098915 (2022).
7. Menounos, B., Gardner, A., Florentine, C. & Fountain, A. Brief communication: Recent estimates of glacier mass loss for western North America from laser altimetry. *The Cryosphere* **18**, 889–894 (2024).
8. Nilsson, J., Gardner, A. S. & Paolo, F. S. Elevation change of the Antarctic Ice Sheet: 1985 to 2020. *Earth Syst. Sci. Data* **14**, 3573–3598 (2022).
9. Nilsson, J. & Gardner, A. S. Elevation Change of the Greenland Ice Sheet and its Peripheral Glaciers: 1992–2023. Preprint at https://doi.org/10.5194/essd-2024-311 (2024).
10. Tepes, P. *et al.* Changes in elevation and mass of Arctic glaciers and ice caps, 2010–2017. *Remote Sensing of Environment* **261**, 112481 (2021).
11. Treichler, D., Kääb, A., Salzmann, N. & Xu, C.-Y. Recent glacier and lake changes in High Mountain Asia and their relation to precipitation changes. *The Cryosphere* **13**, 2977–3005 (2019).
12. Abdullahi, S. *et al.* Estimating Penetration-Related X-Band InSAR Elevation Bias: A Study over the Greenland Ice Sheet. *Remote Sensing* **11**, 2903 (2019).
13. Wessel, B., Bertram, A., Gruber, A., Bemm, S. & Dech, S. A new high-resolution elevation model of Greenland derived from TanDEM-X. *ISPRS Ann. Photogramm. Remote Sens. Spatial Inf. Sci.* **III–7**, 9–16 (2016).
14. Andreassen, L. M., Elvehøy, H., Kjøllmoen, B. & Engeset, R. V. Reanalysis of long-term series of glaciological and geodetic mass balance for 10 Norwegian glaciers. *The Cryosphere* **10**, 535–552 (2016).
15. Braun, M. H. *et al.* Constraining glacier elevation and mass changes in South America. *Nature Clim Change* **9**, 130–136 (2019).
16. Brun, F., Berthier, E., Wagnon, P., Kääb, A. & Treichler, D. A spatially resolved estimate of High Mountain Asia glacier mass balances from 2000 to 2016. *Nature Geosci* **10**, 668–673 (2017).
17. Dussaillant, I. *et al.* Two decades of glacier mass loss along the Andes. *Nat. Geosci.* **12**, 802–808 (2019).
18. King, O., Bhattacharya, A., Bhambri, R. & Bolch, T. Glacial lakes exacerbate Himalayan glacier mass loss. *Sci Rep* **9**, 18145 (2019).
19. Abdel Jaber, W., Rott, H., Floricioiu, D., Wuite, J. & Miranda, N. Heterogeneous spatial and temporal pattern of surface elevation change and mass balance of the Patagonian ice fields between 2000 and 2016. *The Cryosphere* **13**, 2511–2535 (2019).

20. Piermattei, L. *et al.* Observing glacier elevation changes from spaceborne optical and radar sensors – an inter-comparison experiment using ASTER and TanDEM-X data. *The Cryosphere* **18**, 3195–3230 (2024).
21. Seehaus, T. *et al.* Changes of the tropical glaciers throughout Peru between 2000 and 2016 – mass balance and area fluctuations. *The Cryosphere* **13**, 2537–2556 (2019).
22. Seehaus, T. *et al.* Mass balance and area changes of glaciers in the Cordillera Real and Tres Cruces, Bolivia, between 2000 and 2016. *J. Glaciol.* **66**, 124–136 (2020).
23. Sommer, C. *et al.* Rapid glacier retreat and downwasting throughout the European Alps in the early 21st century. *Nat Commun* **11**, 3209 (2020).
24. Sommer, C., Seehaus, T., Glazovsky, A. & Braun, M. H. Brief communication: Increased glacier mass loss in the Russian High Arctic (2010–2017). *The Cryosphere* **16**, 35–42 (2022).
25. Zheng, W. *et al.* Accelerating glacier mass loss on Franz Josef Land, Russian Arctic. *Remote Sensing of Environment* **211**, 357–375 (2018).
26. WGMS. *Global Glacier Change Bulletin No. 5 (2020–2021)*. (ISC(WDS)/IUGG(IACS)/UNEP/UNESCO/WMO, World Glacier Monitoring Service, publication based on database version: doi:10.5904/wgms-fog-2023-09, Zurich, Switzerland, 2023).
27. Harig, C. & Simons, F. J. Ice mass loss in Greenland, the Gulf of Alaska, and the Canadian Archipelago: Seasonal cycles and decadal trends. *Geophysical Research Letters* **43**, 3150–3159 (2016).
28. Beveridge, A. K., Harig, C. & Simons, F. J. The changing mass of glaciers on the Tibetan Plateau, 2002–2016, using time-variable gravity from the GRACE satellite mission. *Journal of Geodetic Science* **8**, 83–97 (2018).
29. Jacob, T., Wahr, J., Pfeffer, W. T. & Swenson, S. Recent contributions of glaciers and ice caps to sea level rise. *Nature* **482**, 514–518 (2012).
30. Blazquez, A. *et al.* Exploring the uncertainty in GRACE estimates of the mass redistributions at the Earth surface: implications for the global water and sea level budgets. *Geophysical Journal International* **215**, 415–430 (2018).
31. Richter, A. *et al.* The Rapid and Steady Mass Loss of the Patagonian Icefields throughout the GRACE Era: 2002–2017. *Remote Sensing* **11**, 909 (2019).
32. Sasgen, I. *et al.* Arctic glaciers record wavier circumpolar winds. *Nat. Clim. Chang.* **12**, 249–255 (2022).
33. Sutterley, T. C. & Velicogna, I. Improved Estimates of Geocenter Variability from Time-Variable Gravity and Ocean Model Outputs. *Remote Sensing* **11**, 2108 (2019).
34. Sutterley, T. C., Velicogna, I. & Hsu, C. Self-Consistent Ice Mass Balance and Regional Sea Level From Time-Variable Gravity. *Earth and Space Science* **7**, e2019EA000860 (2020).
35. Wouters, B., Gardner, A. S. & Moholdt, G. Global Glacier Mass Loss During the GRACE Satellite Mission (2002-2016). *Front. Earth Sci.* **7**, 96 (2019).
36. Box, J. E. *et al.* Global sea-level contribution from Arctic land ice: 1971–2017. *Environ. Res. Lett.* **13**, 125012 (2018).
37. Colgan, W. *et al.* Hybrid glacier Inventory, Gravimetry and Altimetry (HIGA) mass balance product for Greenland and the Canadian Arctic. *Remote Sensing of Environment* **168**, 24–39 (2015).
38. Dussaillant, I., Bannwart, J., Paul, F. & Zemp, M. Glacier mass change gridded data from 1976 to present derived from the Fluctuations of Glaciers Database. World Glacier Monitoring Service, Copernicus Climate Change Service (C3S) Climate Data Store (CDS) https://doi.org/10.24381/CDS.BA597449 (2023).

39. Dussaillant, I. *et al.* Annual mass changes for each glacier in the world from 1976 to 2023. Preprint at https://doi.org/10.5194/essd-2024-323 (2024).

40. Zemp, M. Glacier monitoring tracks progress in limiting climate change. *Nature* **576**, 39–39 (2019).

41. Fan, Y. *et al.* Glacier mass-balance estimates over High Mountain Asia from 2000 to 2021 based on ICESat-2 and NASADEM. *J. Glaciol.* **69**, 500–512 (2023).

42. Miles, E. *et al.* Health and sustainability of glaciers in High Mountain Asia. *Nat Commun* **12**, 2868 (2021).

43. Aðalgeirsdóttir, G. *et al.* Glacier Changes in Iceland From ∼1890 to 2019. *Front. Earth Sci.* **8**, 523646 (2020).

44. Marzeion, B. *et al.* Partitioning the Uncertainty of Ensemble Projections of Global Glacier Mass Change. *Earth's Future* **8**, e2019EF001470 (2020).

45. Zekollari, H. *et al.* Twenty-first century global glacier evolution under CMIP6 scenarios and the role of glacier-specific observations. *The Cryosphere* **18**, 5045–5066, (2024).

46. Allen, L., O'Connell, A. & Kiermer, V. How can we ensure visibility and diversity in research contributions? How the Contributor Role Taxonomy (CRediT) is helping the shift from authorship to contributorship. *Learned Publishing* **32**, 71–74 (2019).
