# Supplementary material for: Community estimate of global glacier mass changes from 2000 to 2023
Source: Nature. 2025 Feb 19;639(8054):382–8. doi: 10.1038/s41586-024-08545-z (PMC11903323; doi:10.1038/s41586-024-08545-z)
Supplement: Supplementary file 2 — Peer Review File [file 41586_2024_8545_MOESM2_ESM.pdf]

nature portfolio

Peer Review File

Community estimate of global glacier mass changes from 2000 to 2023

Corresponding Author: Professor Michael Zemp

This file contains all reviewer reports in order by version, followed by all author rebuttals in order by version.

Attachments originally included by the reviewers as part of their assessment can be found at the end of this file.

Version 0:

Reviewer comments:

Referee #1

(Remarks to the Author)
Over the past two decades, a plethora of research has concentrated on examining the mass balance of glaciers within specific regions. These studies have consistently reported a sustained and, in some cases, an accelerated rate of glacier mass loss across the globe. Nevertheless, a limited number of investigations have attempted to assess the mass balance of glaciers on a global scale. Furthermore, the existing studies in this domain have predominantly relied on a singular data methodology. The article introduces innovative aspects in assessing global glacier mass changes through the Glacier Mass Balance Intercomparison Exercise (GlaMBIE), which includes a comprehensive inter-comparison across various observation methods, robust data homogenization processes, a novel five-step methodology for data combination, detailed uncertainty quantification, and the creation of a new observational baseline.
The research presented in this article is highly laudable for its methodological rigor and significant contributions to the field. The academic community concerned with glacier mass loss has directed significant attention towards understanding the glaciers' response to climate change and the consequential impacts of these changes on sea-level dynamics. To the best of my knowledge, no prior research has endeavored to quantify the global glacier mass balance employing a diverse array of observational techniques and datasets. This work built a new method for data homogenization and combination and, in doing so, will redirect future effects of the glacier mass balance community. The quality of both the experimental approach and treatment of uncertainty is excellent and the writing style is quite clear. The figures included in the main text are visually appealing. The references are appropriate and complete.

I have one minor concerns with the work at present, though both should be readily addressed with some simple clarifications, discussion of assumptions, and a supplementary figure.

My primary concern is the glacier mass in 2000. To account for glacier shrinkage, the authors used regional glacier area-change rates compiled from IPCC AR5 and corrected glacier area from RGI 6.0 to the year 2000 and computed yearly time series of regional glacier area. Regional glacier volume and mass area from the Ice Thickness Models Intercomparison eXperiment (ITMIX). Given that the author has corrected glacier area from RGI 6.0 to the year 2000, it is imperative that the glacier volume and mass estimates be similarly adjusted. This is essential as the ITMIX dataset does not accurately reflect the volume and mass of glaciers as of the year 2000. The authors should pay more attention to clarify it.

Minor line-by-line comments

Line 702, the reference 74 had been published in The Cryosphere, it could be revised the cite.

--Kunpeng Wu

Referee #2

(Remarks to the Author)
Please see the formal review.

Referee #3

(Remarks to the Author)
A. Summary of the key results

The paper presents the findings of the GLAMBIE project (https://glambie.org/project-team/) – a community estimate of glacier mass change for the period from 2000 to 2023. The results have been generated following extensive community input, engagement, checking and homogenisation using data from a range of remote sensing approaches (DEM differencing, altimetry, gravimetry, hybrid methods) and direct mass balance observations (from a smaller subset of global glaciers). Noting that this work represents a huge and praiseworthy community effort, the overall results are not (within uncertainty) significantly different from the 6th IPCC assessment report or previous global studies. Given the uncertainties, it is also not clear that this new estimate is indeed more accurate than previous work.

Results are calculated at the regional rather than at glacier scale which leads to some limitations. For example, the authors state in the abstract that 'Our results provide a new observational baseline for calibrating model ensembles'. However, most model calibration occurs at glacier scale. E.g. in glacier models (e.g., GloGEM, OGGM, PyGEM and so on), each glacier is simulated separately, and the surface mass balance parameters are also calibrated at single glacier scale. This widely accepted approach ensures the accuracy of global projections. Thus, a global glacier mass change dataset like Hugonnet et al. (2021; https://www.nature.com/articles/s41586-021-03436-z) is more readily useful for model calibration.

B. Originality and significance: if not novel, please include reference

The results are original but a current preprint (Dussaillant et al. (2024) https://essd.copernicus.org/preprints/essd-2024-323/) shares many of the authors and a somewhat similar approach – a blending of glacier mass balance observations with remotely sensed surface elevation datasets. However, in Dussaillant et al. (2024), mass changes are resolved at individual glacier scale (which I believe is an improvement). What are the relationships and dependencies between these two papers and datasets?

The overall findings at global scale (glacier mass loss of 273±16 gigatonnes per year from 2000 to 2023) reported in this submission are very similar (-267 ± 16 gigatonnes per year in 2000-2019) to those reported by Hugonnet et al., 2021. The years do not exactly overlap and the authors point out the real difference is 16%, but given the absolute uncertainties are relatively large (at face value between 5-10%, and possibly larger due to processes not considered) the updated mass loss estimate is not a hugely new finding for a general readership. The identified acceleration in mass loss over the last decade was also reported by Hugonnet et al., 2021.

For example, on Line 196 - 'Our results generally confirm glacier mass-loss trends reported by the latest IPCC reports.'

The results in this paper are certainly more comprehensive but the basic pattern and magnitude of mass loss is similar to previous work.

The findings are extremely important. However I question whether they are novel.

C. Data & methodology: validity of approach, quality of data, quality of presentation

My impression is that in the new dataset, glaciological observations dominate the annual variability and DEM differencing dominates the long-term trend. P11 L472~L474 suggests this - "In cases without annual observations (e.g., altimetry pre-2010 or gravimetry during the observational gap between GRACE and GRACE-FO), we used the averaged anomaly from the other observation methods to fill in βy for missing years.' This may be why the overall trends are very similar to Hugonnet et al. (2021) and Dussaillant et al. (2024).

This leads me to a question about the annual variability. Is extrapolating individual mass balance observations to regional scale ever valid? Let me explain in more detail.
- Glacier mass balance measurements are unique to each glacier.
- Mass balance programmes are nearly always carried out on glaciers that (for example) are lower in elevation, flatter and more accessible and which may very well not reflect the region-wide variability.
- The strong spatial correlation between glacier mass balance over several hundred kilometres doesn't mean that individual glaciers have a similar mass change to the aggregated trend in each RGI region.

It's easy to understand how to extract regional values based on DEM differencing, altimetry and gravimetry. However, how to calculate the regional value from a few and unevenly distributed glaciological observations remains unclear. I would like the authors to explain this further.

Related to this (top of Page 5): Given that glaciological measurements are shown to have a bias, do the authors have any recommendations on the future of direct mass balance programmes?

Further and more specific comments:

Page 3 – 'However, mass loss due to calving front retreat of marine and lake-terminating glaciers is not included since that does not significantly impact global sea level.'

Comment: this is true if ice is floating (for the component of the glacier above floatation) but many such lake and fiord glaciers are grounded or partially grounded, and I am not sure this statement is correct. Do the authors have evidence to support it? What proportion of global glaciers are in this category? I note the largest mass loss reported in this study is from Greenland, Alaska and Patagonia where calving glaciers are common.

Page 3 – Line 132. 'This increase is concurrent with the general pattern of atmospheric warming, overlaid by regional decadal variability9,26,49'.
- The authors need to make it clear that the examples provided below this sentence (e.g. end of Karakoram-Kunlun anomaly, slowdown of Icelandic mass loss) follow on from this sentence.
- I'd like to see further discussion of these regional results. Methodological differences etc (the next section) are important for the glaciological community but are of less general interest.

D. Appropriate use of statistics and treatment of uncertainties
The quality of the data processing and presentation is excellent I very much appreciate the community vetting process.

Note that P1 L27: "32±8 mm" should be "320±80 mm" or 32 cm.

E. Conclusions: robustness, validity, reliability
I believe that the conclusions of this paper are robust at the broad scale. However, I'm not sure that they offer sufficient novelty compared to previous work or have sufficiently reduced uncertainty for them to become a new benchmark.

F. Suggested improvements: experiments, data for possible revision
It might be more convincing to compile different glacier mass change datasets at single glacier scale and then to regional scale rather than based on 19 first-order glacier regions directly.

G. References: appropriate credit to previous work?
No issues

Clarity and context: lucidity of abstract/summary, appropriateness of abstract, introduction and conclusions

Abstract: Note that glacier retreat also impacts biodiversity and not just sea level, hazards and water resources. The authors should cite https://www.nature.com/articles/s41586-024-07778-2

The paper is generally well written but would benefit from English language editing.

See further minor comments below:
Page 1 - Line 22 – use non gendered language.
Page 2 – Line 67. Does this mean the 7th IPCC Assessment report. Please specify.
Page 2 – Line 76. Change 'and cover periods since 2000' to 'and covers the time period since the early 2000s'.

For regional glacier area and related changes over time, we assume a general uncertainty of ±5%68.
-What is the justification for this?

How is the influence of changing glacier area (as glaciers retreat) on mass balance considered? Or does this not matter because of the way that mass balance observations influence variability rather than trends?

(Remarks on code availability)

Version 1:

Reviewer comments:

Referee #2

(Remarks to the Author)
The authors have apparently made substantial effort trying to improve the manuscript and clarify reviewers' comments and questions. Even though my main concerns remain more or less the same (the novelty of the conclusions and the suitability of such a comprehensive and complicated analysis to the broad general audience of Nature), I will trust and support the editors' decision.

Here are a few comments that the authors may consider to further improve the manuscript.

1) Extended Data Figure 1 is still too busy and very confusing. I didn't see much difference compared to the original version.

2) Supplementary Data Table 1 can be better organized. A whole page is occupied by the affiliation information of the WGMS team. There is no need to provide all the information here in the table (providing a reference is enough) and break the table into two separate parts.

3) Time spans of different inputs from different groups for the same technique should be also given here in Supplementary Data Table 1.

4) Some of the inputs only cover a short period of time (e.g., Gravimetry from Jacob et al., 2003-2011 and Colgan et al., 2002-2009). It would be more helpful to get some updated series. If the authors only want to get an ensemble average of previously published series (regardless the time spans), there should be many more other published series available.

5) The authors may want to use consistent colors/formats for the Supplementary Data Figures. Some of the bottom panels (Gravimetry) are in b/w, while most of others are in color.

(Remarks on code availability)

Referee #3

(Remarks to the Author)
The authors have done a thorough job at responding to reviewer and editorial feedback, and the new sections and figures improve the paper. The new text (from lines 83 -92) is certainly much clearer.

I still don't believe that the regional estimates will be useful for model evaluation (given that this is mostly done at glacier scale), but the community approach is noteworthy and reproducible, and results are important for understanding regional mass changes.

However, in re-reading the entire revised manuscript I have become concerned about the description of uncertainties and interpretation of results considering the uncertainty bounds. Even though this seems to be more of an issue of presentation than substance, I'd like it to be corrected before the paper can be considered for publication.

Starting with the revised abstract -

While our results generally confirm the findings from previous assessments at a global scale, we found some large regional differences, partly within random errors, suggesting a systematic error among observation methods.

- as well as being difficult to read ('partly within random errors'), this sentence is concerning because differences within uncertainty bounds cannot be interpreted as meaningful.

Looking at this further within the manuscript -

Line 225. At a regional level, the differences to the results from AR6 range between −20% for the Southern Andes and +53% for the Antarctic and Subantarctic Islands AND are often within uncertainties (Extended Data Table 3).

AND needs to be replaced with BUT in this sentence. The fact that the differences are within uncertainties limits what the authors can interpret about these findings.

From Line 225 -
'GlaMBIE extends the temporal coverage to 2023, provides results at annual resolution, and has established a community mechanism to allow estimates to be updated towards IPCC AR7. Regarding uncertainties, our estimates come with error bars that are smaller than the ones reported in IPCC SROCC which the improvement in observational coverage can explain. Compared to IPCC AR5 and AR6, our estimates feature similar uncertainties over multi-year periods but additionally have the advantage of providing results and uncertainties at annual resolution.'

Although it is true that the uncertainties are smaller, the authors also state earlier in the paper that 'Since true independence among these mass-change estimates is unlikely, our random errors will likely represent a lower range of the actual uncertainty for a given region.'

All of these statements need to be reconciled to avoid giving the impression of overinterpretation.

Perhaps the authors can just highlight areas where there are very large differences from previous assessments? For example, the results for peripheral glaciers in Antarctica are very different to previous estimates and this is noteworthy and could receive more attention given that it seems to be for good reason (inclusion of altimetry results).

Minor suggestions:

Could this sentence which interrupts the flow of the paragraph -

225. ‘Reference data at the glacier scale, such as from airborne laser surveys, are less prone to errors than space-borne observations and can be used for validation.’

be removed or added at the end of this paragraph using ‘For example, …’?

line 307. ‘gas emissions and associated warming to limit the impacts of glacier vanishing down-wasting on local geohazards, regional freshwater availability, and global sea-level rise.’

- Glacier downwasting implies only vertical change whereas glaciers are thinning and retreating. Suggest replacing ‘ glacier down wasting’ with ‘ glacier wastage’.

Open Access This Peer Review File is licensed under a Creative Commons Attribution 4.0 International License, which permits use, sharing, adaptation, distribution and reproduction in any medium or format, as long as you give appropriate credit to the original author(s) and the source, provide a link to the Creative Commons license, and indicate if changes were made.
In cases where reviewers are anonymous, credit should be given to 'Anonymous Referee' and the source.
The images or other third party material in this Peer Review File are included in the article’s Creative Commons license, unless indicated otherwise in a credit line to the material. If material is not included in the article’s Creative Commons license and your intended use is not permitted by statutory regulation or exceeds the permitted use, you will need to obtain permission directly from the copyright holder.
To view a copy of this license, visit https://creativecommons.org/licenses/by/4.0/

Community estimate of global glacier mass changes from 2000 to 2023

The GlaMBIE Team

Corresponding author: Michael Zemp, michael.zemp@geo.uzh.ch

Author's response related to the comments received to the manuscript 2024-07-14479

We thank the three referees and the editor for their feedback on our manuscript. We revised our manuscript after considering all their comments and suggestions, which helped improve our paper.

The main changes we made to the manuscript are as follows:

- A revised summary paragraph and main text to better highlight the progress of our paper regarding earlier findings.
- A revised main text to better explain our rationale for an intercomparison at a regional scale, emphasise the advance of GlaMBIE results relative to previous research, and highlight implications for future research.
- Two additional paragraphs in the main text to discuss the limitations and future perspectives of uncertainty estimates.
- A revised introduction paragraph to the Methods section to explain our approach in plain language and to provide references to the full details available in the Extended Data, in the Supplement Material, and in the open data and code repositories.
- A revised Extended Data Figure 1 for improved readability of the Sankey diagram showing all input data from research groups to sensor/products, observations methods, and glacier regions.
- A revised Extended Data Figure 3 for improved readability of differences among observation methods.
- A revised Extended Data Table 3 providing regional differences between our estimates and those reported in IPCC AR6.

Point-by-point reply to reviewer comments: editor and reviewer comments are in black, and the author's responses are in blue, with citations from the revised manuscript in green.

+ +

Comments by Editor, 27th August 2024

27th August 2024

Dear Michael

Your manuscript, "Community estimate of global glacier mass changes from 2000 to 2023", has now been seen by three referees, whose comments are attached below and as a PDF. While they find your work of interest, they have raised points that need to be addressed before we can make a decision on publication. We will need to consider your response to these concerns in the form of a revised manuscript accompanied by a list to explain your revisions. You will also need to

make some editorial changes to your paper so that it is as brief as possible and complies with our Guide to Authors (https://www.nature.com/nature/for-authors).

Response: We revised our manuscript addressing all points. We provided a new version, which includes the required track changes and the point-by-point reply.

Our decision is nuanced, and indeed we discussed the paper among numerous editors. The main issue, as raised by referees 2 and 3, is that the numbers are consistent with existing knowledge, so why should Nature publish the paper? Of course we were well aware of the similarity of your numbers to those already published and decided to proceed anyway. We continue to feel that the core conclusion is sufficient for Nature, and therefore overrule on the issue of importance. Still, the text must be altered in order to clarify the advance. The first paragraph already does so, to a certain extent, but must be further modified to contain wording to the effect of "the advance of our paper is not to modify the existing numbers … indeed we reach largely the same conclusions … instead we provide an effort to harmonize and homogenize the strikingly different existing methodologies … by doing so we provide enhanced confidence that our current understanding is robust to a wide range of methodologies". In short, the first paragraph should more transparently incorporate the spirit of referee 1's supportive comments.

Response: In view of the upcoming IPCC AR7, we believe it is of great relevance to validate the previous state of knowledge based on a much larger observational baseline. While our new assessment confirms the glacier mass-change estimates globally (within error bars), we found larger differences to previous IPCC assessment reports at a regional scale and systematic differences among observation methods. To clarify the advance of our study, we made the following improvements:

+ We revised the first paragraph, which now reads: "**While our results generally confirm the findings from previous assessments**[14–16] **at a global scale, we found some large regional differences, partly within random errors, suggesting a systematic error among observation methods. Our results provide a new baseline for better understanding observational differences and for calibrating model ensembles**[12,16,18]**, which will help to narrow down projections for the 21st century**[11,12,18]**.**"

+ We added a related statement to the main text, Comparison to IPCC estimates and outlook: "Our results generally confirm glacier mass-loss trends reported by the latest IPCC reports. Over common periods, our combined estimates of global glacier mass changes are less negative (but within uncertainties) by 4%, 9%, and 8% than estimates by IPCC AR5[47], SROCC[15], and AR6[16], respectively (Extended Data Table 3). At a regional level, the differences to the results from AR6 range between −20% for the Southern Andes and +53% for the Antarctic and Subantarctic Islands and are often within uncertainties (Extended Data Table 3)."

+ We also added regional results and differences compared to IPCC AR6 to Extended Data Table 3 (new rows highlighted in red track changes).

Extended Data Table 3 | Glacier mass-change rates compared to IPCC results

Source	Period	Mass change (IPCC) (Gt yr^{-1})	Mass change (this study) (Gt yr^{-1})	Δ (%)
IPCC AR5, WG1, Table 4.4, observations	2003–2009	−259 ± 28	−250 ± 27	+3%
IPCC SROCC, Table 2 A.1, observations	2006–2015	−278 ± 113	−254 ± 22	+9%
IPCC AR6, WG1, Chapter 9.5.1, observations	2000–2019	−266 ± 16	−246 ± 17	+8%
01 Alaska	2000–2019	−66.8 ± 5.2	−61.7 ± 7.0	+8%
02 Western Canada & USA	2000–2019	−7.1 ± 2.5	−7.1 ± 0.9	+0%
03 Arctic Canada North	2000–2019	−30.5 ± 2.1	−29.6 ± 2.9	+3%
04 Arctic Canada South	2000–2019	−26.6 ± 2.0	−23.2 ± 2.4	+13%
05 Greenland Periphery	2000–2019	−38.6 ± 3.6	−30.6 ± 7.2	+21%
06 Iceland	2000–2019	−9.5 ± 1.1	−8.4 ± 1.3	+11%
07 Svalbard & Jan Mayen	2000–2019	−9.2 ± 6.1	−10.7 ± 1.5	−16%
08 Scandinavia	2000–2019	−1.7 ± 0.2	−1.7 ± 0.1	+1%
09 Russian Arctic	2000–2019	−10.3 ± 1.0	−12.2 ± 2.2	−19%
10 North Asia	2000–2019	−1.2 ± 0.2	−1.3 ± 0.1	−4%
11 Central Europe	2000–2019	−1.6 ± 0.5	−1.7 ± 0.1	−6%
12 Caucasus & Middle East	2000–2019	−0.6 ± 0.1	−0.7 ± 0.1	−18%
13–15 High Mountain Asia	2000–2019	−20.0 ± 3.9	−19.0 ± 3.4	+5%
16 Low Latitudes	2000–2019	−1.1 ± 0.1	−0.9 ± 0.1	+20%
17 Southern Andes	2000–2019	−21.2 ± 6.8	−25.5 ± 6.7	−20%
18 New Zealand	2000–2019	−0.8 ± 0.1	−0.8 ± 0.1	+4%
19 Antarctic & Subantarctic Islands	2000–2019	−22.6 ± 2.7	−10.6 ± 9.0	+53%
IPCC AR6, WG1, Figure 9.21, model median	2007–2023	−347 ± 125	−300 ± 19	+16%
IPCC AR6, WG1, Table 9.SM.2, RCP 2.6	2015–2100	−333 ± 236	(2015–2023:) −323 ± 27	+3%
IPCC AR6, WG1, Table 9.SM.2, RCP 4.5	2015–2100	−502 ± 316	(2015–2023:) −323 ± 27	+36%
IPCC AR6, WG1, Table 9.SM.2, RCP 8.5	2015–2100	−670 ± 363	(2015–2023:) −323 ± 27	+52%

Most of the referees' other comments are largely straightforward and do not require amplification from me, but I should provide guidance on a few issues:

1) Referee 2 feels that the paper is too complex for Nature's format. We have some sympathy for the referee's viewpoint, and recognize the extreme difficulty of succinctly describing the sprawling methodology (in any journal!). To address the referee's concern, perhaps you could begin the Methods section with a paragraph containing a plain language summary/overview? The same paragraph could also note that the full details are available in the code (citing the Code Availability Statement). That way readers could be notified at the start of the Methods that all necessary details are in fact available, and that the Methods are used to provide an overview not an exhaustive step-by-step guide. You might also increase the citations to ED figures 1 and 2 and SI table 1, so that interested readers are pointed more frequently to the necessary information. Happy to discuss!

Response: We agree that GlaMBIE was a major endeavour in compiling and analysing the glacier results from the diverse observation methods in all 19 regions. At the same time, we are convinced that showcasing the main results with a high-level discussion of the methodological context will be of high interest to the broad readership of Nature, while the methodological details of the individual contributions are provided in more technical journals (with references provided in Supplementary Data Table 1). We believe that our community-based publication is the only feasible way to compile and compare all regional estimates in a homogenised way. We followed the editors' suggestions and made the following improvements:

+ We revised introduction paragraph to the Methods as follows: "The Glacier Mass Balance Intercomparison Exercise (GlaMBIE; https://glambie.org) is a community effort to compile, homogenize, combine, and analyse regional estimates of glacier mass changes from four distinct observation methods, or hybrids of observation methods: glaciological observations, DEM differencing, altimetry, and gravimetry (Extended Data Figure 1). In total, we analysed 233 regional estimates of glacier mass changes from about 450 data contributors organized in 35 research teams (Supplementary Data Table 1). Data contributions were selected based on expert evaluation of their confidence levels. Within each observation method, the selected input data were homogenized for time, space, and unit domains using common corrections. They were then combined first within and second among methods for each of the 19 regions and finally aggregated to global estimates. Below, we briefly summarize the input data (Extended Data Figure 1) and the general workflow (Extended Data Figure 2) of our intercomparison exercise, including key equations. The full methodological details are available in our code (Code availability). For more information on the different observation methods, we refer to a recent review on measuring glacier mass changes from space[26] and the methods references of our input data as listed in Supplement Data Table 1. As an output, GlaMBIE provides the native input data in a standardized format, combined estimates per observation method, and combined estimates among observation methods (Data availability). Given the available data, we consider our combined estimate (among methods) to best reflect the expert evaluation of the GlaMBIE community."

+ We added additional citations to ED Figures 1, 2, and Supplementary Data Table 1 in the main text and Method section so that interested readers can easily find the necessary information (see the track-change version of the revised manuscript).

2) We understand that referee 3 feels you should be focusing on individual glaciers. From our understanding of your approach, doing so would be impossible (because of GRACE's large footprint). If we have the right end of the stick here, it would be best to clarify the rationale within the main text.

Response: Indeed, current estimates from altimetry and gravimetry are only available at a regional scale, and this is only for regions with a large glacier area. However, we agree that future research should aim for intercomparisons at higher spatial and temporal resolutions. We clarify the rationale and provide an outlook in the main text:

+ Glacier mass balance intercomparison: "These estimates are based on one or more of the four observation methods (glaciological, DEM differencing, altimetry, gravimetry) and cover the period since the early 2000s. While the glaciological method and DEM differencing can provide results for individual glaciers with annual and decadal resolution, respectively, current estimates from altimetry and gravimetry are only available on a regional scale but can provide monthly variations. Similarly, DEM differencing and altimetry observe elevation changes, while the glaciological method and gravimetry provide mass changes. To account for the strengths and limitations of the different methods, we collected all regional datasets in the native units (i.e., metre, metre water equivalent, gigatonnes) and at the temporal resolutions specified by the participants (i.e., monthly, annual, multi-annual)."
[…]

"This offers a new observational baseline for future studies to better understand climate change's impact on glaciers and the associated downstream effects at the regional scale. While the current annual resolution estimates are sufficient to assess the contribution of glaciers to sea-level rise, a monthly (or higher) temporal resolution is needed to quantify the contribution of glaciers to the hydrological cycle[6]."

+ Differences among observation methods: "To further develop the applied observation methods, an intercomparison at higher spatial and temporal resolution is needed. As such, mass-change estimates at glacier (or higher) spatial resolution are required to identify the sources of observational differences[26,46]."

3) Given the comments from referees 2 and 3, we wonder if you could expand on the wider importance of the work, beyond the quantification and harmonization. It seems, for example, that your approach could allow you to provide guidance on the key remaining uncertainties, perhaps as a slight expansion of the concluding section?

Response: In the main text, we expanded the last paragraph of the section on Differences among observation methods to highlight the importance of our work: "With the quantification of differences among observation methods, GlaMBIE opens the door for further research to understand better and reduce the discrepancies between observation methods. As an example, a first detailed comparison[28] of altimetry and DEM differencing showed that differences in observed change rates do not necessarily stem from differences in the methods' spatial coverage (e.g., in ice margins and areas of poor radiometric contrast) but exist across the entire glacier hypsometry. In addition, GlaMBIE shows the need for homogenization of uncertainty assessments[32] and improved estimates of volume-to-mass conversion, especially over short survey periods[29]. To further develop the applied observation methods, an intercomparison at higher spatial and temporal resolution is needed. As such, mass-change estimates at glacier (or higher) spatial resolution are required to identify the sources of observational differences[26,46]. Reference data at the glacier scale, such as from airborne laser surveys, are less prone to errors than space-borne observations and can be used for validation. Validation of regional mass changes remains a challenge, and future efforts should be directed towards using high-quality, independent observations at multiple local sites to quantify uncertainties better and propagate these to the regional scale."

4) Please cite and discuss the Dussaillant et al. (2024) paper mentioned by referee 3. Note too that you should have let us know of the submission (see https://www.nature.com/nature-portfolio/editorial-policies/plagiarism#policy-on-duplicate-publication).

Response: Dussaillant et al. (2024, in review, Earth System Science Data) is a data & method paper related to one of the (hybrid) input data sets. As such, it complements the original reference of Dussaillant et al. (2023, https://doi.org/10.24381/cds.ba597449). All input data used for the combined GlaMBIE estimates were required to provide a method reference as listed in Supplement Data Table 1. As these publications are related to input data but not to the GlaMBIE

results, we have not considered related updates as duplicate publications but rather as improving the full transparency of our input data. We apologise for potential misunderstandings.

With the revision of our manuscript, we completed and updated the method references of the input datasets in Supplement Data Table 1 as follows:

- Jakob et al., Altimetry: Jakob and Gourmelen (2023) complemented with Jakob et al. (2021)
- Piermattei et al., DEM differencing: Piermattei et al. (2023, in review) updated by Piermattei et al. (2024, The Cryosphere)
- Dussaillant et al., Hybrid: Dussaillant et al. (2023, C3S) complemented with Dussaillant et al. (2024 in review, Earth System Science Data).
- Huss et al., Hybrid: Huss et al. (in preparation) replaced by Zemp et al. (2019, Nature)
- Ke et al., Hybrid: Ke et al. (2022, J. Glaciol.) corrected to Fan et al. (2023, J. Glaciol.)
- Harig et al., Gravimetry: Harig and Simons (2016, GRL) complemented with Beveridge et al. (2018, J. Geodetic Sci.)
- Sutterley et al., Gravimetry: Sutterley et al. (in prep.) replaced by Sutterley and Velicogna (2019, Remote Sensing), Sutterley et al. (2020, Earth and Space Science)
- Abdullahi et al., DEM differencing: Abdullahi et al. (2019) complemented with Wessel et al. (2016)

One dataset (Menounos et al., in situ) was submitted without method reference. However, this dataset was excluded from the GlaMBIE estimates as it only covered one year of observations (see Supplementary Data Figure 2). We decided to keep it on the list to ensure full transparency in GlaMBIE.

We next have specific formatting requirements, followed by our general guidelines.

- Provide stand-alone figures both for the main text and Extended Data (ED) items (details below). Main text figures may be AI, Vector EPS, layered PSD, postscript, PDF, PowerPoint, Word, or Excel files. ED files (figures and tables) may be JPG, TIF, or EPS. For multi-panel figures, submit a single file with the panels organized as you would like, rather than as separate files for each panel.

Response: We provide all main text figures as PDFs and ED figures as JPGs. Multi-panel figures were submitted in a single file.

- For the main text figures, please ensure that all text items are vectorized. Here is the specific guidance provided by my colleagues in our Art team: "... panels and figures ideally should be supplied as vector artwork – where lines, arrows, scale bars and text, etc. are all in an editable format and labels are on separate layers to images."

Response: We provide all our main text figures as vector artwork in PDF format. Please let us know if any of the figures have specific issues.

- To the extent possible, please use declarative titles for the figure legends (i.e. state the figure's main point).

Response: We double-checked all figures titles to state the main points deleted "Overview of" from the following ones:

+ Figure 1: "Global glacier mass changes from 2000 to 2023"

+ Extended Data Figure 1: "Data submissions to the intercomparison exercise"

- The uncertainty ranges in SI figures 1-19 should be described. We suggest adding a short paragraph immediately prior to SI figure 1 with a heading like "Description of Supplementary figures 1-19". The paragraph should describe the overall layout and uncertainties.

Response: We added a corresponding paragraph before SI Figures 1-19, including a statement on uncertainty ranges:

"Description of Supplementary Data Figures 1−19

Supplementary Data Figures 1−19 show data submissions and GlaMBIE results for the 19 glacier regions. The subplots show the sample size of submitted estimates (a) with combined results within and among methods as annual (b) and cumulative (c) specific mass changes. Submitted and combined estimates are shown as cumulative specific mass changes for altimetry (d), DEM differencing & glaciological observations (e), and gravimetry (f). In the legends, we indicate for each data submission if the variability [v] and/or trend [t] were used to calculate the combined estimate. Datasets not used are shown as grey dotted lines. Data submissions from hybrid approaches are marked (*) and shown in subplots of the assigned observation method. Uncertainties are given at 95% confidence intervals."

- Ensure that your color schemes are appropriate for readers with some form of visual impairment. For example, please check that all figures are accessible for readers with red-green colorblindness.

Response: We used color-blind-safe color schemes based on ColorBrewer (https://colorbrewer2.org), complemented with different line styles to ensure readability.

- Ensure that all data sources and literature are appropriately cited in the figure legends, and precisely clarify which datasets arise from your own work and which are from other sources.

Response: Done.

- Our rights department is checking permissions with great zeal, and this can cause delays of many weeks. Therefore, in the Data Availability Statement, please note software and base maps used for any figure using a map. Also, for any figure showing data from a published source or website/archive, please include all necessary citations in the figure legend itself. If re-use permission is required please provide statements of permission.

Response: We added the following sentence to the Data Availability Statement: "Figure 1 and Extended Data Figure 2 used global background maps from Natural Earth[110] and glacier regions defined by the Global Terrestrial Network for Glaciers[51]."

- Limit the main text references to no more than 50, with the remainder placed in a new Methods References section.

Response: We reduced the main text references to 50 by moving citations to the Method section.

- The Supplementary Information should contain its own references section. All references in the SI should be cited as in the main text, i.e. numerical and superscripted.

Response: Done. We added all citations in the SI to a Supplementary References section.

- From a quick glance it looks like some of the references in SI table 1 are not referenced anywhere (e.g. Foresta et al. (2016) and Khan et al. (2022)). Apologies if we missed! In any case, please ensure that everything in SI table 1 is in a reference list.

Response: Done. All references are now in the Supplementary References and in the Methods section.

- Any references cited only in the SI must also be included in the Methods References. This is because SI are not indexed and we should ensure that proper credit is provided to all sources. Therefore please copy any SI-only references to the Methods References and add to the Methods a sentence along the lines of "Further details are available in the Supplementary Information and rely on references X-Y."

Response: That's a good point! We have now included all SI references in the Methods. We added the method references related to the input data to the corresponding observation method section, adding "using various methods1,2,3."

- Organize your paper as: title page, text, references, table, figure legends, methods, data and code availability statements, methods references, acknowledgements, author contributions, author information (containing interest declaration and corresponding author line), Supplementary Information line, and extended data legends.

Response: The manuscript is organized in the suggested order with two minor deviations:

+ We kept one references list (main text references, followed by method references) to keep the link and automatic numbering by Zotero.

+ We provided all figures as separate files in the requested formats (see above) but kept them also in the manuscript files to ensure correct linking between figure legends and figures.

Now on to the general guidelines …

Please note that the presentation of a Nature paper has undergone significant changes in recent years to improve its readability and navigability online. Specifically, in most instances any supplementary text and data figures/tables can now be integrated into the main paper rather than presented in a separate Supplementary Information file. An overview of the key features and differences may be found in the Composition of a Nature Paper (http://s3-service-broker-live-19ea8b98-4d41-4cb4-be4c-d68f4963b7dd.s3.amazonaws.com/uploads/ckeditor/attachments/7824/3g_Paper_composition.pdf).

Response: We checked the revised composition and organized our features accordingly.

SUMMARY PARAGRAPH: All Nature papers begin with a fully referenced paragraph, typically no longer than 200 words, aimed at readers in other disciplines. This paragraph starts with a 2- to 3-sentence, basic introduction to the field; continues with a 1-sentence statement of the main findings starting 'Here we show' or an equivalent phrase; and finally, concludes with 2 to 3 sentences putting the main findings into general context so it is clear how the results described in the paper have moved the field forward. A downloadable, annotated example is available at https://www.nature.com/nature/for-authors/formatting-guide. In some cases it may be necessary to exceed this limit in order to explain complex material for readers in other fields – in such cases, summary paragraphs can be up to 230 words in length. The extra length, however, is for introduction and context, and not for additional technical information.

Response: We follow the suggested structure of the summary paragraph. After the revisions based on feedback from the Editor (see above) and Referees (see below), its length is 246 words. If required, we could shorten it by deleting the following sentences:

"With this, glacier mass loss is about 18% larger than the loss from the Greenland Ice Sheet and more than twice that from the Antarctic Ice Sheet[17]."

MAIN TEXT: If further introductory material is necessary, the main text can begin with up to 500 words of introduction expanding on the background to the work (some overlap with the summary paragraph is acceptable), before proceeding to a concise, focused account of the new research and findings, and ending with 1 or 2 short paragraphs of discussion. Sections are separated with subheadings to aid navigation. Subheadings may be up to 40 characters (including spaces).

Response: We follow the suggested structure and guidelines of the main text.

STATISTICS: Authors should ensure that any statistical analysis used is sound and that it conforms to the journal's guidelines (see https://www.nature.com/nature/for-authors/formatting-guide for guidance).

Response: Our statistical analysis conforms with the journal's guidelines.

METHODS: At the end of the main text document (after the main figure legends), there should

be a section entitled "Methods", which provides a more detailed discussion of the additional methodological information that would allow other researchers to replicate the results (we define “Methods” quite broadly, so this is not limited to details of experimental protocols – supplementary discussion and analysis can also be included). The Methods section will not appear in the print version but will be fully copy-edited and appear online in the full-text HTML and PDF versions. The Methods section should be written as concisely as possible but should contain all elements necessary to allow interpretation and reproduction of the results. If there are additional references in the Methods section, their numbering should continue from the last reference in the main paper, and the list should follow the Methods section.

Response: We follow the suggested structure and guidelines of the Methods section.

REFERENCES: As a guideline, Articles allow up to 50 references in the main text; additional references can be cited in (and listed after) the Methods section, as detailed above.

Response: Our article has 50 references in the main text and an additional 61 references in the Methods section.

MAIN TEXT STATEMENTS: We require authors to provide a detailed Author Contribution statement immediately after the acknowledgements; the specific contributions of each author must be listed. It is also a condition of publication that authors include an Author Information statement indicating how to access information regarding reprints and permissions, stating whether or not there is a financial or non-financial competing interest, and naming the author to whom correspondence and requests for materials should be addressed. Please ensure that this section is included in the manuscript file after the Methods (but before the Extended Data legends) - it will not appear in the print version but will appear online in the full-text HTML and PDF versions. For details of "end note" style and an example see https://www.nature.com/nature/for-authors/formatting-guide.

Response: We provide a detailed Author Contribution statement, following the Contributor Role Taxonomy (CrediT, Allen et al. 2019), which is graphically shown in Supplementary Data Figure 20.

FIGURE LEGENDS: These should be listed sequentially after the references in the main text and not in the figures files. Each legend should begin with a brief title for the whole figure and continue with a short description of each panel and the symbols used. Any error bars in the figures must be defined (for example, s.d., s.e.m.) and the value of n indicated; see https://www.nature.com/nature/for-authors/formatting-guide for further explanation.

Response: We followed the suggested guidelines for figure legends. In addition, we added the following statement at the end of the Methods section: “We note that uncertainties (σ) in the Methods section (above) are formulated at the one-sigma level (i.e., 68% confidence interval) to simplify equations, while results in the main text, figures, and tables are reported at the 1.96-sigma level (i.e., 95% confidence interval).”

DISPLAY ITEMS: We ask that you take stock of all the data that have been generated throughout the review process and ensure that only the data most central to the conclusions are presented in the main figures. Figures should be comprehensible to readers in other or related disciplines, and assist their understanding of the paper. We encourage authors who are describing complex processes to include a schematic of the main finding as part of the Extended Data to aid readers unfamiliar with the immediate discipline. Figures should be as small and simple as is compatible with clarity. All panels of a figure should be logically connected; each panel of a multipart figure should be sized so that the whole figure can be reduced by the same amount and reproduced on the printed page at the smallest size at which essential details are visible. For guidance, Nature's standard figure sizes are 89 mm (one column), 120 mm (one and a half columns), or exceptionally 183 mm (two columns) wide; the full depth of a Nature page is 247 mm. All panels of figures should be presented on a single page and assembled into a rectangular shape for publication; please indicate any essential alignments (parts horizontal, vertical, spacings of stereo pairs, etc.). Tables should be prepared using the Table menu in Microsoft Word.

Response: We followed the suggested guidelines for display items.

FIGURE FORMATTING: Lettering in all figures (labelling of axes and so on) should be in uniform, sans-serif font, in lower-case type, and large enough to permit substantial reduction for publication (minimum font size 5 pt). Separate parts of a figure are labelled a, b, etc. Units have a single space between the number and the unit, and follow SI nomenclature or the nomenclature common to a particular field. Thousands are separated by commas (1,000). Unusual units or abbreviations are defined in the legend. Scale bars rather than magnification factors should be used.

Response: We followed the suggested guidelines for figure formatting.

IMAGE PRESENTATION: Authors should be aware that any image provided for publication, either in print or online (as Extended Data or Supplemental Information), may be subject to a quality control process to check for image integrity and manipulation. For a full discussion of our standards regarding how images should be prepared and presented, see www.nature.com/authors/editorial_policies/image.html.

Response: Our article has no images.

EXTENDED DATA: Nature is now integrating the supplemental figures and tables into the final version of most papers. Extended Data do not appear in the printed version of the paper but are included online within the full-text HTML and at the end of the online PDF. Extended Data are an integral part of the paper and only data that directly contribute to the main message should be presented. All Extended Data must be referred to in the main text, figure legends and/or Methods section, and their figure legends should be listed sequentially at the end of the main text, not in the Extended Data files. Authors should assemble the Extended Data into a maximum of ten, multi-panelled display items, submitted as individual JPEG, TIFF or EPS files. They must be provided at the same quality as figures for print, but there are important differences in their

formatting. More specific instructions are provided in the Extended Data Formatting Guide (http://s3-service-broker-live-19ea8b98-4d41-4cb4-be4c-d68f4963b7dd.s3.amazonaws.com/uploads/ckeditor/attachments/7823/3h_Extended_data.pdf).

Response: We followed the suggested guidelines for extended data.

SUPPLEMENTARY INFORMATION: Supplementary Information is online-only material published with the manuscript (https://www.nature.com/nature/for-authors/supp-info). For most papers, there should be no need for Supplementary Information beyond that already provided as Methods and Extended Data, the aim being to avoid unnecessary fragmentation of the paper online. Exceptions to this rule include large datasets that cannot be accommodated within Extended Data; video material; and more complex "Supplemental Methods" (and any associated references) that do not readily fit within the constraints of the Methods/Extended Data formats. Please note that after the paper has been formally accepted you can only provide amended Supplementary Information files for critical changes to the scientific content, not for style. You should clearly explain what changes have been made if you do resupply any such files.

Response: We followed the suggested guidelines for supplementary information and made one deviation. Given the limited number of Extended Data features, we provided full-page figures of our input data and results for all 19 glacier regions as Supplementary Data Figures 1-19. We believe that these figures are important to document the full transparency of our results.

SOURCE DATA: To further increase transparency, we encourage authors to provide, in spreadsheet form, the data underlying the graphical representations used in the figures. This is in addition to our well-established data-deposition policy for specific types of experiments and large datasets. Readers of the online manuscript will be able to access the Source Data directly from the figure legend. Spreadsheets can only be submitted in .xls, .xlsx or .csv formats. One file per figure is permitted; thus, if there is a multi-panelled figure the Source Data for each panel should be clearly labeled in the csv/Excel file; alternatively the data for a figure can be included in multiple, clearly labeled sheets within an Excel file. File sizes of up to 30 MB are permitted; however, it is expected that the vast majority of Source Data files will be considerably smaller than this. When submitting these files with your manuscript, please select the file type "Source Data" and use the title field in the file description tab to indicate the figure to which the Source Data pertain.

Response: With the revised manuscript, we provide source data for all main Figures and Extended Data Figures, except for ED Fig. 2, which shows the GlaMBIE workflow. The source data is organised in one Excel file with separate worksheets per figure.

THIRD PARTY RIGHTS:
While you finalise your manuscript, please identify any content used in your article — whether in the main text, extended data or supplementary information — that comes from a third party. This could include figures, tables, images, videos or text boxes that are reproductions or adaptations of items that have previously been published elsewhere and/or are owned by a third party. It also encompasses pictures taken by professional photographers, maps and images downloaded from the internet. You must obtain the right to use each of these items and you will

need to provide evidence that you have these rights before your paper can be accepted for publication. You will also need to give proper attribution to the copyright holders in your paper. Failure to obtain the appropriate rights will delay the publication of your article. Please fill out a Third Party Rights Table, and upload this to our manuscript tracking system with your manuscript. You can find more information about third party rights on our Rights and permissions page.

Response: Our manuscript uses only open-source material, including proper citation, and does not use content with third-party rights.

When revising your paper:

* Include a point-by-point response to our referees and to our editorial suggestions. We are committed to providing a fair and constructive peer-review process. Please do not hesitate to contact us if there are specific requests from the reviewers that you believe are technically impossible or unlikely to yield a meaningful outcome.

Response: The present file provides our point-by-point response to referees and editorial suggestions.

* Ensure it complies with our format requirements for Articles as set out at https://www.nature.com/nature/for-authors

Response: We followed the format requirements.

* Resubmit electronically using the following link: […]

Response: Done

* This url links to your confidential home page and associated information about manuscripts you may have submitted or be reviewing for us. If you wish to forward this email to co-authors, please delete the link to your homepage first.

Response: Personal URL removed.

All correspondence should contain your Nature reference number in the subject line.

We look forward to hearing from you soon, ideally within four weeks.

Yours sincerely
Michael

Dr Michael White
Senior Editor, Nature
San Francisco
ORCID iD 0000-0002-0238-8913

+ +

Referee #1 (Remarks to the Author):

Over the past two decades, a plethora of research has concentrated on examining the mass balance of glaciers within specific regions. These studies have consistently reported a sustained and, in some cases, an accelerated rate of glacier mass loss across the globe. Nevertheless, a limited number of investigations have attempted to assess the mass balance of glaciers on a global scale. Furthermore, the existing studies in this domain have predominantly relied on a singular data methodology. The article introduces innovative aspects in assessing global glacier mass changes through the Glacier Mass Balance Intercomparison Exercise (GlaMBIE), which includes a comprehensive inter-comparison across various observation methods, robust data homogenization processes, a novel five-step methodology for data combination, detailed uncertainty quantification, and the creation of a new observational baseline.
The research presented in this article is highly laudable for its methodological rigor and significant contributions to the field. The academic community concerned with glacier mass loss has directed significant attention towards understanding the glaciers' response to climate change and the consequential impacts of these changes on sea-level dynamics. To the best of my knowledge, no prior research has endeavored to quantify the global glacier mass balance employing a diverse array of observational techniques and datasets. This work built a new method for data homogenization and combination and, in doing so, will redirect future effects of the glacier mass balance community. The quality of both the experimental approach and treatment of uncertainty is excellent and the writing style is quite clear. The figures included in the main text are visually appealing. The references are appropriate and complete.

Response: We thank Referee #1 for the positive feedback and clarified the minor points outlined below.

I have one minor concerns with the work at present, though both should be readily addressed with some simple clarifications, discussion of assumptions, and a supplementary figure.

My primary concern is the glacier mass in 2000. To account for glacier shrinkage, the authors used regional glacier area-change rates compiled from IPCC AR5 and corrected glacier area from RGI 6.0 to the year 2000 and computed yearly time series of regional glacier area. Regional glacier volume and mass area from the Ice Thickness Models Intercomparison eXperiment (ITMIX). Given that the author has corrected glacier area from RGI 6.0 to the year 2000, it is imperative that the glacier volume and mass estimates be similarly adjusted. This is essential as the ITMIX dataset does not accurately reflect the volume and mass of glaciers as of the year 2000. The authors should pay more attention to clarify it.

Response: We agree and – in fact – did correct the regional glacier area from RGI 6.0 <u>and</u> the regional glacier volume and mass from ITMIX, which is based on RGI 6.0, to the year 2000. The manuscript described this in the Methods section “Glacier area, volume, and mass in 2000”, Lines 297−303.

We slightly revise the corresponding section to emphasise better the correction of glacier volume and mass to the year 2000: “Regional glacier volume and mass are from a multi-model consensus ice thickness estimate[20], which was based on glacier outlines from RGI 6.0. We used their estimates relevant to sea-level rise, i.e., subtracting the ice fraction below present-day sea level. In addition, we corrected their values to the year 2000 (Table 1) by using annual mass changes after 2000 from our combined estimate (see below) and before 2000 from the input dataset by Dussaillant et al.[56], which provides mass-change estimates from 1976, combining glaciological observations with DEM differencing.”

In the main text, we point to this correction in the caption of Table 1: “Regional and global glacier area[19] and mass[20] corrected to the year 2000 (see Methods), …”

Minor line-by-line comments

Line 702, the reference 74 had been published in The Cryosphere, it could be revised the cite.

Response: We updated the method reference of the input data by Piermattei et al. (2023, in review => 2024, published, The Cryosphere), as well as several other references (see response to comment above).

+ +

Referee #2 (Remarks to the Author):

This manuscript provides a fairly comprehensive and lengthy analysis of global glacier mass changes from 2000 to 2023 based on inputs from different techniques and a large number of groups. There is no doubt that the study is important and should be published. My main concern is that if the results and presentations are fitted to Nature. I understand this intends to follow the footsteps of the two IMBIE Team’s publications in Nature in 2018 and 2020 on Greenland and Antarctic ice mass balance. However, given the scope of global glaciers and the way the results were organized, it would be very difficult for the general readers to comprehend.

Response: We thank Referee #2 for the feedback. Indeed, GlaMBIE follows the example of the successful IMBIE. Glaciers are an essential climate variable, the largest non-steric contributor to current sea-level rise, and a vital component of the water cycle. The research community and IPCC authors identified the need for a global assessment of glacier mass changes that combines results from all observation methods. We agree that this is a major endeavour requiring us to develop an approach tailored to glacier observation methods. We believe that presenting a high-level overview of the results and the strengths and limitations of the different observation methods is of interest to the broad readership of Nature.

Major comments:
1) The analysis, result presentation, and organization are fairly difficult to follow. As a researcher with over 20 years experience in related fields, I myself had a really hard time to

follow the analysis, data processing and results interpretation. This is not necessarily the authors' fault. The target here (global glaciers) is simply too broad.

The two IMBIE Teams did a good job in putting together two very concise short papers that are both important and can be easily understood by the general readers. Greenland and Antarctic ice sheets are two single targets, with one covered by each of the IMBIE papers. In comparisons, there are 19 target regions in the present analysis.

The complexity is caused by not only the large number of target regions in the present study, but also the different techniques used for different regions and different groups providing the different estimates.

Response: We agree that including all 19 glacier regions and all the different observation methods makes the exercise more complex than the equivalent IMBIE papers. At the same time, we are convinced that bringing together all these results in a well-defined intercomparison exercise is a major advance in our understanding, complements and adds to the work of IMBIE, and is an essential contribution towards the following IPCC report. Future research will benefit from the total transparency of GlaMBIE, which summarised and discussed global and regional results in the context of the current state of knowledge and provided open access to the code and all data used and produced. We revised the introduction of the Methods section to summarise our approach in plain language and refer to the Code and Data Availability statements.

Methods: "The Glacier Mass Balance Intercomparison Exercise (GlaMBIE; https://glambie.org) is a community effort to compile, homogenize, combine, and analyse regional estimates of glacier mass changes from four distinct observation methods, or hybrids of observation methods: glaciological observations, DEM differencing, altimetry, and gravimetry (Extended Data Figure 1). In total, we analysed 233 regional estimates of glacier mass changes from about 450 data contributors organized in 35 research teams (Supplementary Data Table 1). Data contributions were selected based on expert evaluation of their confidence levels. Within each observation method, the selected input data were homogenized for time, space, and unit domains using common corrections. They were then combined first within and second among methods for each of the 19 regions and finally aggregated to global estimates. Below, we briefly summarize the input data (Extended Data Figure 1) and the general workflow (Extended Data Figure 2) of our intercomparison exercise, including key equations. The full methodological details are available in our code (Code availability). For more information on the different observation methods, we refer to a recent review on measuring glacier mass changes from space[26] and the methods references of our input data as listed in Supplement Data Table 1. As an output, GlaMBIE provides the native input data in a standardized format, combined estimates per observation method, and combined estimates among observation methods (Data availability). Given the available data, we consider our combined estimate (among methods) to best reflect the expert evaluation of the GlaMBIE community."

2) While this community assessment of global glaciers mass balance is important, the main conclusions are not really new and some are mostly a confirmation of a recent Nature paper by Hugonnet et al. (2020) (with its author/co-authors also on this GlaMBIE Team). This is another reason that I believe it would fit better to a more specialized journal.

Response: The global assessment based on DEM differencing by Hugonnet et al. (2021) was undoubtedly an important benchmark and provided the observational baseline in IPCC AR6. While our study confirmed their findings (within error bars) at a global scale and regarding long-term trends, we found larger differences at a regional scale and a systematic difference to the results from altimetry. Also, DEM differencing (as used by Hugonnet et al. 2021) is limited in reproducing the inter-annual variability. Following the editor's suggestion (see above), we revised the summary paragraph to emphasise these points better, and we added the regional differences to the IPCC AR6 to Extended Data Table 3. The differences between our results and IPCC AR6 and Hugonnet et al. (2021) are discussed in the main text section "Comparison to IPCC estimates and outlook".

+ Summary paragraph: "**While our results generally confirm the findings from previous assessments**[14–16] **at a global scale, we found some large regional differences, partly within random errors, suggesting a systematic error among observation methods. Our results provide a new baseline for better understanding observational differences and for calibrating model ensembles**[12,16,18]**, which will help to narrow down projections for the 21**st **century**[11,12,18]**.**"

+ Extended Data Table 3 (new rows highlighted in red track changes):

Extended Data Table 3 | Glacier mass-change rates compared to IPCC results

Source	Period	Mass change (IPCC) (Gt yr^{-1})	Mass change (this study) (Gt yr^{-1})	Δ (%)
IPCC AR5, WG1, Table 4.4, observations	2003–2009	−259 ± 28	−250 ± 27	+3%
IPCC SROCC, Table 2 A.1, observations	2006–2015	−278 ± 113	−254 ± 22	+9%
IPCC AR6, WG1, Chapter 9.5.1, observations	2000–2019	−266 ± 16	−246 ± 17	+8%
01 Alaska	2000–2019	−66.8 ± 5.2	−61.7 ± 7.0	+8%
02 Western Canada & USA	2000–2019	−7.1 ± 2.5	−7.1 ± 0.9	+0%
03 Arctic Canada North	2000–2019	−30.5 ± 2.1	−29.6 ± 2.9	+3%
04 Arctic Canada South	2000–2019	−26.6 ± 2.0	−23.2 ± 2.4	+13%
05 Greenland Periphery	2000–2019	−38.6 ± 3.6	−30.6 ± 7.2	+21%
06 Iceland	2000–2019	−9.5 ± 1.1	−8.4 ± 1.3	+11%
07 Svalbard & Jan Mayen	2000–2019	−9.2 ± 6.1	−10.7 ± 1.5	−16%
08 Scandinavia	2000–2019	−1.7 ± 0.2	−1.7 ± 0.1	+1%
09 Russian Arctic	2000–2019	−10.3 ± 1.0	−12.2 ± 2.2	−19%
10 North Asia	2000–2019	−1.2 ± 0.2	−1.3 ± 0.1	−4%
11 Central Europe	2000–2019	−1.6 ± 0.5	−1.7 ± 0.1	−6%
12 Caucasus & Middle East	2000–2019	−0.6 ± 0.1	−0.7 ± 0.1	−18%
13–15 High Mountain Asia	2000–2019	−20.0 ± 3.9	−19.0 ± 3.4	+5%
16 Low Latitudes	2000–2019	−1.1 ± 0.1	−0.9 ± 0.1	+20%
17 Southern Andes	2000–2019	−21.2 ± 6.8	−25.5 ± 6.7	−20%
18 New Zealand	2000–2019	−0.8 ± 0.1	−0.8 ± 0.1	+4%
19 Antarctic & Subantarctic Islands	2000–2019	−22.6 ± 2.7	−10.6 ± 9.0	+53%
IPCC AR6, WG1, Figure 9.21, model median	2007–2023	−347 ± 125	−300 ± 19	+16%
IPCC AR6, WG1, Table 9.SM.2, RCP 2.6	2015–2100	−333 ± 236	(2015–2023:) −323 ± 27	+3%
IPCC AR6, WG1, Table 9.SM.2, RCP 4.5	2015–2100	−502 ± 316	(2015–2023:) −323 ± 27	+36%
IPCC AR6, WG1, Table 9.SM.2, RCP 8.5	2015–2100	−670 ± 363	(2015–2023:) −323 ± 27	+52%

3) As a main conclusion, the authors claimed that the estimated glacier mass loss rate (-273 Gt/yr) over 2000-2023 is about 18% larger than the loss from the Greenland Ice Sheet and more than twice that from the Antarctic Ice Sheet and used Otosaka et al. (2023) as reference.

Otosaka et al. (2023) was a extended analysis of the IMBIE Team paper over a longer period of time 1992-2020. There were no separate Greenland and Antarctic mass rate estimates for the period 2000-2023 in Otosaka et al. (2023). I am not sure what the authors compared with. It could be likely from Figure 3 and corresponding descriptions in Otosaka et al. (2023) for Greenland and Antarctic estimates for the period 2003-2018.

Response: We compared our glacier mass-change rates to ice sheet results from Table 2 in Otosaka et al. (2023). Otosaka et al. provided mean annual change rates for 5-year periods since 2002, missing one year (i.e. 2021) in the last period (2017-2021). To allow for a sound comparison between 5-year periods, we compared our results from 2002-2021 (i.e., four pentads) to their results from 2002-2020, as described in the main text and further detailed in our Extended Data Table 1. Excluding the glacier mass changes for 2021 would slightly increase the glacier change rate (2002-20) from −262 to −264 Gt yr^{-1}., but still result in a (rounded) difference to the Greenland Ice Sheet of 18%.

Main text, Global and regional mass changes: "Compared to recent estimates[17] for the ice sheets from 2002 to 2021, glacier mass loss is about 18% (significant at 90% CI) larger than the loss from the Greenland Ice Sheet and more than twice the loss from the Antarctic Ice Sheet (Extended Data Table 1)."

Extended Data Table 1:

Extended Data Table 1 | Comparison of mass changes from glaciers and ice sheets

Period	Glaciers, global total (Gt yr^{-1})	Glaciers, Greenland Periphery (Gt yr^{-1})	Glaciers, Antarctic & Subantarctic Islands (Gt yr^{-1})	Ice Sheet, Greenland (Gt yr^{-1})	Ice Sheet, Antarctica (Gt yr^{-1})
2002–2006	−229 ± 34	−18 ± 15	−24 ± 19	−170 ± 39	−59 ± 41
2007–2011	−266 ± 32	−54 ± 14	−2 ± 17	−270 ± 38	−127 ± 45
2012–2016	−254 ± 30	−29 ± 13	0 ± 17	−203 ± 40	−147 ± 43
2017–2021*	−299 ± 32	−29 ± 14	−28 ± 18	−247 ± 42	−112 ± 55
2002–2021°	−262 ± 16	−32 ± 7	−13 ± 9	−223 ± 20	−111 ± 23

For ice sheets, rates are calculated for 2017–2020* and 2002–2020°.

To compare mass changes for peripheral glaciers (from this study) and for ice sheets (from IMBIE[12]) in Greenland and Antarctica, we calculated mean annual change rates (Gt yr^{-1}) for five-year periods from calendar years 2002 to 2021. Note that we corrected ice sheet estimates by −10 Gt yr^{-1} and −3 Gt yr^{-1} for the Greenland and Antarctica ice sheets, respectively, to account for the leaking effects of glacier signal into ice sheet estimates from gravimetry[12]. Uncertainties of glacier mass changes correspond to 95% confidence intervals, while ice sheet uncertainties are reported as one standard error[12].

Nevertheless the comparison is not valid given the different time spans (differing by at least 4-5 years).

Response: As explained above (and noted in Extended Data Table 1), our comparison is valid and refers to the common observation period from 2002 to 2020/21.

For unknown reasons, the gravimetry results in Otosaka et al. (2023; Figure 3) for Greenland over the period 2003-2018 (~ -220 Gt/yr) appear significantly smaller than similar GRACE and GRACE-FO estimates, e.g., -261 Gt/yr over the period 2002-2019 from Velicogna et al. (2020).

An updated gravimetry estimate of Greenland mass rate over the period 2002-2023 can be easily derived from the latest GRACE and GRACE-FO mascon solutions. This GlaMBIE Team includes several experts in satellite gravimetry and it should not be difficult.

Response: The differences in the ice sheet results between the 'consensus estimate' by Otosaka et al. (2023) and the individual estimate by Velicogna et al. (2020) can have several reasons, including differences in observation periods, corrections for GIA and ocean mass changes, filtering, etc. We consider our comparison between IMBIE (Otosaka et al. 2023) and GlaMBIE (this study) results to be valid and to come with correct reference to the source (i.e., Otosaka et al. 2023, Table 2). The objective of this study was to obtain estimates for the 19 glacier regions, and we believe that any update of ice sheet estimates is the preserve of researchers involved with IMBIE. 😉

4) If I understand correctly, the authors included Greenland Periphery and Antarctic & Subantarctic Islands in the global glacier mass change and then compared the estimated global glacier mass change with Greenland and Antarctic ice sheets mass change. There might be some inconsistency here, as satellite gravimetry could not separate those small signals from the nearby ice sheets mass changes. This is yet another indication that this analysis may fit better for a specialized journal, as there are not many people (except for glaciologists) who can really tell the difference between Greenland Periphery and Greenland glaciers.

Response: Our global glacier mass-change estimates in Table 1 cover all 19 regions, including peripheral glaciers in Greenland (region 05) and on the Antarctic and Subantarctic Islands (region 19). Our comparison between glacier and ice sheet estimates is given in Extended Data Table 1, including corrections to account for the leaking effects of glacier signal into ice sheet estimates from gravimetry as suggested by IMBIE (Otosaka et al. 2023).

To emphasise the difference between glaciers and ice sheets upfront, we updated the first sentence of the main text as follows: "Glaciers separate from the continental ice sheets in Greenland and Antarctica covered a global area of approximately 706,000 km^2 around the year 2000[19], with an estimated total volume of 158±41×10^3 km^3, equivalent to a potential sea-level rise of 324±84 mm[20]."

5) Since the inputs from different techniques, different groups and different series from the same group covered different time span, it is not clear how those data were combined. For example, the authors "analysed 78 geodetic assessments from gravimetry from 7 research teams, covering 17 out of 19 regions". What are those 78 geodetic assessments? Time spans? Targets?

Response: Full transparency of the input data and related methods is given in Extended Data Figure 1 (Sankey Diagram), Supplementary Data Table 1 (research teams, method references, regions), and Supplementary Data Figures 1-19 (regional plots of all input data including time spans, temporal resolution, and coverage). To better guide the reader to this information, we added the method references to all corresponding Method sections, e.g. for Gravimetry:

"For gravimetry, we analysed 78 data contributions from 7 research teams, covering 17 out of 19 regions using various methods[31,90–97] (Extended Data Figure 1, Supplementary Data Table 1)."

6) Line 27: “with an estimated total volume of 158±41×103 km3, equivalent to a potential sea-level rise of 32±8 mm”. This is wrong statement. The global ocean area is about 360 million square kilometers, and the 158×103 km3 glacier ice would be equivalent to a potential sea-level rise of 439 mm, more than one order larger than the number (32 mm) given here.

Response: Thanks for spotting this error, which was introduced when aligning units to mm sea-level rise! We corrected the value to the correct digits based on Farinotti et al. 2019, Table 1: “…with an estimated total volume of 158±41×10^3 km^3, equivalent to a potential sea-level rise of 324±84 mm[20].”

Note that the sea-level equivalent is corrected for the ice below sea level, as noted in the Methods section, Glacier area, volume and mass in 2000: “…We used their estimates relevant to sea-level rise, i.e., subtracting the ice fraction below present-day sea level.”

7) Extended Data Figure 1: It is a novel idea to illustrate the data submissions this way, but it is still very confusing, especially when different groups share the same color. There is no way to really track down the actual contributions from the different groups in this case.

Response: We updated the caption to clarify the meaning of the different colours and revised the figure to improve its readability by re-sorting the different lines according to observation methods. The revised figure allows tracking all estimates from research teams to sensors/products and observation methods. The link between research groups and regions is given in Supplementary Data Table 1.

Extended Data Figure 1 | Data submissions to the intercomparison exercise

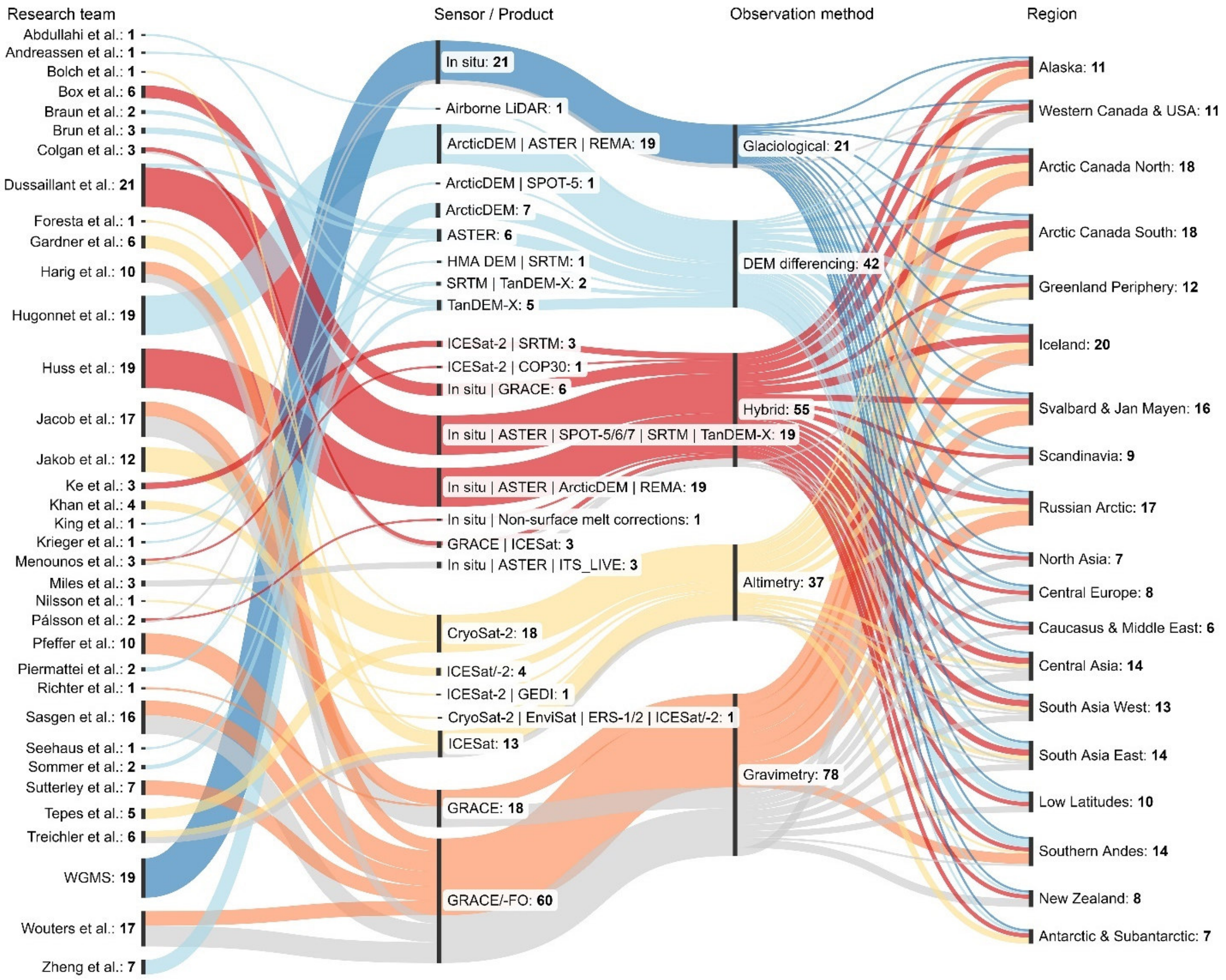

"The figure provides an overview of research teams (left, in alphabetic order) participating in GlaMBIE with their selection of sensors or products (middle left) used for computing glacier mass-change estimates from different observation methods (middle right) for the 19 regions (right). From the 233 regional results, 195 (coloured lines) were used to compute our combined estimates, and 38 (grey lines) were excluded based on regional confidence levels of observation methods (see Methods). Colours follow observation methods. A more detailed overview of all data contributions is given in Supplementary Data Table 1. The figure was produced with SankeyMATIC[112]."

8) Extended Data Figure 3: This is a busy graphic. Among the 19 regions, the time spans are mostly different, and none covers the period before 2002. Then how could the authors derive reliable estimates for the period 2000 – 2023? A time span was given for each of the 19 regions, with 4 estimates from the 4 techniques. Did the 4 techniques cover the same time span?

Response: The time spans are different among regions and observation methods. In Extended Data Figure 3 and Extended Data Table 2, we addressed this by comparing results over common observation periods separately for altimetry (left column) and gravimetry (right column). This approach allowed for a fair comparison among methods over a common observation period (as used in Extended Data Figure 3).

The results for the entire period from 2000−2023 (as shown in Table 1) are possible thanks to the full coverage of the glaciological observations, which were calibrated to the other methods over the common observation period (as explained in the Methods section and illustrated in Extended Data Figure 2).

We deleted the result values per method to make the figure less busy and will provide them in the figure's source data. In addition, we reworded the caption for more clarity:

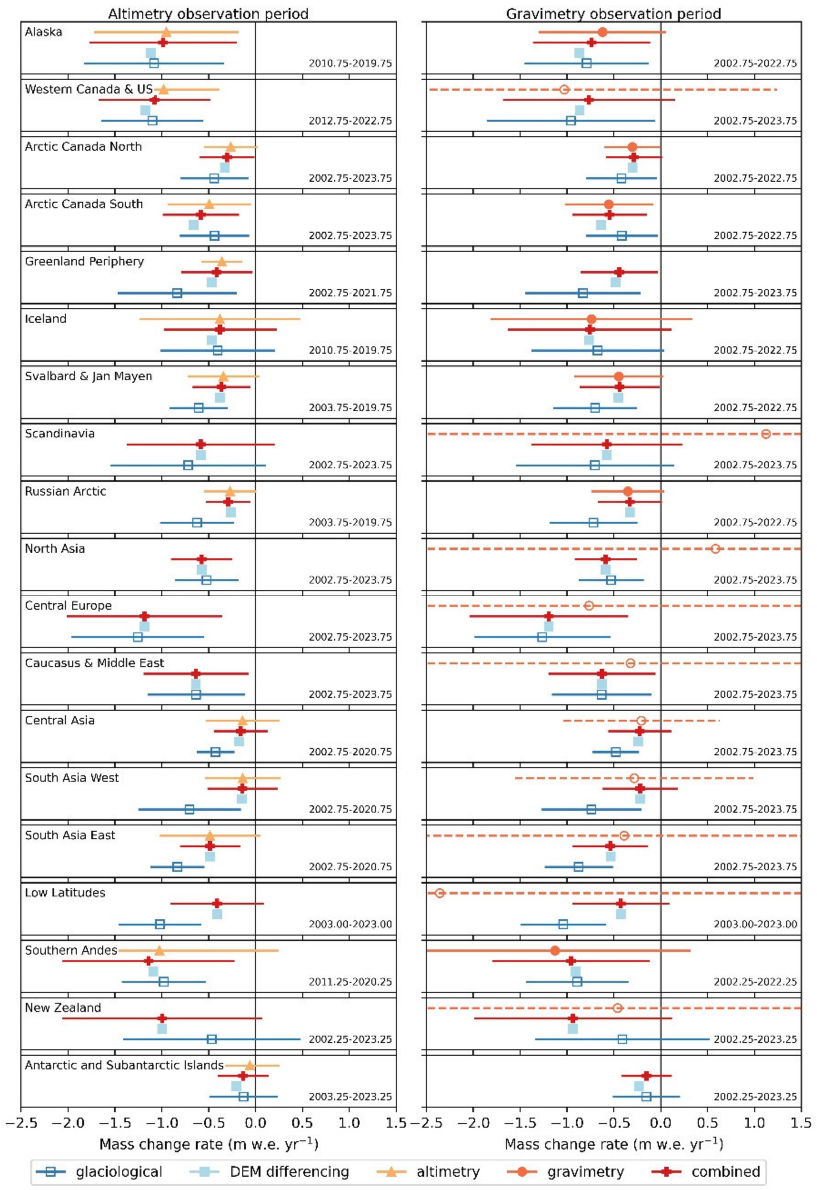

“For each region (rows), mean specific mass-change rates (shown as markers) and inter-annual variability (one standard deviation, shown as lines) are compared among different methods over common observation periods for altimetry (left column) and gravimetry (right column). The common observation periods are shown as decimal years (bottom right). The gravimetry period does not include the hydrological year 2007/08. The combined estimates include only the observation methods indicated by complete markers and solid lines. Empty markers or dashed lines indicate results not considered for the combined estimates. Differences between observation methods and related uncertainties are given in Extended Data Table 2.”

There are simple too many things to consider and too many issues to deal with in this comprehensive analysis. A more specialized journal should be a better fit for this work

Response: Our community estimate marks a major milestone in assessing global glacier changes from different observation methods. We believe that the related results and discussion are of great interest to the readers of Nature and provide an important baseline for future research - in this or more specialised journals - to better understand the observational differences and for calibrating model ensembles.

+ +

Referee #3 (Remarks to the Author):

A. Summary of the key results

The paper presents the findings of the GLAMBIE project (https://glambie.org/project-team/) – a community estimate of glacier mass change for the period from 2000 to 2023. The results have been generated following extensive community input, engagement, checking and homogenisation using data from a range of remote sensing approaches (DEM differencing, altimetry, gravimetry, hybrid methods) and direct mass balance observations (from a smaller subset of global glaciers). Noting that this work represents a huge and praiseworthy community effort, the overall results are not (within uncertainty) significantly different from the 6th IPCC assessment report or previous global studies. Given the uncertainties, it is also not clear that this new estimate is indeed more accurate than previous work.

Response: We thank Referee #3 for the feedback. Our new assessment confirms the glacier mass-change estimates of past IPCC reports globally (within error bars). However, we found some larger differences on a regional scale (partly within error bars), suggesting systematic differences among observation methods. These findings call for future research to improve impact studies and future projections.

We revised the main text to better highlight the progress of our study and added additional paragraphs to discuss the limitations and perspectives for future improvements of uncertainty estimates. Below, you find the revised version of the corresponding paragraphs.

+ We revised the first paragraph: "**While our results generally confirm the findings from previous assessments[14–16] at a global scale, we found some large regional differences, partly within random errors, suggesting a systematic error among observation methods. Our results provide a new baseline for better understanding observational differences and for calibrating model ensembles[12,16,18], which will help to narrow down projections for the 21st century[11,12,18].**"

+ We added a related statement to the main text, Comparison to IPCC estimates and outlook: "Our results generally confirm glacier mass-loss trends reported by the latest IPCC reports. Over common periods, our combined estimates of global glacier mass changes are less negative (but within uncertainties) by 4%, 9%, and 8% than estimates by IPCC AR5[47], SROCC[15], and AR6[16],

respectively (Extended Data Table 3). At a regional level, the differences to the results from AR6 range between −20% for the Southern Andes and +53% for the Antarctic and Subantarctic Islands and are often within uncertainties (Extended Data Table 3).”

+ We added regional results and differences compared to IPCC AR6 to Extended Data Table 3 (here in red):

Extended Data Table 3 | Glacier mass-change rates compared to IPCC results

Source	Period	Mass change (IPCC) (Gt yr^{-1})	Mass change (this study) (Gt yr^{-1})	Δ (%)
IPCC AR5, WG1, Table 4.4, observations	2003–2009	−259 ± 28	−250 ± 27	+3%
IPCC SROCC, Table 2 A.1, observations	2006–2015	−278 ± 113	−254 ± 22	+9%
IPCC AR6, WG1, Chapter 9.5.1, observations	2000–2019	−266 ± 16	−246 ± 17	+8%
01 Alaska	2000–2019	−66.8 ± 5.2	−61.7 ± 7.0	+8%
02 Western Canada & USA	2000–2019	−7.1 ± 2.5	−7.1 ± 0.9	+0%
03 Arctic Canada North	2000–2019	−30.5 ± 2.1	−29.6 ± 2.9	+3%
04 Arctic Canada South	2000–2019	−26.6 ± 2.0	−23.2 ± 2.4	+13%
05 Greenland Periphery	2000–2019	−38.6 ± 3.6	−30.6 ± 7.2	+21%
06 Iceland	2000–2019	−9.5 ± 1.1	−8.4 ± 1.3	+11%
07 Svalbard & Jan Mayen	2000–2019	−9.2 ± 6.1	−10.7 ± 1.5	−16%
08 Scandinavia	2000–2019	−1.7 ± 0.2	−1.7 ± 0.1	+1%
09 Russian Arctic	2000–2019	−10.3 ± 1.0	−12.2 ± 2.2	−19%
10 North Asia	2000–2019	−1.2 ± 0.2	−1.3 ± 0.1	−4%
11 Central Europe	2000–2019	−1.6 ± 0.5	−1.7 ± 0.1	−6%
12 Caucasus & Middle East	2000–2019	−0.6 ± 0.1	−0.7 ± 0.1	−18%
13–15 High Mountain Asia	2000–2019	−20.0 ± 3.9	−19.0 ± 3.4	+5%
16 Low Latitudes	2000–2019	−1.1 ± 0.1	−0.9 ± 0.1	+20%
17 Southern Andes	2000–2019	−21.2 ± 6.8	−25.5 ± 6.7	−20%
18 New Zealand	2000–2019	−0.8 ± 0.1	−0.8 ± 0.1	+4%
19 Antarctic & Subantarctic Islands	2000–2019	−22.6 ± 2.7	−10.6 ± 9.0	+53%
IPCC AR6, WG1, Figure 9.21, model median	2007–2023	−347 ± 125	−300 ± 19	+16%
IPCC AR6, WG1, Table 9.SM.2, RCP 2.6	2015–2100	−333 ± 236	(2015–2023:) −323 ± 27	+3%
IPCC AR6, WG1, Table 9.SM.2, RCP 4.5	2015–2100	−502 ± 316	(2015–2023:) −323 ± 27	+36%
IPCC AR6, WG1, Table 9.SM.2, RCP 8.5	2015–2100	−670 ± 363	(2015–2023:) −323 ± 27	+52%

+ Glacier mass balance intercomparison, last paragraph: “Uncertainties originate from several sources taken as independent, including the input data's reported uncertainties, homogenization corrections, and the spread among the input data (see Methods). Our assessment does not correct systematic errors[26,32], due to the lack of reference data to perform such corrections at a regional scale. Instead, we excluded specific estimates deemed of no confidence based on expert evaluation by the GlaMBIE community (see Methods). For the same reason, we could not perform validation of our reported random errors. Many input data originate from the same sources or are obtained from similar methods and are thus likely to have correlated errors. Since true independence among these mass-change estimates is unlikely, our random errors will likely represent a lower range of the actual uncertainty for a given region. Finally, as for previous efforts, our estimates suffer from the limited knowledge of density conversion at short time scales (less than five years)[29].”

+ Differences among observation methods, last paragraph: “To further develop the applied observation methods, an intercomparison at higher spatial and temporal resolution is needed. As such, mass-change estimates at glacier (or higher) spatial resolution are required to identify the sources of observational differences[26,46]. Reference data at the glacier scale, such as from airborne laser surveys, are less prone to errors than space-borne observations and can be used for

validation. Validation of regional mass changes remains a challenge, and future efforts should be directed towards using high-quality, independent observations at multiple local sites to quantify uncertainties better and propagate these to the regional scale."

+ Comparison to IPCC estimates and outlook: "GlaMBIE extends the temporal coverage to 2023, provides results at annual resolution, and as put in place a community mechanism to allow estimates to be updated towards IPCC AR7. Regarding uncertainties, our estimates come with error bars that are smaller than the ones reported in IPCC SROCC (Extended Data Table 3, Extended Data Figure 4), which can be explained by the improvement in observational coverage. Compared to IPCC AR5 and AR6, our estimates feature similar uncertainties over multi-year periods but have the advantage to additionally provide results and uncertainties at annual resolution."

Results are calculated at the regional rather than at glacier scale which leads to some limitations. For example, the authors state in the abstract that 'Our results provide a new observational baseline for calibrating model ensembles'. However, most model calibration occurs at glacier scale. E.g. in glacier models (e.g., GloGEM, OGGM, PyGEM and so on), each glacier is simulated separately, and the surface mass balance parameters are also calibrated at single glacier scale. This widely accepted approach ensures the accuracy of global projections. Thus, a global glacier mass change dataset like Hugonnet et al. (2021; https://www.nature.com/articles/s41586-021-03436-z) is more readily useful for model calibration.

Response: Indeed, the glaciological method and DEM differencing can deliver results at the glacier scale, while current estimates from altimetry and gravimetry mainly provide regional estimates, and this is only for regions with a large glacier area. It is true that currently, most glacier models use the results from Hugonnet et al. (2021) for calibration at the glacier scale. However, users need to be aware of the method-dependent limitations of that dataset: while DEM differencing from ASTER can provide long-term trends of glacier elevation changes that are reliable on regional average, it can also be affected by large uncertainties for smaller glaciers and in regions with larger data voids (e.g. due to frequent clouds). Also, DEM-differencing cannot capture the inter-annual or seasonal variability well. GlaMBIE provides an ensemble of results that opens new ways to calibrate and validate glacier models at multiple spatio-temporal scales.

We agree that future research should aim for intercomparisons at higher spatial and temporal resolutions. We clarified the rationale for regional resolution and provided an outlook in the main text:

+ Glacier mass balance intercomparison: "These estimates are based on one or more of the four observation methods (glaciological, DEM differencing, altimetry, gravimetry) and cover the period since the early 2000s. While the glaciological method and DEM differencing can provide results for individual glaciers with annual and decadal resolution, respectively, current estimates from altimetry and gravimetry are only available on a regional scale but can provide monthly variations. Similarly, DEM differencing and altimetry observe elevation changes, while the glaciological method and gravimetry provide mass changes. To account for the strengths and

limitations of the different methods, we collected all regional datasets in the native units (i.e., metre, metre water equivalent, gigatonnes) and at the temporal resolutions specified by the participants (i.e., monthly, annual, multi-annual)."

+ Differences among observation methods: "To further develop the applied observation methods, an intercomparison at higher spatial and temporal resolution is needed. As such, mass-change estimates at glacier (or higher) spatial resolution are required to identify the sources of observational differences[26,46]."

B. Originality and significance: if not novel, please include reference

The results are original but a current preprint (Dussaillant et al. (2024) https://essd.copernicus.org/preprints/essd-2024-323/) shares many of the authors and a somewhat similar approach – a blending of glacier mass balance observations with remotely sensed surface elevation datasets. However, in Dussaillant et al. (2024), mass changes are resolved at individual glacier scale (which I believe is an improvement). What are the relationships and dependencies between these two papers and datasets?

Response: As described in the Methods section, "Dussaillant et al.[56,98] and Huss et al.[23] combined glaciological observations with geodetic estimates at the glacier scale for all 19 regions with similar but partly different approaches. From their results, we assigned the temporal variability to the glaciological and the long-term trends to the DEM differencing methods."

These two datasets build the backbone of the GlaMBIE estimates in regions with a small glacier area but are complemented with additional estimates from altimetry and gravimetry in regions with a larger glacier area. Dussaillant et al. and Huss et al. make 38 out of 195 regional input datasets to compute our combined estimates (Extended Data Figure 1, Supplementary Data Table 1).

The overall findings at global scale (glacier mass loss of 273±16 gigatonnes per year from 2000 to 2023) reported in this submission are very similar (-267 ± 16 gigatonnes per year in 2000-2019) to those reported by Hugonnet et al., 2021. The years do not exactly overlap and the authors point out the real difference is 16%, but given the absolute uncertainties are relatively large (at face value between 5-10%, and possibly larger due to processes not considered) the updated mass loss estimate is not a hugely new finding for a general readership. The identified acceleration in mass loss over the last decade was also reported by Hugonnet et al., 2021.

For example, on Line 196 - 'Our results generally confirm glacier mass-loss trends reported by the latest IPCC reports.'

The results in this paper are certainly more comprehensive but the basic pattern and magnitude of mass loss is similar to previous work.

The findings are extremely important. However I question whether they are novel.

Response: Our study is novel since it is the first inter-comparison exercise that provides the methodology to combine the results from the main observation methods into an annual time series for all glacier regions. As such, the GlaMBIE framework and methodology will allow us to regularly update our estimates based on new observations from methods with high temporal resolutions (e.g., glaciological observations, altimetry, and gravimetry).

Given the upcoming IPCC AR7, we believe it is of great relevance to validate the previous state of knowledge based on a much larger community-based observational baseline. It is good news that our results confirm earlier findings by Hugonnet et al. (2021) and IPCC AR6 (mainly based on Hugonnet et al. 2021) at a global scale and within error bars. However, we find larger differences at a regional scale, partly within random errors, suggesting systematic differences between observation methods.

As shown in the response to point A (above), we revised the summary paragraph and main text to emphasize the improvements, and we added a regional comparison to the results of IPCC AR6 to Extended Data Table 3. In addition, we summarized the novelty of our study in the main text, Global and regional mass changes, last (additional) paragraph:

"GlaMBIE has developed the framework and methodology to compile and update mass-change estimates based on the main in situ and satellite observations. As such, we extended the observation period in all glacier regions from 2000 to 2023 and provided an annual resolution. This offers a new observational baseline for future studies to better understand climate change's impact on glaciers and the associated downstream effects at the regional scale. While the current annual resolution estimates are sufficient to assess the contribution of glaciers to sea-level rise, a monthly (or higher) temporal resolution is needed to quantify the contribution of glaciers to the hydrological cycle[6]."

C. Data & methodology: validity of approach, quality of data, quality of presentation

My impression is that in the new dataset, glaciological observations dominate the annual variability and DEM differencing dominates the long-term trend. P11 L472~L474 suggests this - "In cases without annual observations (e.g., altimetry pre-2010 or gravimetry during the observational gap between GRACE and GRACE-FO), we used the averaged anomaly from the other observation methods to fill in βy for missing years.' This may be why the overall trends are very similar to Hugonnet et al. (2021) and Dussaillant et al. (2024).

Response: This depends on the region and the availability of observation methods. "we used the average anomaly from the other observation methods in $\bar{\beta}_y$ for missing years." [e.g., Hugonnet et al. (2021) and Dussaillant et al. (2024)] "This yearly time series was then re-trended by adding the long-term trend of each input dataset." [e.g., altimetry or gravimetry]. Hence, the overall trends are from the original methods and the anomalies are bias-corrected over common observation periods. We show the comparison to IPCC AR6 with regional differences between −20 and +53% in the updated Extended Data Table 3 (see above).

This leads me to a question about the annual variability. Is extrapolating individual mass balance observations to regional scale ever valid? Let me explain in more detail.
- Glacier mass balance measurements are unique to each glacier.
- Mass balance programmes are nearly always carried out on glaciers that (for example) are lower in elevation, flatter and more accessible and which may very well not reflect the region-wide variability.
- The strong spatial correlation between glacier mass balance over several hundred kilometres doesn't mean that individual glaciers have a similar mass change to the aggregated trend in each RGI region.

Response: Agreed. As explained in the main text and the Methods section, the annual variability is well correlated regionally, whereas the long-term trend of individual glaciers might not be representative of their entire region. This is why we separated and recombined the temporal variability from the long-term trend for each dataset. The reasoning is explained in the Methods, Glaciological observations, and implemented in the Methods, Separation of temporal variability from the long-term trend.

It's easy to understand how to extract regional values based on DEM differencing, altimetry and gravimetry. However, how to calculate the regional value from a few and unevenly distributed glaciological observations remains unclear. I would like the authors to explain this further.

Response: We added a short paragraph to the main text, Glacier mass balance intercomparison, to better explain the strengths and limitations of the observation methods: "While the glaciological method and DEM differencing can provide results for individual glaciers with annual and decadal resolution, respectively, current estimates from altimetry and gravimetry are only available on a regional scale but can provide monthly variations. Similarly, DEM differencing and altimetry observe elevation changes, while the glaciological method and gravimetry provide mass changes."

In the same section, we summarized our approach, "…, we combined the selected estimates using a five-step approach (see Methods and Extended Data Figure 2): First, we homogenized all datasets concerning spatial, temporal, and unit domains using common conversions. Second, we separated the temporal variability from the long-term trend for each dataset. Third, we combined each region's average temporal variability and long-term trends. Depending on available input data, we computed three regional time series: one for altimetry, one for gravimetry, and one combining the temporal variability from the glaciological data with the long-term trends from DEM differencing. Fourth, we combined the time series from altimetry and gravimetry and the combination of glaciological and DEM differencing into single regional estimates. Fifth, we summed the regional estimates to a global time series."

Note that we did not use the long-term trend from glaciological observations, but we combined their inter-annual variability with the long-term trend from DEM differencing. Hence, we did not calculate a regional value by simply extrapolating a few glaciological observations. To further clarify the influence of input data and observation methods, we revised and extended the corresponding Methods section at the end of Quality control, homogenization, and selection:

"Among observation methods, the weight of a given method depends on the availability of regional data and the assessed confidence level. As an example, the glaciological observations have no weight on regional trends (due to 'no confidence') but determine the temporal variability by one-third in Iceland (sharing the same weight with altimetry and gravimetry) and fully in Central Europe (due to lack of confident results from the other methods)."

The approach is further detailed, including supporting equations in the Methods section, illustrated in Extended Data Figure 2, and provided in full detail in the GlaMBIE code.

Following the editor's suggestion, we also revised the introduction of the Methods section to provide a summary of the method in plain language and better links to where more details are available (see above).

Related to this (top of Page 5): Given that glaciological measurements are shown to have a bias, do the authors have any recommendations on the future of direct mass balance programmes?

Response: The strength of glaciological in-situ observations is that they capture the seasonal and annual variability of glacier surface balance, with time series extending back into the early 20th century. These observations are precious for process understanding and model calibration/validation. Given the strengths and limitations of the remote sensing methods, we need to maintain long-term glaciological monitoring programs and expand them into under-sampled regions (e.g., Andes, High Mountain Asia, Arctic, Antarctic). At the same time, we need to push these in-situ networks towards continuous and near-time monitoring. These recommendations have already been made in earlier publications (e.g., Zemp et al. 2015, WGMS 2023) and – complemented with recommendations for spaceborne methods – would go beyond the scope of the present publication. However, in the context of GlaMBIE, we are in preparation for an opinion article in the EOS science magazine (https://eos.org/) with recommendations for in-situ and remote sensing methods for glacier mass-change monitoring. We aim to submit this article as a follow-up to the present paper once it is accepted for publication.

Further and more specific comments:

Page 3 – 'However, mass loss due to calving front retreat of marine and lake-terminating glaciers is not included since that does not significantly impact global sea level.'

Comment: this is true if ice is floating (for the component of the glacier above floatation) but many such lake and fiord glaciers are grounded or partially grounded, and I am not sure this statement is correct. Do the authors have evidence to support it? What proportion of global glaciers are in this category? I note the largest mass loss reported in this study is from Greenland, Alaska and Patagonia where calving glaciers are common.

Response: This sentence needs to be read in the context of the entire paragraph. We differentiate between glacier calving due to ice discharge and calving front retreat. Calving due to ice discharge of the whole (mostly) grounded glacier is implicitly accounted for in the observed elevation changes in DEM differencing and altimetry, and in the mass-change signal in gravimetry. However, the retreat of calving fronts is not captured in the observations of most

input data. We do provide an estimate of the mass loss due to calving front retreat for Northern Hemisphere glaciers based on Kochtitzky et al. (2022)[27]:

Main text, Global and regional mass change: "We note that our mass-change estimates include glacier calving due to ice discharge, which is implicitly accounted for in DEM differencing, altimetry, and gravimetry. However, mass loss due to calving front retreat of marine and lake-terminating glaciers[34,35] is not included since that does not significantly impact global sea level. For the last two decades, mass loss due to calving front retreat was quantified to 10±3 Gt yr^{-1} for Northern Hemisphere glaciers[34] and 43 Gt yr^{-1} for the Greenland Ice Sheet[36], for comparison."

Methods section, DEM differencing: "DEM differencing represents glacier mass changes above sea level as it implicitly accounts for calving due to ice discharge (contributing to sea-level rise), but it does not include any mass changes below water level (not contributing to sea-level rise) due to the retreat or advance of lake- and ocean-terminating glaciers[34]."

Methods section, Altimetry: "Similar to DEM differencing, altimetry represents glacier mass changes relevant to sea-level rise."

Page 3 – Line 132. 'This increase is concurrent with the general pattern of atmospheric warming, overlaid by regional decadal variability9,26,49'.
- The authors need to make it clear that the examples provided below this sentence (e.g. end of Karakoram-Kunlun anomaly, slowdown of Icelandic mass loss) follow on from this sentence.

Response: We added "as such, " to better link the general statement with the following examples: "…; this increase is concurrent with the general pattern of atmospheric warming, overlaid by regional decadal variability[11,27,37]. As such, we note that the regions with an increase in mass loss include South Asia West, …"

- I'd like to see further discussion of these regional results. Methodological differences etc (the next section) are important for the glaciological community but are of less general interest.

Response: Within the scope of the present publication, we aim to highlight the potential of the GlaMBIE results for future research on (a) regional interpretation in the context of atmospheric trends and variability and (b) on better understanding and reducing the differences within and among methods. While (a) certainly is of general interest, (b) is also relevant for all remote sensing communities of related sensors. We added a statement at the end of both sections to invite the communities to use GlaMBIE results for future research in both areas.

Main text, Global and regional mass changes: "GlaMBIE has developed the framework and methodology to compile and update mass-change estimates based on the main in situ and satellite observations. As such, we extended the observation period in all glacier regions from 2000 to 2023 and provided an annual resolution. This offers a new observational baseline for future studies to better understand climate change's impact on glaciers and the associated downstream effects at the regional scale. While the current annual resolution estimates are sufficient to assess the contribution of glaciers to sea-level rise, a monthly (or higher) temporal resolution is needed to quantify the contribution of glaciers to the hydrological cycle[6]."

Main text, Difference among observation methods: "To further develop the applied observation methods, an intercomparison at higher spatial and temporal resolution is needed. As such, mass-change estimates at glacier (or higher) spatial resolution are required to identify the sources of observational differences[26,46]. Reference data at the glacier scale, such as from airborne laser surveys, are less prone to errors than space-borne observations and can be used for validation. Validation of regional mass changes remains a challenge, and future efforts should be directed towards using high-quality, independent observations at multiple local sites to quantify uncertainties better and propagate these to the regional scale."

D. Appropriate use of statistics and treatment of uncertainties
The quality of the data processing and presentation is excellent I very much appreciate the community vetting process.

Response: Thanks.

Note that P1 L27: "32±8 mm" should be "320±80 mm" or 32 cm.

Response: Thanks for spotting this error, which was introduced when aligning units to mm sea-level rise! We corrected the value to the correct digits based on Farinotti et al. 2019, Table 1: "… equivalent to a potential sea-level rise of 324±84 mm[20]."

E. Conclusions: robustness, validity, reliability
I believe that the conclusions of this paper are robust at the broad scale. However, I'm not sure that they offer sufficient novelty compared to previous work or have sufficiently reduced uncertainty for them to become a new benchmark.

Response: As outlined in the responses above, we updated the summary paragraph to highlight the advances and relevance of the present work. We also added a corresponding overview of regional differences to IPCC AR6 to Extended Data Table 3 and a statement on our reported uncertainties to the main text (see above).

In addition, we added two paragraphs to discuss the limitations and perspectives for future improvements of uncertainty estimates:

+ Glacier mass balance intercomparison, last paragraph: "Uncertainties originate from several sources taken as independent, including the input data's reported uncertainties, homogenization corrections, and the spread among the input data (see Methods). Our assessment does not correct systematic errors[26,32], due to the lack of reference data to perform such corrections at a regional scale. Instead, we excluded specific estimates deemed of no confidence based on expert evaluation by the GlaMBIE community (see Methods). For the same reason, we could not perform validation of our reported random errors. Many input data originate from the same sources or are obtained from similar methods and are thus likely to have correlated errors. Since true independence among these mass-change estimates is unlikely, our random errors will likely represent a lower range of the actual uncertainty for a given region. Finally, as for previous

efforts, our estimates suffer from the limited knowledge of density conversion at short time scales (less than five years)[29].” (Note that in contrast to previous efforts, we accounted for the density conversion issue by increasing the error bar with shorter time scales, as described in the Methods section, Quality control, homogenization, and selection.)

+ Differences among observation methods, last paragraph: “To further develop the applied observation methods, an intercomparison at higher spatial and temporal resolution is needed. As such, mass-change estimates at glacier (or higher) spatial resolution are required to identify the sources of observational differences[26,46]. Reference data at the glacier scale, such as from airborne laser surveys, are less prone to errors than space-borne observations and can be used for validation. Validation of regional mass changes remains a challenge, and future efforts should be directed towards using high-quality, independent observations at multiple local sites to quantify uncertainties better and propagate these to the regional scale.”

F. Suggested improvements: experiments, data for possible revision
It might be more convincing to compile different glacier mass change datasets at single glacier scale and then to regional scale rather than based on 19 first-order glacier regions directly.

Response: We revised the main text to explain better the rationale for an intercomparison at a regional scale and added an outlook for future research:

Main text, Glacier mass balance intercomparison: “These estimates are based on one or more of the four observation methods (glaciological, DEM differencing, altimetry, gravimetry) and cover the period since the early 2000s. While the glaciological method and DEM differencing can provide results for individual glaciers with annual and decadal resolution, respectively, current estimates from altimetry and gravimetry are only available on a regional scale but can provide monthly variations. Similarly, DEM differencing and altimetry observe elevation changes, while the glaciological method and gravimetry provide mass changes. To account for the strengths and limitations of the different methods, we collected all regional datasets in the native units (i.e., metre, metre water equivalent, gigatonnes) and at the temporal resolutions specified by the participants (i.e., monthly, annual, multi-annual).”

Main text, Differences among observation methods: “To further develop the applied observation methods, an intercomparison at higher spatial and temporal resolution is needed. As such, mass-change estimates at glacier (or higher) spatial resolution are required to identify the sources of observational differences[26,46]. Reference data at the glacier scale, such as from airborne laser surveys, are less prone to errors than space-borne observations and can be used for validation. Validation of regional mass changes remains a challenge, and future efforts should be directed towards using high-quality, independent observations at multiple local sites to quantify uncertainties better and propagate these to the regional scale.”

We are currently negotiating with ESA for a follow-up phase of GlaMBIE, including a pilot study at higher spatial and temporal resolution. Fingers crossed. 😊

G. References: appropriate credit to previous work?

No issues

Response: Thanks. Following the editor's suggestion, we updated the Methods section to include all method references listed in the Supplementary material for proper credit and indexing.

Clarity and context: lucidity of abstract/summary, appropriateness of abstract, introduction and conclusions

Abstract: Note that glacier retreat also impacts biodiversity and not just sea level, hazards and water resources. The authors should cite https://www.nature.com/articles/s41586-024-07778-2

Response: We added a short statement and references to the impact on marine and terrestrial ecosystems (Hopwood et al. 2020, The Cryosphere; Ficetola et al. 2024, Nature; Bosson et al. 2023, Nature) to the summary paragraph:

"Glaciers are icons of ongoing anthropogenic climate change[1]. Their melting leads to increased local geohazards[2], impacts marine[3] and terrestrial[4,5] ecosystems, regional freshwater resources[6], and the global water and energy cycles[7,8]."

The paper is generally well written but would benefit from English language editing.

Response: We polished the English across the paper with the support of our native co-authors.

See further minor comments below:
Page 1 - Line 22 – use non gendered language.

Response: We deleted this sentence to shorten the summary paragraph.

Page 2 – Line 67. Does this mean the 7th IPCC Assessment report. Please specify.

Response: Yes. We clarified the statement: "… towards the upcoming IPCC reports of the seventh assessment cycle."

Page 2 – Line 76. Change 'and cover periods since 2000' to 'and covers the time period since the early 2000s'.

Response: Done: "These estimates are based on one or more of the four observation methods (glaciological, DEM differencing, altimetry, gravimetry) and cover the period since the early 2000s."

For regional glacier area and related changes over time, we assume a general uncertainty of ±5%68.
-What is the justification for this?

Response: We clarified the text providing reference to the intercomparison study by Paul et al. (2017): "For regional glacier area and related changes over time, we assume a general uncertainty of ±5% based on a glacier mapping intercomparison study[57]."

How is the influence of changing glacier area (as glaciers retreat) on mass balance considered? Or does this not matter because of the way that mass balance observations influence variability rather than trends?

Response: The influence of changing glaciers does matter when calculating specific mass changes (m w.e.) but less (depending on the observation method used) when calculating mass changes in Gt. GlaMBIE provides results in both m w.e. and Gt and, hence, does consider glacier area change at a regional scale as described in the Methods section, Glacier area, volume and mass in 2000:

"To account for glacier shrinkage, we used regional glacier area-change rates (% per year) compiled from IPCC AR5[47] (Chapter 4, figure 4.10 and Table 4.SM1), extended with additional literature by Zemp et al. (2019)[25]. With these annual area-change rates, we corrected glacier area from RGI 6.0 to the year 2000 (Table 1) and computed yearly time series (t_y) of regional glacier area (S in km^2):

$$S_{t_y} = S_{t_0} + (t_y - t_0) \cdot \delta S/\delta t \cdot S_{t_0}$$

where S_{t_0} is the regional glacier area in the (area-weighted average) reference year t_0 and $\delta S/\delta t$ the annual area-change rate (in percentage)."

++

Additional improvements

During the revision of the manuscript, we implemented the following additional improvements, listed here for completeness and transparency:

Updated Team list: In the last version of the manuscript, we forgot to add one of the data providers and co-authors – Johan Nilsson, Jet Propulsion Laboratory, California Institute of Technology, Pasadena, US – to The GlaMBIE Team. We added him to the team list in the revised version and updated the Author Contribution Statement and the CReDIT table in the supplementary material accordingly.

Additional internal reviews: One of our co-authors' home institutions, the U.S. Geological Survey (USGS), has internal regulations that require additional internal reviews. We received feedback from Brianna Rick (University of Alaska Fairbanks) and Greg Pederson (USGS). We added their comments and our responses for completeness below. In the acknowledgements, we thanked them "for their feedback on the manuscript".

Feedback from USGS: Based on the feedback from both internal reviewers and our responses, the USGS Bureau Approving Officer (BAO) granted approval contingent on the following changes to the manuscript:

+ The affiliation of USGS co-authors (line 860) are changed to simply "U.S. Geological Survey."

Response: Done.

+ The last sentence of the Abstract is deleted: "Our study reminds us of the urgent need for tangible measures to prevent further human-made climate change and limit the impacts of glacier vanishing." Rationale from the BAO: A USGS scientist cannot advocate for policy change and cannot issue urgent calls for tangible measures; this sentence clearly crosses the line.

Response: We deleted the related sentence from the summary paragraph.

+ The same sentence in lines 283-286 is deleted. Rationale from the BAO: It's advocacy and USGS cannot advocate. USGS scientists cannot co-author a product with these sentences.

Response: To avoid advocacy, we revised the corresponding sentence to:

"Altogether, our observations and recent modelling studies[11,18] indicate higher projected glacier mass loss than the estimates from IPCC AR6[12,16]. We are, therefore, facing continued and possibly accelerated mass loss until the end of this century. This underpins the IPCC's call[16] for urgent and concrete actions to reduce greenhouse gas emissions and associated warming to limit the impact of glacier down-wasting on local geohazards, regional freshwater availability, and global sea-level rise."

Feedback from Brianna Rick (University of Alaska Fairbanks): "The manuscript entitled "Community estimate of global glacier mass changes from 2000 to 2023" by The GlaMBIE Team addresses the issue of global estimates of glacier mass change. In the paper, the authors present the results of combining in-situ and remote sensing-based glacier mass balance observations at regional to global scales. These data are used to estimate global glacier mass change rate, and to compare to previous estimates, which aids in narrowing projections of glacier mass loss and sea level rise for the 21st century. The authors find a global glacier mass-change rate of -273±16 gigatonnes per year from 2000 to 2023. This rate increased by 36±10% between the time periods of 2000-2011 to 2012-2023. Results confirm the glacier mass loss trends reported in recent IPCC reports, and are within the uncertainty for other glacier mass change estimates, though are slightly less negative. These estimates are important for future glacier mass loss projections, as well as estimates of sea level rise. The authors do a good job of recognizing and quantifying the uncertainty of their methods and estimates, and communicate their findings in an approachable way. Overall, I find this article suitable for submission."

Response: We thank Brianna Rick for the feedback.

Feedback from Greg Pederson (USGS): Thank you for the opportunity to review your manuscript entitled "Community estimate of global glacier mass changes from 2000 to 2023". It

was a compelling, though thoroughly depressing read, on the current best estimate of glacier mass loss regionally and globally over recent decades. The manuscript is generally well-written, the figures are of exceptional quality, and the results should be of great interest to the public and all scientists working on issues related to glaciers and the cryosphere. To the best of my understanding, the methods employed in conducting the study represent a novel, uniform, and highly rigorous advancement over previous methods used in regional studies and in all prior IPCC estimates and forecasts. Correspondingly the results appear highly robust at both regional and global scales, and importantly support the central conclusions made in the paper. For these reasons, I find the manuscript suitable for publication and I think it will be well received by the global scientific community.

Response: We thank Greg Pederson for the feedback.

Though the manuscript as written is succinct and well explained, and could be published in its current form, I do have a few minor comments to consider before resubmitting the paper to the journal. Some little comments are provided as highlighted call-outs in the manuscript PDF [added below]. Please use or disregard the suggestions at your discretion.

Main comment:
In both the introduction and discussion, it's stated that the methods this team of glaciologists has advanced stands to improve mass-balance estimates regionally and globally, but in many cases, those advances appear understated. Especially when you look at how small the differences are between these revised mass-loss estimates from the GlaMBIE team relative to the last IPCC estimates. Consider explaining in greater detail, or better highlighting, the specific benefits this method will provide in future glacier mass estimates, and what this means at regional and global scales.
Response: This feedback aligns with the feedback of the editor and other referees. To better highlight the advance of our study, we made the following improvements:

+ We revised the first paragraph: "**While our results generally confirm the findings from previous assessments[14–16] at a global scale, we found some large regional differences, partly within random errors, suggesting a systematic error among observation methods. Our results provide a new baseline for better understanding observational differences and for calibrating model ensembles[12,16,18], which will help to narrow down projections for the 21st century[11,12,18].**"

+ We added a related statement to the main text, Comparison to IPCC estimates and outlook: "Our results generally confirm glacier mass-loss trends reported by the latest IPCC reports. Over common periods, our combined estimates of global glacier mass changes are less negative (but within uncertainties) by 4%, 9%, and 8% than estimates by IPCC AR5[47], SROCC[15], and AR6[16], respectively (Extended Data Table 3). At a regional level, the differences to the results from AR6 range between −20% for the Southern Andes and +53% for the Antarctic and Subantarctic Islands and are often within uncertainties (Extended Data Table 3)."

+ We also added regional results and differences compared to IPCC AR6 to Extended Data Table 3 (see above).

Correspondingly, modestly expanding the discussion around both the regional and global estimates and projections of future glacier mass loss may be helpful. I'm impressed by how similar the most regional estimated glacier mass losses are relative to regional IPCC projections in supplemental figures 20 and 21, but is this type of comparison valid when considering uncertainties in climate model ensembles and regional glacier types, glacier model specifics, or in light of regional decadal climate variability? Conversely, what lessons could be learned in making such a comparison?

Response: Our comparison of the observations with model projections summarises the conclusions from several discussions with several co-authors from the glacier modelling community (e.g., M.H., F.M., L.S., H.Z.). The comparison, as shown for global results (Extended Data Figure 5) and regional results (Supplementary Data Figures 21 and 22), is certainly just a starting point and deserves to be addressed in future research bringing together both the observation and modelling communities.

I recognize space is limited, so any minor additional expansion around these points may be helpful to the reader. Otherwise, I look forward to seeing this impressive collaborative effort on a global glacier assessment published.

Response: Given the limited space, we focused our improvements on better explaining the advance of our study.

Line 27-28: This may be a translation or unfamiliar reference issue, but aren't glaciers more of a "climate-change index" than they are a "climate variable". When I hear climate variable I think of atmospheric variables (e.g. temp, precip,, humiditiy), whereas glaciers change in response to these variables, hence and index.

Response: The concept of "Essential Climate Variable" has been introduced by the Global Climate Observing System and was adopted by IPCC. In this sentence, we use this term in line with the corresponding reference (Bojinski et al. 2014, BAMS): "Glaciers have long been considered essential climate variables[1]."

Line 74: Was this supposed to be "Supplemental Data Table 1?"

Response: We revised the sentence to refer to both Extended Data Table 1 (i.e., Sankey diagram to visualise the GlaMBIE) and Supplementary Data Table 1 (listing all data submission with more detail): "For 19 predefined regions, we compiled 233 estimates of regional glacier mass changes from about 450 data contributors organised in 35 research teams (Extended Data Figure 1, Supplementary Data Table 1)."

Lines 89-91: This is a major methodological accomplishment and advancement in the science. Hopefully this has been highlighted throughout the manuscript.

Response: Thank you.

We highlight this point in the summary paragraph:

"Previous assessments of global glacier mass changes were hampered by spatial and temporal limitations and the heterogeneity of existing data series[14–16]. The Glacier Mass Balance Intercomparison Exercise (GlaMBIE) tackles these challenges through a joint effort across the scientific community to collect, homogenize, combine, and analyse glacier mass changes from in situ and remote-sensing observations at regional to global scales."

And we revised the introduction of the Methods section to explain our approach in plain language:

"In total, we analysed 233 regional estimates of glacier mass changes from about 450 data contributors organized in 35 research teams (Supplementary Data Table 1). Data contributions were selected based on expert evaluation of their confidence levels. Within each observation method, the selected input data were homogenized for time, space, and unit domains using common corrections. They were then combined first within and second among methods for each of the 19 regions and finally aggregated to global estimates. Below, we briefly summarize the input data (Extended Data Figure 1) and the general workflow (Extended Data Figure 2) of our intercomparison exercise, including key equations. The full methodological details are available in our code (Code availability). For more information on the different observation methods, we refer to a recent review on measuring glacier mass changes from space[26] and the methods references of our input data as listed in Supplement Data Table 1. As an output, GlaMBIE provides the native input data in a standardized format, combined estimates per observation method, and combined estimates among observation methods (Data availability). Given the available data, we consider our combined estimate (among methods) to best reflect the expert evaluation of the GlaMBIE community."

Line 161: The extended data figures citend in the main text are not aligning with the supplemental figures I received. Please ensure the cited supplemental figures and tables in the main text align with the supplemental section to be resubmitted to the journal. Nevermind, I now see the extended data table and figure section is different from the supplemental. Disregard my notes on fixing this.

Response: Yes, Nature differentiates between Extended Data, which comes with the main article in the online version (but not in the printed article), and Supplementary Data, which comes as a PDF file linked in the online article.

Lines 196-215: Can you discuss in a bit more detail, what other aspects of your methodological improvements will mean for future reporting on glacier mass loss in the IPCC reports? A few sentences really drawing out the strength of the methods here could help round out this paragraph a little more.

Response: We added the following sentences to the end of this section:

“Regarding uncertainties, our estimates come with error bars that are smaller than the ones reported in IPCC SROCC (Extended Data Table 3, Extended Data Figure 4), which the improvement in observational coverage can explain. Compared to IPCC AR5 and AR6, our estimates feature similar uncertainties over multi-year periods but additionally have the advantage of providing results and uncertainties at annual resolution.”

Lines 225-228: This sounds like regional decadal climate variability, which is not well captured in ensembles of climate model runs.

Response: We considered this comment and concluded that it underscores the closing statement included in this sentence: “, which calls for further investigations.” Future research needs to test hypotheses like this, i.e. regional decadal variability, in a similar way as the work by Malles & Marzeion (2021), cited in the following sentences: “Remarkable deviations between model ensemble and observations were already identified in these regions by a recent study with one glacier model[50]. They were attributed to the lack of calibration data (Russian Arctic), a negative bias in glaciological observations used for model calibration (Central Asia, South Asia West), issues in model setup forced with reanalysis data (Southern Andes), and large portions of marine-terminating glaciers not well represented in the model (Southern Andes).”

Line 824: Glad to see this here.

Response: Thanks. See also comment on USGS disclaimers below.

Line 865: Wonderful figure! It shows an incredible amount of data succinctly.

Response: Thanks.

Line 918: Impressive workflow here. This was a major effort and the quality of the results show in your figures.

Response: Thanks. Based on feedback from other referees, we further improved the readability of Extended Data Figure 1 and the related caption. (see above).

Best regards,
Greg Pederson

Disclaimer: USGS internal regulations required the following institutional disclaimers, which were added to our manuscript:

+ Acknowledgements: “Any use of trade, firm, or product names is for descriptive purposes only and does not imply endorsement by the U.S. Government or by any other of the co-author’s institutions.”

+ Additional information [This disclaimer refers to the review process and can be deleted in the final article.]: “**USGS disclaimer**. This draft manuscript is distributed solely for purposes of

courtesy review and comments received will be addressed and treated as appropriate to ensure there is no conflict of interest. Its content is deliberative and predecisional, so it must not be disclosed or released by reviewers. Because the manuscript has not yet been approved for publication by the U.S. Geological Survey (USGS), it does not represent any official USGS finding or policy."

Additional minor changes: Based on co-author feedback during the revision, we made some minor changes to the manuscript (mainly rewording) to improve clarity and readability. All changes are reported in the track-change document.

Community estimate of global glacier mass changes from 2000 to 2023

The GlaMBIE Team

Corresponding author: Michael Zemp, michael.zemp@geo.uzh.ch

Author’s response related to the comments received to the manuscript 2024-07-14479A

We thank the referees and the editor for their feedback in the second review. After considering all their comments and suggestions, we revised our manuscript, improving our paper's clarity.

The main changes we made to the manuscript are as follows:

- We revised the summary paragraph based on the feedback from the editor and the referees.
- We double-checked and revised – where necessary – our manuscript based on the feedback from the editor and the two referees.
- We edited the English of our manuscript with the support of our native English-speaking co-authors.
- We separated the information in SI Table 1 into two new SI Tables 1 and 2 for better readability.

Below, we outline our revisions in a point-by-point reply. There are two points that require further guidance by the editor:

- Cover artwork: we provide a suggestion for a cover artwork (see below). In addition, ESA offered support to generate a rendering of a glacier landscape with overflying satellites. Please let us know if the timeline towards publication would allow to work on such an additional suggestion for a cover artwork.
- Licence agreement: One of our co-authors is employee of the U.S. Geological Survey, which requests a separate licence agreement (see below). Please let us know if such a separate agreement is feasible.

Point-by-point reply to reviewer comments: editor and reviewer comments are in black, and the author’s responses are in blue, with citations from the revised manuscript in green.

+ +

Comments by Editor, 27th August 2024

11th November 2024

Dear Michael,

Your revised manuscript, "Community estimate of global glacier mass changes from 2000 to 2023", has now been seen by the original referees 2 and 3, whose comments are attached below. In the light of their advice I am delighted to say that we can in principle offer to publish it. First, however, we will need you to revise your paper to address the points made by the referees, and to

make some editorial changes to your paper so that it is as brief as possible and complies with our Guide to Authors (https://www.nature.com/nature/for-authors).

Response: We thank the referees for their feedback and the Editor for his guidance, which greatly improved our manuscript. We are excited to see the results of GlaMBIE being published in Nature.

I'll begin with a few comments on the referees' reports. On referee 2's five points, our guidance is as follows:

Response: We highly appreciate the Editor's guidance on implementing the referees' main points.

1) You are welcome to further revise ED figure 1, but this is not required.

Response: We decided to keep ED Figure 1 unchanged. See also the response to Referee #2 below.

2) If you see a clear way to simplify SI table 1 you are welcome to do so.

Response: We decided to split SI Table 1 into two. Newly, we have SI Table 1, which provides an overview of methods, sensors or products, method references, and regions for each research team, and SI Table 2, which provides the affiliations for each research team. See also the response to Referee #2 below.

3) If the requested time spans are available elsewhere you do not need to add them to SI table 1.

Response: Providing information about temporal coverage, resolution, and gaps for each research team and region in SI Table 1 is not feasible. However, we added a sentence to the caption of SI Table 1, pointing out that "Information about temporal coverage, resolution, and gaps of the submitted data are shown for each region in SI Figures 1−19.". See also the response to Referee #2 below.

4) If you see a straightforward way to extend your time series or add additional time series you are welcome to do so. Again, doing so is not required.

Response: GlaMBIE is a community effort that has compiled observations through an open call for data. Extending or adding additional time series at this stage is not feasible. To clarify this point, we added a corresponding sentence in the first paragraph of the Methods section: "Data contributions were compiled through an open call for data submission and selected based on expert evaluation of their confidence levels."

We are confident that the spatial and temporal coverage for all methods will be further improved in a future phase of GlaMBIE.

See also the response to Referee #2 below.

5) Please harmonize your color schemes where appropriate.

Response: SI Figures 1−19 come with consistent colors and formats. As noted in the caption ("Description of Supplementary Data Figures 1−19"), "datasets not used are shown as grey dotted lines".

Please implement referee 3's suggestions for more guarded wording.

Response: We agree that more guarded wording can help to present the results in the right context. At the same time, we partly disagree with the referee's comments suggesting that differences within uncertainty bounds are meaningless. However, we double-checked and revised our corresponding text under consideration of the suggestions of Referee #3, as outlined below.

Next there are a few remaining specific requests. As before please provide a point-by-point reply to each.

-Change "Supplementary Data" to "Supplementary Information" throughout.

Response: Done.

-Wording on line 968-970 suggests that the SI will be moved to a repository. But as the SI does not contain data, please continue to submit it as a PDF and do not move to a repository.

Response: Understood. We submitted the SI as PDF.

-ED tables should be submitted as stand-alone items, just like the ED figures.

Response: Understood. We submitted the ED Tables as stand-alone JPG files.

-Remove main text figures and all ED items (figures and tables) from the article file, submitting them only as stand-alone items. But keep the main text tables in the article file.

Response: Done.

-The bold-faced summary paragraph appears to exceed our limit of 230 words. A judicious edit should suffice.

Response: We revised the summary paragraph to improve its clarity, to polish the English, and to fit the limit of 230 words:

"Glaciers are icons of ongoing anthropogenic climate change[1]. Their melting leads to increased local geohazards[2], impacts marine[3] and terrestrial[4,5] ecosystems, regional freshwater resources[6], and both global water and energy cycles[7,8]. Together with the Greenland and Antarctic ice sheets, glaciers are essential drivers of present[9,10] and future[11–13] sea-level rise. Previous assessments of global glacier mass changes were hampered by

spatial and temporal limitations and the heterogeneity of existing data series[14–16]. Here we show in an intercomparison exercise that glaciers worldwide have lost 273±16 gigatonnes in mass annually from 2000 to 2023, with an increase of 36±10% from the first (2000−2011) to the second (2012−2023) half of the period. Since 2000, glaciers have lost between 2% and 39% of their ice regionally and about 5% globally. Glacier mass loss is about 18% larger than the loss from the Greenland Ice Sheet and more than twice that from the Antarctic Ice Sheet[17]. Our results arise from a scientific community effort to collect, homogenize, combine, and analyse glacier mass changes from in situ and remote-sensing observations. While our estimates are in agreement with findings from previous assessments[14–16] at a global scale, we found some large regional deviations due to systematic differences among observation methods. Our results provide a refined baseline for better understanding observational differences and for calibrating model ensembles[12,16,18], which will help to narrow projection uncertainty for the 21st century[11,12,18].”

-The bold-faced summary paragraph should contain a sentence beginning “Here we show…” containing the main conclusion. I suggest moving and rephrasing the sentence beginning “The Glacier Mass Balance Intercomparison Exercise” to after the “Here we show” sentence. You could begin with something like “Our results arise from …” or similar.

Response: We revised the summary paragraph to include the “Here we show …” sentence (see above).

-Limit the main text references to no more than 50 and place the remainder in a new Methods References section.

Response: Done.

-If available, provide the DOI for the Zenodo repo.

Response: In the Data availability statement, we provide the DOI for the GlaMBIE dataset (https://doi.org/10.5904/wgms-glambie-2024-07) shared through the WGMS data repository. And in the Code availability statement, we provide the link to the GlaMBIE code in GitHub (https://github.com/earthwave/glambie) as well as the DOI (https://doi.org/10.5281/zenodo.14186227) and reference (Jakob et al. 2024, Zenodo) to the corresponding code release in Zenodo.

-Organize your paper as: title page, text, references, tables, figure legends, methods, data and code availability statements, methods references, acknowledgements, author contributions, author information (containing interest declaration and corresponding author line), Supplementary Information line, and extended data legends.

Response: Done.

Our usual guidelines now follow, many of which you have seen before. Please check through once more.

Please note that the presentation of a Nature paper has undergone significant changes in recent years to improve its readability and navigability online. Specifically, in most instances any supplementary text and data figures/tables can now be integrated into the main paper rather than presented in a separate Supplementary Information file. An overview of the key features and differences may be found in the Composition of a Nature Paper (http://s3-service-broker-live-19ea8b98-4d41-4cb4-be4c-d68f4963b7dd.s3.amazonaws.com/uploads/ckeditor/attachments/7824/3g_Paper_composition.pdf).

Response: We checked the revised composition and organized our features accordingly.

Please also ensure that you provide production-compatible versions of all figures; see http://s3-service-broker-live-19ea8b98-4d41-4cb4-be4c-d68f4963b7dd.s3.amazonaws.com/uploads/ckeditor/attachments/7822/3c_Final_artwork.pdf for detailed guidelines. If figure files are incorrectly formatted, flattened, or of insufficient resolution, they cannot be used and this will delay final acceptance of your manuscript.

Response: We followed the suggested guidelines for figure formatting.

TRANSPARENT PEER REVIEW
Nature offers a transparent peer review option for new original research manuscripts. We encourage increased transparency in peer review by publishing the reviewer comments and author rebuttal letters if the authors agree. Such peer review material is made available as a supplementary peer review file. Please state in the cover letter 'I wish to participate in transparent peer review' if you wish to opt in, or 'I do not wish to participate in transparent peer review' if you wish to opt out. Failure to state your preference will result in delays in accepting your manuscript for publication. If you wish to opt in to transparent peer review please provide your response to reviewers as a .doc file where possible.

Response: We wish to participate in transparent peer review.

Please note: we allow redactions to authors' rebuttal and reviewer comments in the interest of confidentiality. If you are concerned about the release of confidential data, please let us know specifically what information you would like to have removed. Please note that we cannot incorporate redactions for any other reasons. Reviewer names will be published in the peer review files if the reviewer signed the comments to authors, or if reviewers explicitly agree to release their name. For more information, please refer to our FAQ page.

Response: We have no concerns about the release of our rebuttal to reviewer comments.

ORCID: IMPORTANT: All authors identified as 'corresponding author' on the manuscript must have an ORCID associated with their Nature account before submitting the final version of the manuscript. While non-corresponding authors do not have to link their ORCIDs, they are encouraged to do so. Please note that it will NOT be possible to add/modify ORCIDs at the proof stage. Thus, if they wish to have their ORCID added to the paper they must follow the above

procedure prior to acceptance. If you have any issues attaching an ORCID identifier to your MTS account, please contact the Platform Support Helpdesk.

Response: Michael Zemp – as corresponding author – is registered with his ORCID (0000-0003-2391-7877) in his Nature account. I have encouraged all co-authors to register their ORCIDs with their Nature accounts.

SUMMARY PARAGRAPH: All Nature papers begin with a fully referenced paragraph, typically no longer than 200 words, aimed at readers in other disciplines. This paragraph starts with a 2- to 3-sentence, basic introduction to the field; continues with a 1-sentence statement of the main findings starting 'Here we show' or an equivalent phrase; and finally, concludes with 2 to 3 sentences putting the main findings into general context so it is clear how the results described in the paper have moved the field forward. A downloadable, annotated example is available at https://www.nature.com/nature/for-authors/formatting-guide. In some cases it may be necessary to exceed this limit in order to explain complex material for readers in other fields – in such cases, summary paragraphs can be up to 230 words in length. The extra length, however, is for introduction and context, and not for additional technical information.

Response: We follow the suggested structure of the summary paragraph. After the revisions based on feedback from the Editor (see above), its length is 230 words.

MAIN TEXT: If further introductory material is necessary, the main text can begin with up to 500 words of introduction expanding on the background to the work (some overlap with the summary paragraph is acceptable), before proceeding to a concise, focused account of the new research and findings, and ending with 1 or 2 short paragraphs of discussion. Sections are separated with subheadings to aid navigation. Subheadings may be up to 40 characters (including spaces).

Response: We follow the suggested structure and guidelines of the main text.

STATISTICS: Authors should ensure that any statistical analysis used is sound and that it conforms to the journal's guidelines (see https://www.nature.com/nature/for-authors/formatting-guide for guidance).

Response: Our statistical analysis conforms with the journal's guidelines.

METHODS: At the end of the main text document (after the main figure legends), there should be a section entitled "Methods", which provides a more detailed discussion of the additional methodological information that would allow other researchers to replicate the results (we define "Methods" quite broadly, so this is not limited to details of experimental protocols – supplementary discussion and analysis can also be included). The Methods section will not appear in the print version but will be fully copy-edited and appear online in the full-text HTML and PDF versions. The Methods section should be written as concisely as possible but should contain all elements necessary to allow interpretation and reproduction of the results. If there are additional references in the Methods section, their numbering should continue from the last reference in the main paper, and the list should follow the Methods section.

Response: We follow the suggested structure and guidelines of the Methods section.

REFERENCES: As a guideline, Articles allow up to 50 references in the main text; additional references can be cited in (and listed after) the Methods section, as detailed above.

Response: Our article has 50 references in the main text and an additional 64 references in the Methods section.

MAIN TEXT STATEMENTS: We require authors to provide a detailed Author Contribution statement immediately after the acknowledgements; the specific contributions of each author must be listed. It is also a condition of publication that authors include an Author Information statement indicating how to access information regarding reprints and permissions, stating whether or not there is a financial or non-financial competing interest, and naming the author to whom correspondence and requests for materials should be addressed. Please ensure that this section is included in the manuscript file after the Methods (but before the Extended Data legends) - it will not appear in the print version but will appear online in the full-text HTML and PDF versions. For details of "end note" style and an example see https://www.nature.com/nature/for-authors/formatting-guide.

Response: We provide a detailed Author Contribution statement, following the Contributor Role Taxonomy (CrediT, Allen et al. 2019), which is graphically shown in SI Figure 22.

DATA AVAILABILITY STATEMENT: All published manuscripts reporting original research in Nature Portfolio journals must include a data availability statement. The data availability statement must make the conditions of access to the “minimum dataset” that are necessary to interpret, verify and extend the research in the article, transparent to readers. This minimum dataset may be provided through deposition in public community/discipline-specific repositories, custom proprietary repositories for certain types of datasets, or general repositories like Figshare, Zenodo and Dryad. Providing large datasets in supplementary information is strongly discouraged and the preferred approach is to make data available in repositories. More information on Nature Portfolio’s reporting standards and preparing your Data Availability Statement can be found here: https://www.nature.com/nature-portfolio/editorial-policies/reporting-standards#reporting-requirements

Response: In the Data Availability Statement, we provide all data submissions to and results from GlaMBIE as dataset with a DOI (https://doi.org/10.5904/wgms-glambie-2024-07) from the World Glacier Monitoring Service. The registration of the DOI is currently in process and will be completed with the publication of the article.

For all studies using custom code or mathematical algorithm that is deemed central to the conclusions, a statement must be included under the heading "Code availability", indicating whether and how the code or algorithm can be accessed, including any restrictions to access. Code availability statements should be provided as a separate section after the data availability statement but before the References. Code should be deposited in a DOI-minting repository such as Zenodo, Gigantum or Code Ocean and cited in the reference list. Authors are encouraged to

manage subsequent code versions and to use a license approved by the open source initiative. Additional details can be found here: https://www.nature.com/nature-research/editorial-policies/reporting-standards#availability-of-computer-code.

Response: In the Code Availability Statement, we provide the link to the GlaMBIE code in GitHub (https://github.com/earthwave/glambie) as well as the DOI (https://doi.org/10.5281/zenodo.14186227) and reference (Jakob et al. 2024, Zenodo) to the corresponding code release in Zenodo.

FIGURE LEGENDS: These should be listed sequentially after the references in the main text and not in the figures files. Each legend should begin with a brief title for the whole figure and continue with a short description of each panel and the symbols used. Any error bars in the figures must be defined (for example, s.d., s.e.m.) and the value of n indicated; see https://www.nature.com/nature/for-authors/formatting-guide for further explanation.

Response: We followed the suggested guidelines for figure legends. In addition, we added the following statement at the end of the Methods section: "We note that uncertainties (σ) in the Methods section (above) are formulated at the one-sigma level (i.e., 68% confidence interval) to simplify equations, while results in the main text, figures, and tables are reported at the 1.96-sigma level (i.e., 95% confidence interval)."

DISPLAY ITEMS: We ask that you take stock of all the data that have been generated throughout the review process and ensure that only the data most central to the conclusions are presented in the main figures. Figures should be comprehensible to readers in other or related disciplines, and assist their understanding of the paper. We encourage authors who are describing complex processes to include a schematic of the main finding as part of the Extended Data to aid readers unfamiliar with the immediate discipline. Figures should be as small and simple as is compatible with clarity. All panels of a figure should be logically connected; each panel of a multipart figure should be sized so that the whole figure can be reduced by the same amount and reproduced on the printed page at the smallest size at which essential details are visible. For guidance, Nature's standard figure sizes are 89 mm (one column), 120 mm (one and a half columns), or exceptionally 183 mm (two columns) wide; the full depth of a Nature page is 247 mm. All panels of figures should be presented on a single page and assembled into a rectangular shape for publication; please indicate any essential alignments (parts horizontal, vertical, spacings of stereo pairs, etc.). Tables should be prepared using the Table menu in Microsoft Word.

Response: We followed the suggested guidelines for display items.

THIRD PARTY RIGHTS: You must provide proof that you have secured permission to use any third party materials that appear in any part of your manuscript, including extended data and supplementary information. Please fill out a Third Party Rights Table, and upload this to our manuscript tracking system with the final version of your manuscript. Third party materials include any figures, tables, images, videos or text boxes that are reproductions or adaptations of items that have previously been published elsewhere and/or are owned by a third party. This

includes pictures taken by professional photographers, maps and images downloaded from the internet. You will need to obtain the right to use each of these items before your paper can be accepted for publication. You will also need to give proper attribution to the copyright holders in your paper. Please ensure you upload any necessary grants of rights alongside the final version of your manuscript. More information is available on our Rights and permissions page.

Response: Our manuscript uses only open-source material, including proper citation, and does not use content with third-party rights.

COVER ARTWORK: We welcome submissions of artwork for consideration for our cover. More information can be found in our guide for cover artwork. The file name(s) should include the manuscript reference number and be labelled as a cover suggestion; a short description is also preferred. Illustrations should be selected more for their aesthetic appeal than for their scientific content. We cannot promise that your suggestions will be selected for the cover, as competition is intense.

Response: We provide a suggestion for a cover artwork (see below). In addition, ESA offered support to generate a rendering of a glacier landscape with overflying satellites. Please let us know if the timeline towards publication would allow to work on such an additional/alternative suggestion for a cover artwork.

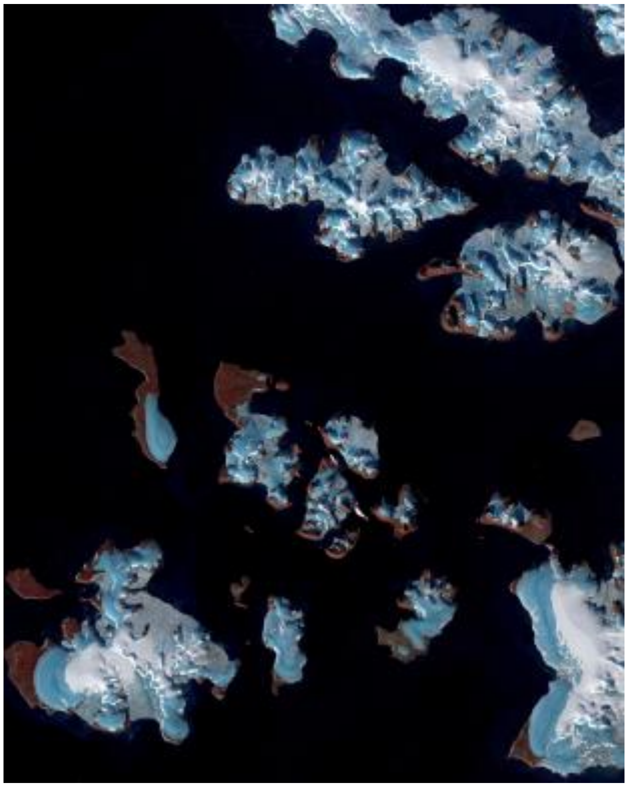

Melting glaciers in Russia's Arctic region. This image, taken by the Sentinel-2 satellite on September 12, 2017, shows the blue glaciers on the reddish-brown Franz Josef Land archipelago north of 80 degrees latitude in the Arctic Ocean (black). The glaciers (blue) are covered with little or no snow (white), which is a clear indication of significant mass loss. Source: Copernicus Sentinel data 2017.

FIGURE FORMATTING: Lettering in all figures (labelling of axes and so on) should be in uniform, sans-serif font, in lower-case type, and large enough to permit substantial reduction for

publication (minimum font size 5 pt). Separate parts of a figure are labelled a, b, etc. Units have a single space between the number and the unit, and follow SI nomenclature or the nomenclature common to a particular field. Thousands are separated by commas (1,000). Unusual units or abbreviations are defined in the legend. Scale bars rather than magnification factors should be used.

Response: We followed the suggested guidelines for figure formatting.

IMAGE PRESENTATION: Authors should be aware that any image provided for publication, either in print or online (as Extended Data or Supplemental Information), may be subject to a quality control process to check for image integrity and manipulation. For a full discussion of our standards regarding how images should be prepared and presented, see www.nature.com/authors/editorial_policies/image.html.

Response: Our article has no images.

EXTENDED DATA: Nature integrates the supplemental figures and tables into the final version of most papers. Extended Data do not appear in the printed version of the paper but are included online within the full-text HTML and at the end of the online PDF. Extended Data are an integral part of the paper and only data that directly contribute to the main message should be presented. All Extended Data must be referred to in the main text, figure legends and/or Methods section, and their figure legends should be listed sequentially at the end of the main text, not in the Extended Data files. Authors should assemble the Extended Data into a maximum of ten, multi-panelled display items, submitted as individual JPEG, TIFF or EPS files. They must be provided at the same quality as figures for print, but there are important differences in their formatting. More specific instructions can be found in the Extended Data Formatting Guide (http://s3-service-broker-live-19ea8b98-4d41-4cb4-be4c-d68f4963b7dd.s3.amazonaws.com/uploads/ckeditor/attachments/7823/3h_Extended_data.pdf).

Response: We followed the suggested guidelines for extended data.

SUPPLEMENTARY INFORMATION: Supplementary Information is online-only material published with the manuscript (https://www.nature.com/nature/for-authors/supp-info). For most papers, there should be no need for Supplementary Information beyond that already provided as Methods and Extended Data, the aim being to avoid unnecessary fragmentation of the paper online. Exceptions to this rule include large datasets that cannot be accommodated within Extended Data; video material; and more complex “Supplemental Methods” (and any associated references) that do not readily fit within the constraints of the Methods/Extended Data formats. Please note that after the paper has been formally accepted you can only provide amended Supplementary Information files for critical changes to the scientific content, not for style. You should clearly explain what changes have been made if you do resupply any such files.

Response: We followed the suggested guidelines for supplementary information and made one deviation. Given the limited number of Extended Data features, we provided full-page figures of our input data and results for all 19 glacier regions as Supplementary Information Figures 1-19. We believe that these figures are important to document the full transparency of our results.

SOURCE DATA: To further increase transparency, we encourage authors to provide, in spreadsheet form, the data underlying the graphical representations used in the figures. This is in addition to our well-established data-deposition policy for specific types of experiments and large datasets. Readers of the online manuscript will be able to access the Source Data directly from the figure legend. Spreadsheets can only be submitted in .xls, .xlsx or .csv formats. One file per figure is permitted; thus, if there is a multi-panelled figure the Source Data for each panel should be clearly labeled in the csv/Excel file; alternatively the data for a figure can be included in multiple, clearly labeled sheets within an Excel file. File sizes of up to 30 MB are permitted; however, it is expected that the vast majority of Source Data files will be considerably smaller than this. When submitting these files with your manuscript, please select the file type "Source Data" and use the title field in the file description tab to indicate the figure to which the Source Data pertain.

Response: we provide source data for all main Figures and Extended Data Figures, except for ED Fig. 2, which shows the GlaMBIE workflow. The source data is organised in one Excel file with separate worksheets per figure.

CONSORTIA: For papers containing one or more consortia, all members of the consortium who contributed to the paper must be listed in the paper (i.e., print/online PDF). If necessary, individual authors can be listed in both the main author list and as a member of a consortium listed at the end of the paper. When submitting your revised manuscript via the online submission system, the consortium name should be entered as an author, together with the contact details of a nominated consortium representative. See https://www.nature.com/authors/policies/authorship.html for our authorship policy and https://www.nature.com/documents/nr-consortia-formatting.pdf for further consortia formatting guidelines, which should be adhered to prior to acceptance.

Response: Our article is submitted by 'The GlaMBIE Team', with all team members listed after the references of the main text. According to our understanding of the guidelines, our article does not contain one or more consortia.

We will not send your revised paper for further review. If the revised paper is in Nature format (as detailed above), in accessible style and of appropriate length, we shall start the acceptance process immediately.

Response: We revised our article following Nature format to the best of our knowledge and believe.

In order to accept your paper, we shall need the following electronic files:
* A cover letter describing your response to our editorial comments and detailing any format changes during revision, particularly if the overall length is affected.

Response: We provide a cover letter.

* A point-by-point response to the remaining issues raised by our referees (a word document is preferred).

Response: We provide a point-by-point reply.

* The final version of your text as a Word document (Word Equation Editor/MathType should be used only for formulae that cannot be produced using normal text or Symbol font). If this is not possible, please provide the manuscript as a single plain vanilla TeX or LaTeX file that includes all references and abbreviations, with no special formatting, as well as a PDF version that is uploaded as a 'related manuscript file'.

Response: We provide our final manuscript as a Word document.

* Production-quality versions of all figures (for details see http://s3-service-broker-live-19ea8b98-4d41-4cb4-be4c-d68f4963b7dd.s3.amazonaws.com/uploads/ckeditor/attachments/7822/3c_Final_artwork.pdf). As we must be able to edit the figures so that they conform to our house style, the submission of files that are incorrectly formatted, flattened, or of insufficient resolution may delay final acceptance of your manuscript.

Response: We provide our figures as PDF files.

* The final version of the Extended Data; this should be presented as individual JPEG, TIFF or EPS files.

Response: We provide our Extended Data Figures and Tables as JPEG files.

* The final version of the Supplementary Information; this should be presented as one file if feasible, ideally a PDF.

Response: We provide our Supplementary Information as PDF file.

* The Source Data, if provided.

Response: We provide our Source data as XLSX file with one worksheets for each ED item.

* Completed and signed copy of the manuscript checklist (https://www.nature.com/documents/nature-manuscript-checklist-research.pdf).

Response: We provide a signed manuscript checklist.

* Completed copy of the editorial policy checklist (https://www.nature.com/documents/nr-editorial-policy-checklist.pdf)

Response: We provide a completed copy of the editorial policy checklist.

* Completed and signed copy of the colour charge form (https://www.nature.com/documents/nature-colour-figure-form.pdf). Nature requests that authors of accepted manuscripts contribute towards the total cost of reproduction of colour figures in print.

Response: We provide a completed and signed copy of the colour charge form.

NOTE: These last two forms should be uploaded as scanned PDFs as a separate attachment, by choosing the file type 'Related Manuscript File'.

Response: Done.

Nature has now transitioned to a unified Rights Collection system which will allow our Author Services team to quickly and easily collect the rights and permissions required to publish your work. Once your paper is accepted, you will receive an email in approximately 10 business days providing you with a link to complete the grant of rights. If you choose to publish Open Access, our Author Services team will also be in touch at that time regarding any additional information that may be required to arrange payment for your article. If you have any questions please contact ASJournals@springernature.com.

Response: Understood.

Nature is now a Transformative Journal. Authors may publish their research with us through the traditional subscription access route or make their paper immediately open access through payment of an article-processing charge (APC). Authors will not be required to make a final decision about access to their article until it has been accepted. Find out more about Transformative Journals.

Response: Understood.

Authors may need to take specific actions to achieve compliance with funder and institutional open access mandates. If your research is supported by a funder that requires immediate open access (e.g. according to Plan S principles) then you should select the gold OA route, and we will direct you to the compliant route where possible. If you select the subscription publication route our standard licensing terms will need to be accepted, including our self-archiving policies. Those standard licensing terms will supersede any other terms that the author or any third party may assert apply to any version of the manuscript.

Response: We select the gold OA route. Please note the special requirements by USGS as mentioned below, at the end of our point-by-point reply.

All of the electronic files should be uploaded using the following link: [link removed]

We hope to hear from you within two weeks; please let us know if the process may take longer.

Yours sincerely

Michael

Dr Michael White
Senior Editor, Nature
San Francisco
ORCID iD 0000-0002-0238-8913

+ +

Referee #2 (Remarks to the Author):

The authors have apparently made substantial effort trying to improve the manuscript and clarify reviewers' comments and questions. Even though my main concerns remain more or less the same (the novelty of the conclusions and the suitability of such a comprehensive and complicated analysis to the broad general audience of Nature), I will trust and support the editors' decision.

Response: We thank Referee #2 for the positive feedback, which improved the clarity of our paper.

Here are a few comments that the authors may consider to further improve the manuscript.

1) Extended Data Figure 1 is still too busy and very confusing. I didn't see much difference compared to the original version.

[Editor suggestion: "1) You are welcome to further revise ED figure 1, but this is not required."]

Response: We acknowledge that Sankey diagrams might not be to everybody's taste. However, the vast majority of co-authors found them useful for providing an overview of a comprehensive intercomparison exercise. Hence, we believe they will be also useful for readers and decided to keep ED Figure 1 unchanged.

2) Supplementary Data Table 1 can be better organized. A whole page is occupied by the affiliation information of the WGMS team. There is no need to provide all the information here in the table (providing a reference is enough) and break the table into two separate parts.

[Editor suggestion: "2) If you see a clear way to simplify SI table 1 you are welcome to do so."]

Response: Given the tremendous effort required to carry out, compile, and share all the in situ observations, we do not believe that deleting the affiliation information of the WGMS staff, national correspondents, and principal investigators in SI Table 1 is justified. Hence, we decided to keep the names and affiliations of all data contributors.

However, we separated the information into two SI tables for better readability. Newly, we have SI Table 1, which provides an overview of methods, sensors or products, method references, and

regions for each research team, and SI Table 2, which provides the affiliations for each research team.

3) Time spans of different inputs from different groups for the same technique should be also given here in Supplementary Data Table 1.

[Editor suggestion: "3) If the requested time spans are available elsewhere you do not need to add them to SI table 1."]

Response: Providing information about temporal coverage in SI Table 1 is not really feasible since it would have to include temporal resolution and gaps and since this information differs for each research group, method, and region.

However, we added a sentence to the caption of SI Table 1, pointing out that "Information about temporal coverage, resolution, and gaps of the submitted data are shown for each region in SI Figures 1−19."

4) Some of the inputs only cover a short period of time (e.g., Gravimetry from Jacob et al., 2003-2011 and Colgan et al., 2002-2009). It would be more helpful to get some updated series. If the authors only want to get an ensemble average of previously published series (regardless the time spans), there should be many more other published series available.

[Editor suggestion: "4) If you see a straightforward way to extend your time series or add additional time series you are welcome to do so. Again, doing so is not required."]

Response: GlaMBIE is a community effort which compiles observations through an open call for data. While some research groups used this opportunity to improve and/or update their results from earlier studies, others only had the resources to submit results from earlier work. To clarify this point, we added a corresponding sentence in the first paragraph of the Methods section: "Data contributions were compiled through an open call for data submission and selected based on expert evaluation of their confidence levels."

With this approach, GlaMBIE compiled the observational estimates from previous IPCC reports and activated the community to update previous studies, extend them to additional regions, and produce new estimates. We are confident in the ability to further improve the spatial and temporal coverage from all methods in a subsequent phase of GlaMBIE.

5) The authors may want to use consistent colors/formats for the Supplementary Data Figures. Some of the bottom panels (Gravimetry) are in b/w, while most of others are in color.

[Editor suggestion: "5) Please harmonize your color schemes where appropriate."]

Response: SI Figures 1−19 come with consistent colors and formats. As noted in the caption ("Description of Supplementary Data Figures 1−19"), "datasets not used are shown as grey dotted lines".

+ +

Referee #3 (Remarks to the Author):

The authors have done a thorough job at responding to reviewer and editorial feedback, and the new sections and figures improve the paper. The new text (from lines 83 -92) is certainly much clearer.
I still don't believe that the regional estimates will be useful for model evaluation (given that this is mostly done at glacier scale), but the community approach is noteworthy and reproducible, and results are important for understanding regional mass changes.

Response: We thank Referee #3 for the positive feedback, which improved the clarity of our paper.

However, in re-reading the entire revised manuscript I have become concerned about the description of uncertainties and interpretation of results considering the uncertainty bounds. Even though this seems to be more of an issue of presentation than substance, I'd like it to be corrected before the paper can be considered for publication.

Response: We agree that more guarded wording can help to present the results in the right context and, hence, implemented the related suggestions as outlined below.

Starting with the revised abstract -

While our results generally confirm the findings from previous assessments at a global scale, we found some large regional differences, partly within random errors, suggesting a systematic error among observation methods.

- as well as being difficult to read ('partly within random errors'), this sentence is concerning because differences within uncertainty bounds cannot be interpreted as meaningful.

Response: We disagree with the statement of Referee #3 that "differences within uncertainty bounds cannot be interpreted as meaningful". First, the statistical interpretation of a result depends on both the signal and the random noise. Hence, a result can be meaningful but not statistically significant due to either a small signal, a larger observational uncertainty, or both. Second, a result might be subject to a systematic error not captured by the random uncertainty. Hence, a systematic error can be meaningful but might only become statistically significant after cumulating over a long enough observation period. Third, the significance of a difference depends on the confidence level. Even if a difference is not significant with a 95% confidence level, it is significant with at a lower confidence level. Given the strong implications of sea-level rise, even such a difference may be important to consider.

However, we agree with Referee #3 that the sentence in the abstract is difficult to read and, hence, revised it for improved clarity:

“While our results are in agreement with the findings from previous assessments[14–16] at a global scale, we found some large regional deviations due to systematic differences among observation methods.”

In the main text, in the section on Comparison to IPCC estimates, we then briefly discuss the issue of uncertainty levels:

“At a regional level, the differences to the results from AR6 range between −20% for the Southern Andes and +53% for the Antarctic & Subantarctic Islands but are often within uncertainties (Extended Data Table 3). Even if these differences are not always significant at the 95% confidence level, they are in many cases large enough to be worth considering given the severe impacts of glacier changes.”

Looking at this further within the manuscript -

Line 225. At a regional level, the differences to the results from AR6 range between −20% for the Southern Andes and +53% for the Antarctic and Subantarctic Islands AND are often within uncertainties (Extended Data Table 3).

AND needs to be replaced with BUT in this sentence. The fact that the differences are within uncertainties limits what the authors can interpret about these findings.

Response: Done. We replaced AND with BUT in this sentence.

From Line 225 -
‘GlaMBIE extends the temporal coverage to 2023, provides results at annual resolution, and has established a community mechanism to allow estimates to be updated towards IPCC AR7. Regarding uncertainties, our estimates come with error bars that are smaller than the ones reported in IPCC SROCC which the improvement in observational coverage can explain. Compared to IPCC AR5 and AR6, our estimates feature similar uncertainties over multi-year periods but additionally have the advantage of providing results and uncertainties at annual resolution.’

Although it is true that the uncertainties are smaller, the authors also state earlier in the paper that ‘Since true independence among these mass-change estimates is unlikely, our random errors will likely represent a lower range of the actual uncertainty for a given region.’

All of these statements need to be reconciled to avoid giving the impression of overinterpretation.

Response: We do not see an overinterpretation in our statements: we point out where results are within error bars and discuss the limitations of our uncertainty assessment at the end of the sections “Glacier mass balance intercomparison” and “Differences among observation methods.” Please note that the mentioned limitations in the uncertainty assessment apply not only to our study but also – for example – to the results presented and compared in the IPCC reports. Thus,

if our random errors likely represent a lower range of the actual uncertainty, the same applies to corresponding results in the IPCC reports.

Perhaps the authors can just highlight areas where there are very large differences from previous assessments? For example, the results for peripheral glaciers in Antarctica are very different to previous estimates and this is noteworthy and could receive more attention given that it seems to be for good reason (inclusion of altimetry results).

Response: In the main text, section "Comparison to IPCC estimates and outlook", we discuss areas of larger differences and provide reference to ED Tables 3 and 4 containing the differences for all regions:

"At a regional level, the differences to the results from AR6 range between −20% for the Southern Andes and +53% for the Antarctic & Subantarctic Islands but are often within uncertainties (Extended Data Table 3). Even if these differences are not always significant at the 95% confidence level, they are in many cases large enough to be worth considering given the severe impacts of glacier changes. The values reported by IPCC are based on a few selected studies per region. In contrast, our GlaMBIE protocols (Extended Data Figure 1) consistently combine time series from the main observation methods, each based on input from multiple research teams. As such, our coordinated community effort yields a most comprehensive estimate of glacier mass changes from observations. The annual resolution of the GlaMBIE estimates represents an important improvement over the multi-annual change rates reported by the IPCC. Over aggregated regions (Extended Data Figure 4), we found the most prominent differences for Greenland Periphery and for Antarctic & Subantarctic Islands, where our estimate (-53 ± 11 Gt yr^{-1}) is less negative than reported in IPCC AR6 (-61 ± 4 Gt yr^{-1}) due to our inclusion of results from altimetry; and in regions with a small glacier area, where our estimate (-17 ± 1 Gt yr^{-1}) is also less negative than reported in IPCC AR5 (-25 ± 4 Gt yr^{-1}) due to our exclusion of results from gravimetry."

Minor suggestions:

Could this sentence which interrupts the flow of the paragraph -

225. 'Reference data at the glacier scale, such as from airborne laser surveys, are less prone to errors than space-borne observations and can be used for validation.'

be removed or added at the end of this paragraph using 'For example, …'?

Response: Done. We moved the sentence to the end of the paragraph using "For example, …".

Iine 307. 'gas emissions and associated warming to limit the impacts of glacier vanishing down-wasting on local geohazards, regional freshwater availability, and global sea-level rise.'

- Glacier downwasting implies only vertical change whereas glaciers are thinning and retreating. Suggest replacing ' glacier down wasting' with ' glacier wastage'.

Response: Done. We replaced “glacier down wasting” with “glacier wastage”.

+ +

Additional improvements

During the revision of the manuscript, we implemented the following additional improvements, listed here for completeness and transparency:

Additional method references: We added an additional method reference to Supplementary Data Table 1 and updated our reference list accordingly: Nilsson et al., Altimetry: Nilsson et al. (2022), Nilsson and Gardner (2024).

Updated author affiliations: We updated the affiliations of the authors, adding a second institution to T.B.: “[12]Central-Asian Regional Glaciological Centre of Category 2 Under the Auspices of UNESCO, Almaty, KZ.”

Additional minor changes: Based on co-author feedback during the revision, we made minor changes to the manuscript (mainly rewording) to improve clarity and readability.

English editing: We polished the English of the manuscript with the support of native English-speaking co-authors.

All changes are reported in the track-change document.

+ +

Additional matters

USGS requirements about license agreement: One of the co-authors (Caitlyn Florentine) is an employee of the U.S. Geological Survey (USGS) and needs to be sensitive to governmental policies regarding license agreements. To that end, USGS asked to point out the following requirements for Caitlyn Florentine to be listed as a co-author:

+ The USGS receives an Adobe PDF agreement, so her Center Director can securely sign it instead of using DocuSign or similar software, which is not permitted.

+ The USGS will need to sign a separate agreement that contains only the USGS author.

+ If Nature uses the standard Springer Nature open access agreement, please incorporate the following edits:

Section 5 “Cooperation: "The Author will cooperate fully with the Licensee in relation to any legal action that might arise from the publication of the Article, and the Author will give the Licensee access at reasonable times to any relevant accounts, documents, and records within the power or control of the Author, **provided that the Author's access or cooperation with**

Licensee or any of its affiliates does not violate the federal laws, regulations or policies of the United States of America. The Author agrees that any Licensee affiliate through which the Licensee exercises any rights or performs any obligations under this Agreement is intended to have the benefit of and will have the right to enforce the terms of this Agreement."

Provision 9 "Governing Law": "This Agreement will be governed by, and construed in accordance with, the federal laws of the United States of America. The federal courts of the United States of America will have exclusive jurisdiction."

Caitlyn Florentine (cflorentine@usgs.gov) and her USGS science center communication officer are available to support the implementation of the license agreement with USGS.

If implementing such a separate license agreement with USGS is not feasible, I propose, as corresponding author, that Caitlyn Florentine be removed from the author list, but her contribution to GlaMBIE is recognized in the acknowledgements.

Version 0:
Referee #2

This manuscript provides a fairly comprehensive and lengthy analysis of global glacier mass changes from 2000 to 2023 based on inputs from different techniques and a large number of groups. There is no doubt that the study is important and should be published. My main concern is that if the results and presentations are fitted to Nature. I understand this intends to follow the footsteps of the two IMBIE Team's publications in Nature in 2018 and 2020 on Greenland and Antarctic ice mass balance. However, given the scope of global glaciers and the way the results were organized, it would be very difficult for the general readers to comprehend.

Major comments:

1) The analysis, result presentation, and organization are fairly difficult to follow. As a researcher with over 20 years experience in related fields, I myself had a really hard time to follow the analysis, data processing and results interpretation. This is not necessarily the authors' fault. The target here (global glaciers) is simply too broad.

   The two IMBIE Teams did a good job in putting together two very concise short papers that are both important and can be easily understood by the general readers. Greenland and Antarctic ice sheets are two single targets, with one covered by each of the IMBIE papers. In comparisons, there are 19 target regions in the present analysis.

   The complexity is caused by not only the large number of target regions in the present study, but also the different techniques used for different regions and different groups providing the different estimates.

2) While this community assessment of global glaciers mass balance is important, the main conclusions are not really new and some are mostly a confirmation of a recent Nature paper by Hugonnet et al. (2020) (with its author/co-authors also on this GlaMBIE Team). This is another reason that I believe it would fit better to a more specialized journal.

3) As a main conclusion, the authors claimed that the estimated glacier mass loss rate (-273 Gt/yr) over 2000-2023 is about 18% larger than the loss from the Greenland Ice Sheet and more than twice that from the Antarctic Ice Sheet and used Otosaka et al. (2023) as reference. Otosaka et al. (2023) was a extended analysis of the IMBIE Team paper over a longer period of time 1992-2020. There were no separate Greenland and Antarctic mass rate estimates for the period 2000-2023 in Otosaka et al. (2023). I am not sure what the authors compared with. It could be likely from Figure 3 and corresponding descriptions in Otosaka et al. (2023) for Greenland and Antarctic estimates for the period 2003-2018.

   Nevertheless the comparison is not valid given the different time spans (differing by at least 4-5 years). For unknown reasons, the gravimetry results in Otosaka et al. (2023; Figure 3) for Greenland over the period 2003-2018 (~ -220 Gt/yr) appear significantly smaller than similar GRACE and GRACE-FO estimates, e.g., -261 Gt/yr over the period 2002-2019 from Velicogna et al. (2020).

   An updated gravimetry estimate of Greenland mass rate over the period 2002-2023 can be easily derived from the latest GRACE and GRACE-FO mascon solutions. This GlaMBIE Team includes several experts in satellite gravimetry and it should not be difficult.

4) If I understand correctly, the authors included Greenland Periphery and Antarctic & Subantarctic Islands in the global glacier mass change and then compared the estimated

global glacier mass change with Greenland and Antarctic ice sheets mass change. There might be some inconsistency here, as satellite gravimetry could not separate those small signals from the nearby ice sheets mass changes. This is yet another indication that this analysis may fit better for a specialized journal, as there are not many people (except for glaciologists) who can really tell the difference between Greenland Periphery and Greenland glaciers.

5) Since the inputs from different techniques, different groups and different series from the same group covered different time span, it is not clear how those data were combined. For example, the authors “analysed 78 geodetic assessments from gravimetry from 7 research teams, covering 17 out of 19 regions”. What are those 78 geodetic assessments? Time spans? Targets?

6) Line 27: “with an estimated total volume of $158\pm41\times10^3$ km^3, equivalent to a potential sea-level rise of 32±8 mm”. This is wrong statement. The global ocean area is about 360 million square kilometers, and the 158×10^3 km^3 glacier ice would be equivalent to a potential sea-level rise of 439 mm, more than one order larger than the number (32 mm) given here.

7) Extended Data Figure 1: It is a novel idea to illustrate the data submissions this way, but it is still very confusing, especially when different groups share the same color. There is no way to really track down the actual contributions from the different groups in this case.

8) Extended Data Figure 3: This is a busy graphic. Among the 19 regions, the time spans are mostly different, and none covers the period before 2002. Then how could the authors derive reliable estimates for the period 2000 – 2023? A time span was given for each of the 19 regions, with 4 estimates from the 4 techniques. Did the 4 techniques cover the same time span?

   There are simple too many things to consider and too many issues to deal with in this comprehensive analysis. A more specialized journal should be a better fit for this work.
